# A Small RNA Is Linking CRISPR–Cas and Zinc Transport

**DOI:** 10.3389/fmolb.2021.640440

**Published:** 2021-05-13

**Authors:** Pascal Märkle, Lisa-Katharina Maier, Sandra Maaß, Claudia Hirschfeld, Jürgen Bartel, Dörte Becher, Björn Voß, Anita Marchfelder

**Affiliations:** ^1^Department of Biology II, Ulm University, Ulm, Germany; ^2^Department of Microbial Proteomics, Institute of Microbiology, University of Greifswald, Greifswald, Germany; ^3^Institute of Biochemical Engineering, University of Stuttgart, Stuttgart, Germany

**Keywords:** small RNA, archaea, CRISPR-Cas, zinc transport, haloarchaea

## Abstract

The function and mode of action of small regulatory RNAs is currently still understudied in archaea. In the halophilic archaeon *Haloferax volcanii*, a plethora of sRNAs have been identified; however, in-depth functional analysis is missing for most of them. We selected a small RNA (s479) from *Haloferax volcanii* for detailed characterization. The sRNA gene is encoded between a CRISPR RNA locus and the Cas protein gene cluster, and the s479 deletion strain is viable and was characterized in detail. Transcriptome studies of wild-type *Haloferax* cells and the deletion mutant revealed upregulation of six genes in the deletion strain, showing that this sRNA has a clearly defined function. Three of the six upregulated genes encode potential zinc transporter proteins (ZnuA1, ZnuB1, and ZnuC1) suggesting the involvement of s479 in the regulation of zinc transport. Upregulation of these genes in the deletion strain was confirmed by northern blot and proteome analyses. Furthermore, electrophoretic mobility shift assays demonstrate a direct interaction of s479 with the target *znu*C1 mRNA. Proteome comparison of wild-type and deletion strains further expanded the regulon of s479 deeply rooting this sRNA within the metabolism of *H. volcanii* especially the regulation of transporter abundance. Interestingly, s479 is not only encoded next to CRISPR–*cas* genes, but the mature s479 contains a crRNA-like 5′ handle, and experiments with Cas protein deletion strains indicate maturation by Cas6 and interaction with Cas proteins. Together, this might suggest that the CRISPR–Cas system is involved in s479 function.

## Introduction

Small RNAs have been well established as key regulators of gene expression in both pro- and eukaryotic species (Storz et al., 2011; Wagner and Romby, 2015; Buddeweg et al., 2018a), but still, understanding of small RNAs (sRNAs) in the archaeal domain lags behind (Gomes-Filho et al., 2018). RNomics and the more recent high-throughput approaches have been applied to several archaeal species to uncover the wealth of small transcripts found in this domain of life [reviewed in Marchfelder et al. (2012); Kliemt and Soppa (2017), Buddeweg et al. (2018a), and Gelsinger and DiRuggiero (2018a)]. With numbers in the hundred (*Archaeoglobus fulgidus*, *Methanosarcina mazei*, *Sulfolobus solfataricus*, *Thermococcus kodakarensis*, *Pyrococcus abyssi*, and *Haloferax mediterranei*) or even thousand (*Haloferax volcanii* and *Methanolobus psychrophilus*), sRNAs are well established as widespread and abundant players within the transcriptome of various archaeal species [reviewed in Gelsinger and DiRuggiero (2018a); Gomes-Filho et al. (2018), and Payá et al. (2018)].

sRNAs from the haloarchaeal model organism *H. volcanii* have been studied since more than 10 years (Straub et al., 2009; Babski et al., 2011; Fischer et al., 2011; Heyer et al., 2012). Several recent RNA-seq and differential RNA-seq (dRNA-seq) studies explored the small RNome of *H. volcanii* in greater depth and uncovered an unexpected wealth of potential small RNAs expanding the number of sRNA candidates from just about 200 identified in 2009 to now well over 1,000 candidate sRNAs (Heyer et al., 2012: 190 sRNAs; Babski et al., 2016: 1,701 candidates; Laass et al., 2019: 1,635 candidates; Gelsinger and DiRuggiero, 2018b: 1,533 candidates) (Heyer et al., 2012; Babski et al., 2016; Gelsinger and DiRuggiero, 2018b; Laass et al., 2019). Depending on their genomic localization, small regulatory RNAs are categorized as trans-encoded intergenic sRNAs (sRNAs) or cis-encoded antisense RNAs (asRNAs) overlapping with annotated reading frames of the opposite strand. In contrast to asRNAs, for whom targets are readily deducible as they are by default able to extensively base pair with the transcript originating from the opposite strand, trans-acting sRNAs pose quite a challenge as to the identification of targeted mRNAs. More so, as they bind targets *via* imperfect base-pairing, sRNAs may regulate multiple targets as demonstrated in bacteria (Papenfort and Vogel, 2009; Wagner and Romby, 2015) and by the few archaeal examples [reviewed in Buddeweg et al. (2018a); Gelsinger and DiRuggiero (2018a), and Gomes-Filho et al. (2018)].

It is well established that intergenic sRNAs of *H. volcanii* are differentially expressed in response to growth phase or environmental stimuli including temperature, salinity, and oxidative stress (Straub et al., 2009; Babski et al., 2011; Fischer et al., 2011; Gelsinger and DiRuggiero, 2018a). Their profound biological role is unquestioned as growth phenotypes have been demonstrated for sRNA deletion mutants in response to temperature, salt, alternative carbon sources, phosphate availability, and oxidative stress (Straub et al., 2009; Fischer et al., 2011; Heyer et al., 2012; Jaschinski et al., 2014; Kliemt et al., 2019). Besides growth, swarming behavior or cell shape has been affected by sRNA deletions as well (Jaschinski et al., 2014). sRNA-mediated regulation may involve a range of sRNAs as differential transcriptome analyses in the context of oxidative stress imply (Gelsinger and DiRuggiero, 2018b). A recent metatranscriptome study highlights the importance of sRNA-based regulation for the archaeal metabolism, once more demonstrating the differential expression of sRNAs in a halo-extremophile community inside salt rocks of the Atacama Desert in response to environmental changes on a population-wide scale (Gelsinger et al., 2020). This underpins that sRNAs do not take the sideline in archaeal gene regulation but are central players for stress adaptation in a large-scale perspective. Despite this immense body of evidence as to the involvement of sRNA-mediated regulation in the processes of metabolic and stress adaptation, data on sRNA-target pairs are still scarce.

The intergenic sRNA s479 of *H. volcanii* described herein gained our interest, as sRNA resulting in a growth phenotype upon deletion but also as RNA encoded between genes for the CRISPR–Cas system. Differential transcriptome and proteome analysis revealed changes in several zinc-related mRNAs and proteins, respectively. Analyses of the deletion strain in conjunction with electrophoretic mobility shift data and impaired growth under elevated zinc concentrations confirm a role of s479 within the zinc regulon. Differential proteome analysis reveals a role for s479 in the adjustment of a network of ABC transporters. Furthermore, s479 seems to depend on Cas proteins for maturation and stability.

## Results

### Characterization of s479

**FIGURE 1:**
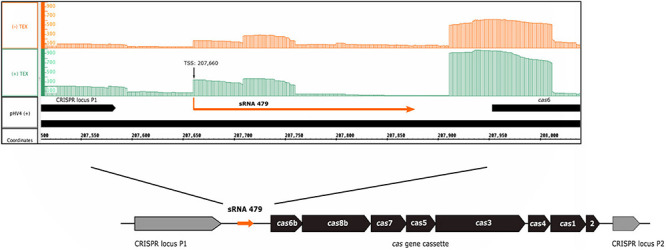
Genomic location of s479. The sRNA 479 is encoded on the genomic plasmid pHV4, and as the read abundance shows (Jaschinski et al., 2014; Babski et al., 2016), transcription initiates at position 207,660. Transcription start site data (TSS) together with the previously obtained RNomics data (Straub et al., 2009) show that the s479 gene is 213 bp long. Location of s479 is noteworthy, as it is encoded in the intergenic region between a CRISPR locus and the *cas* gene cassette. Reads obtained from RNA treated with terminal exonuclease (+TEX) are shown in green, and reads from untreated RNA (−TEX) are shown in orange. Comparison of reads from both fractions allowed us to determine the TSS (indicated by an arrow). Genome coordinates and annotated genes of the main chromosome plus strand are shown in black at the bottom.

The s479 RNA was identified in an early RNomics study, sequencing cDNA clones after RNA size selection (Straub et al., 2009). It is an intergenic sRNA located on the genomic plasmid pHV4 downstream of the CRISPR locus P1 and upstream of the type I-B *cas* gene cassette (Figure 1). A genome-wide high-throughput study analyzing transcriptional start sites of *H. volcanii* (Babski et al., 2016) revealed an enrichment of transcript starts at position 207,660.

**FIGURE 2:**
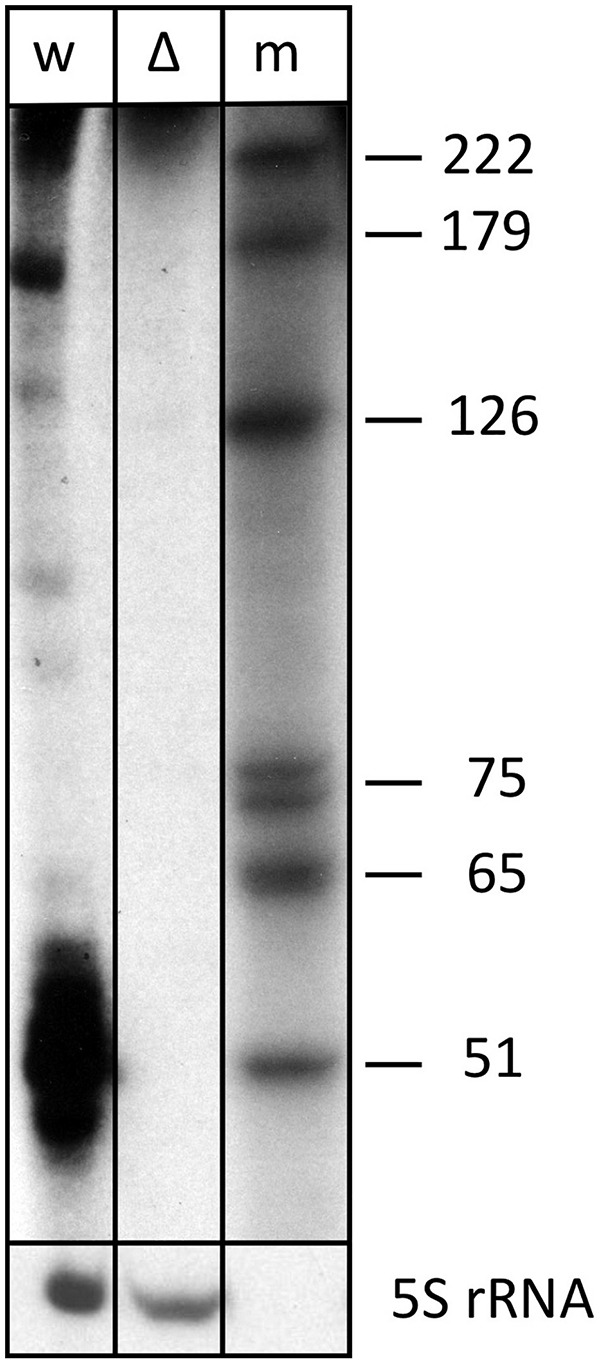
The s479 precursor transcript is processed into a shorter RNA. Total RNA from wild-type (lane w) and Δ*s479* (lane Δ) cells was separated on a polyacrylamide gel. After transfer, the membrane was hybridized with a probe against s479 (upper panel, the lower panel was hybridized with a probe against the 5S rRNA). Lane w: RNA from *H. volcanii* wild-type strain, lane Δ: RNA from a Δ*s479* strain, lane m: size marker; sizes are given at the right in nucleotides.

**FIGURE 3:**
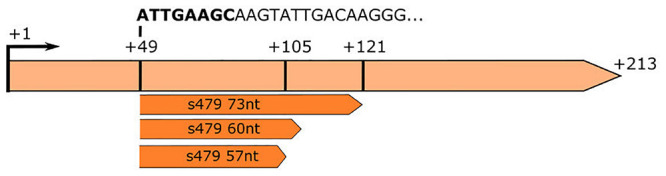
RNA-seq analysis of small RNAs reveals sequences matching the s479 locus. The s479 precursor RNA is 213 nucleotides long, shown in light orange. RNomics analysis of size-selected RNA samples (Maier et al., 2013) reveals shorter versions of s479: 57, 60, and 73 nt in length, shown below in dark orange. These data confirm the results of the northern blot (Figure 2). The sequence of the shorter sRNAs starts at nucleotide 49 of the precursor. The first 8 nucleotides are identical to crRNA 5′ handle sequence (shown in bold: ATTGAAGC) (Supplementary Figure 2).

Expression of s479 was confirmed by northern blot analysis revealing long RNAs of about 220 and 160 nucleotides and a very strong cluster of signals of approximately 51 nucleotides (Figure 2), showing that the primary s479 transcript is processed yielding an RNA of about 51 nucleotides. The northern data confirm the dRNA-seq results presented in Figure 1, which show that RNAs starting at the mature s479 5′ end (position 49 in Figure 3) with about 50 nucleotides length have the most reads.

**FIGURE 4:**
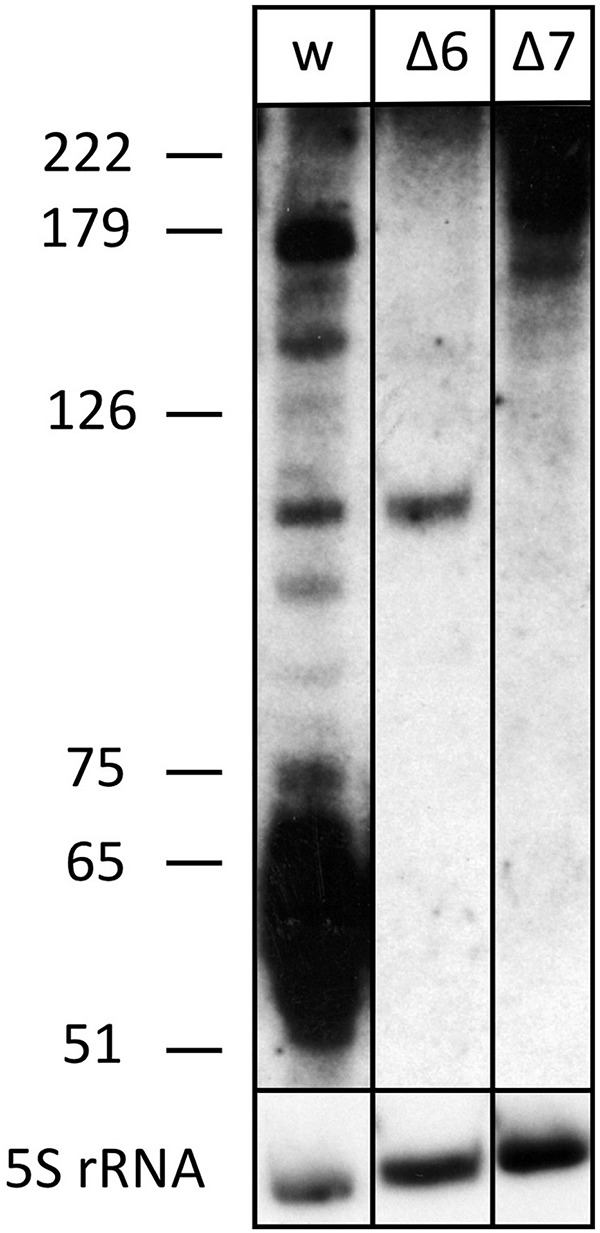
The processed s479 species are lost upon deletion of genes for the Cas6 or Cas7 protein. Total RNA was separated on a polyacrylamide gel. After transfer, a probe against s479 was used for hybridization (upper panel). A size marker is given at the left. Lane w: RNA from *H. volcanii* H119 wild-type strain, lane Δ6: RNA from *H. volcanii cas6* deletion strain Δ*cas6*, lane Δ7: RNA from *H. volcanii cas7* deletion strain Δ*cas7*. In the lower panel, hybridization with a probe against 5S rRNA is shown.

This is also supported by a serendipitous finding: an RNA-seq study of small RNAs to identify CRISPR RNAs revealed reads mapping to the s479 locus; a 57 and a 60 nt as well as a 73 nt form (Figure 3; Supplementary Figure 1; Maier et al., 2013). These correspond in size to the cluster of bands visible in the northern blot analysis (Figures 2, 4).

**FIGURE 5:**
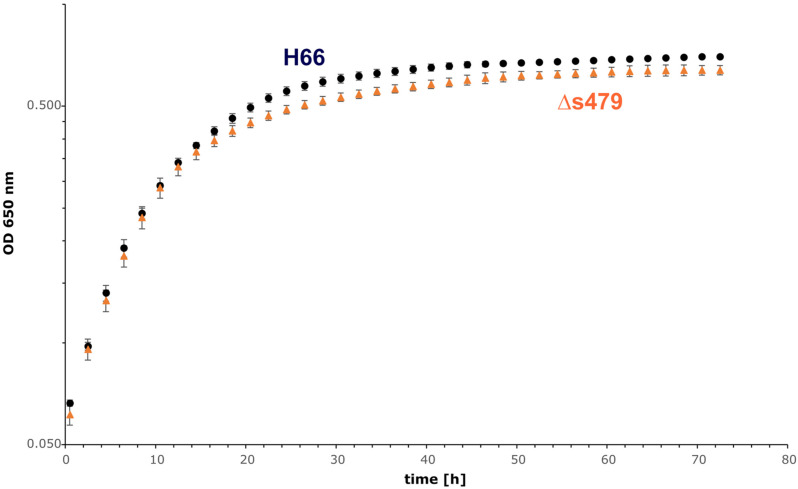
Growth experiment comparing the wild-type strain and Δ*s479* strain. Both strains were cultivated in triplicate with Hv-Ca medium (supplemented with uracil) in microtiter plates and OD_650 nm_ was monitored using a heated plate-reader instrument.

To elucidate the importance of s479 in the context of *H. volcanii* metabolism in more detail, we first compared the growth of the s479 deletion strain (Δ*s479*) obtained in a previous study (Jaschinski et al., 2014) with the H66 wild-type strain (Figure 5). Growth curves show a diauxic growth: doubling times of the wild-type and deletion strains in phase 1 (0.5–10.5 h) are not very different with the doubling times of 4.7 h for the wild-type strain and 4.5 h for the deletion strain (see also Supplementary Figure 5C for doubling time details). However, in phase 2 (14.5–24.5 h), the doubling time of the deletion strain is longer with 24 h compared with 19 h of the wild-type strain. In addition, the deletion strain reaches a lower OD in stationary phase, showing that the sRNA has an important function in the cell.

### Influence of s479 on the *H. volcanii* Transcriptome

As deletion of s479 led to a modest growth phenotype, we wanted to identify genome-wide changes in the *H. volcanii* gene expression profile resulting from loss of sRNA expression. We performed RNA-seq analysis of the sRNA deletion strain Δ*s479* and the wild-type strain H66 grown to exponential phase. To pinpoint transcripts affected by the absence of s479, we applied a stringent differential transcriptome analysis.

**FIGURE 6:**
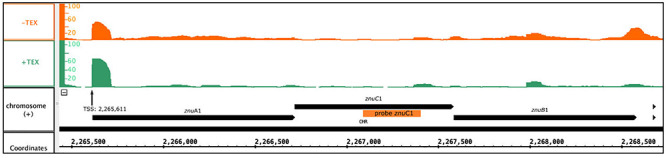
Genomic localization of the *znu* operon. Depicted is the localization of the *znu* operon alongside the read coverage at TSS (Babski et al., 2016) confirming the joint transcription of all three *znu* genes as an approximately 3,000 nt transcript. The location of the PCR probe used for *znu*C1 detection in northern blot hybridization is given as orange bar. Reads obtained from RNA treated with terminal exonuclease (+TEX) are shown in green, and reads from untreated RNA (−TEX) are shown in orange. Comparison of reads from both fractions allowed us to determine the TSS (indicated by an arrow). Genome coordinates and annotated genes of the main chromosome plus strand are shown in black at the bottom.

Bioinformatic analysis identified one transcript as downregulated, the s479 RNA, and five transcripts as upregulated with a log_2_ fold change ≥2. As shown in Table 1, two of the upregulated genes are derived from a single genomic region on the main chromosome comprising among others the operon *znu*A1C1B1 encoding a putative zinc ABC transporter (Figure 6).

**TABLE 1:** Six genes are differentially expressed in the deletion strain Δ*s479*.

**Gene**	**log_2_**	***p* adj**	**Product**
**Downregulated**			
s479	–4.30	2.7E-31	s479
Upregulated			
HVO_2402; *gcv*P2	3.27	1.7E-19	Glycine dehydrogenase
HVO_2396; *grx*4	2.16	6.5E-07	Glutaredoxin-like protein
HVO_2398; *znu*C1	2.11	1.6E-07	Putative zinc ABC transporter ATP-binding protein
HVO_2400	2.08	1.7E-08	Hypothetical protein
HVO_2399; *znu*B1	1.98	3.2E-07	Putative zinc ABC transporter permease

*The log_2_ fold change (column log_2_) deletion vs. wild type is given alongside the HVO–gene number (column gene), *p* value (column *p* adj), and gene product name (column product).*

Abundance of the two transcripts, *znu*C1 and *znu*B1, increases more than four-fold in response to s479 deletion. *znu*A1, encoding the periplasmic substrate-binding protein of the said putative ABC transporter, is also present within the set of differentially expressed genes but fell just below the threshold of log_2_FC ≥2 with a score of 1.8 (Supplementary Table 1).

As the abundance of all three genes of the *znu* operon is altered upon s479 deletion, we further concentrated our analysis on the *znu* operon comprising *znu*A1 (HVO_2397), *znu*B1 (HVO_2399), and *znu*C1 (HVO_2398). TSS analyses (Babski et al., 2016) show that expression is governed by a single TSS four nucleotides upstream of the *znu*A1 start codon, resulting in a multicistronic mRNA of approximately 3,000 nt (Figure 6). Such a short 5′ UTR is typical for *H. volcanii* which has a high percentage of leaderless mRNAs and 5′ UTRs shorter than six nucleotides (Babski et al., 2016).

**FIGURE 7:**
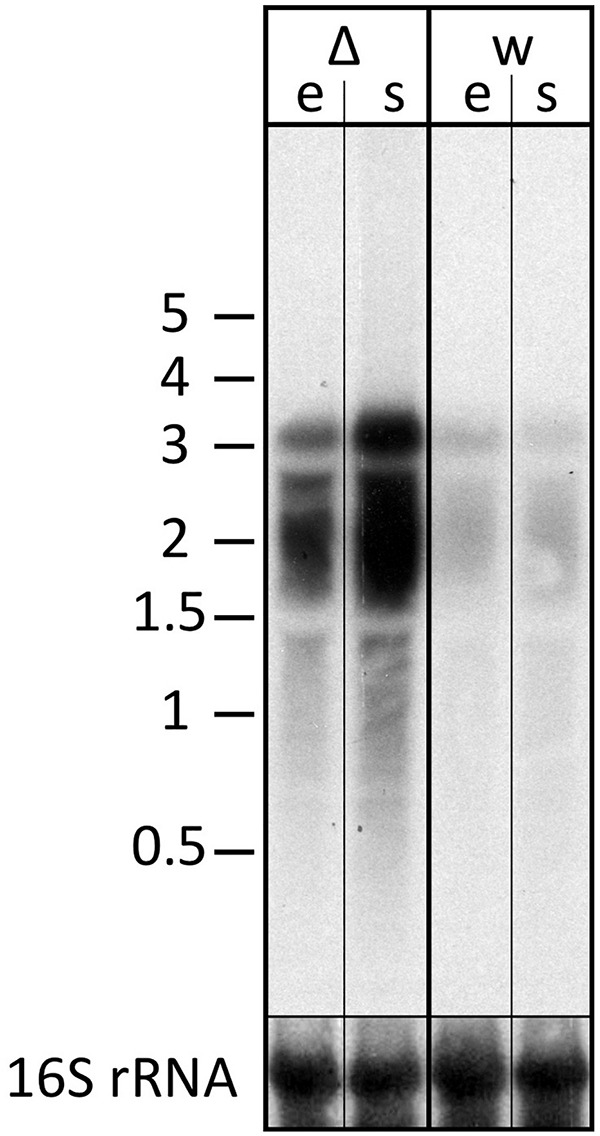
Transcript levels of the *znu* operon in wild-type and s479 deletion strains. We compared the transcript levels of *znu* in total RNA of wild-type H66 (lanes w) and Δ*s479* (lanes Δ) cells at exponential and stationary phases (lanes e and s, respectively) using northern blot analysis. The probe used for hybridization in the upper panel is located in the central part of the *znuC1* coding sequence (Figure 5). Signals at about 3,000 nucleotides correspond in length to the complete *znu* operon mRNA, and signals at approximately 2,000 correspond in length to a bicistronic mRNA encompassing either *znu*A1 and *znu*C1 or *znu*C1 and *znu*B1. In the lower panel, the blot was hybridized with a probe against the 16S rRNA. A size marker is given in kilobytes at the left.

The transcript differences seen for the *znu* operon were validated using northern blot analysis probing for part of the *znu*C1 coding sequence (Figure 7). The *znu* transcript is more abundant in the strain without s479. In addition, results of the northern blot analysis confirm transcription of the *znu* operon as a single polycistronic transcript of about 3,000 nucleotides (Figure 7).

### Influence of s479 on Protein Abundance

Taking the analysis of the 479 target sphere a step further, we compared proteomes of the wild type and the s479 deletion strain. Since we saw differential expression of transporter genes in the transcriptome study, we used separate protocols for the maximum recovery of soluble as well as membrane-associated proteins for protein extraction, and samples were then analyzed separately by mass spectrometry (MS). In total, 22 proteins were exclusively present in the s479 deletion strain, whereas 18 proteins were not detected in Δ*s479* (Table 2 and Supplementary Table 2).

**TABLE 2:** Transporter components exclusively present or accumulated in the deletion strain.

**Gene**	**Product**	**On/log_2_**	**KEGG pathway/COG assignment**
HVO_B0198	ABC-type transport system periplasmic substrate-binding protein (probable substrate iron-III)	On	hvo02010—ABC transporters
HVO_B0047	ABC-type transport system periplasmic substrate-binding protein (probable substrate iron-III)	On	hvo02010—ABC transporters
HVO_2375	Putative phosphate ABC transporter periplasmic substrate-binding protein	On	hvo02010—ABC transporters
HVO_2397	**Putative zinc ABC transporter periplasmic substrate-binding protein**	**On**	hvo02010—ABC transporters
HVO_1705	Putative iron-III ABC transporter periplasmic substrate-binding protein	On	hvo02010—ABC transporters
HVO_2398	**Putative zinc ABC transporter ATP-binding protein**	**2.5**	hvo02010—ABC transporters
HVO_2324	Pantothenate permease	2.1	COG0591 code ER Na^+^/proline symporter

*Differential proteome analysis comparing the s479 deletion strain and wild-type strain was used determining the log_2_ fold change deletion vs. wild type (column on/log_2_). If detected only in the deletion strain, the protein is listed as “on.” Additionally, HVO–gene numbers (column gene) encoding the proteins are given alongside the gene product (column product) and KEGG/COG assignments (column KEGG pathway/COG assignment) informing on the metabolic pathway(s). Proteins encoded by the *znu* operon are given in bold.*

For 12 proteins, a significant differential abundance with log_2_ fold change ≥2 was measured. Eight of them accumulated and four were depleted in the deletion strain. A summary of the proteomic changes detected with a log_2_ fold change ≥0.8 is given in Supplementary Table 2. Comparison with the transcriptome data shows that the increase in transcript level seen for the *znu* operon is paralleled by an increase in protein level (*znu*C1, HVO_2398) or the exclusive detection of the gene product in the deletion strain (*znu*A1, HVO_2397). Analysis of the KEGG pathway assignment of the uniquely or differentially present proteins reveals an enrichment of transporter proteins exclusively present in the deletion strain (5 of 22) (Table 2 and Supplementary Table 2). However, the majority of proteins present in the deletion strain only are hypothetical proteins (9 of 22).

Since deletion of the s479 gene alters the expression of zinc transporters, we compared the growth of the wild-type and s479 strains in low zinc and high zinc concentrations. Under low zinc concentrations, wild type and Δ*s479* show the same growth behavior (data not shown). However, upon addition of high zinc concentrations, Δ*s479* shows defects in growth (Supplementary Figure 5).

### *In silico* Analysis of the sRNA–Target Interaction

**FIGURE 8:**
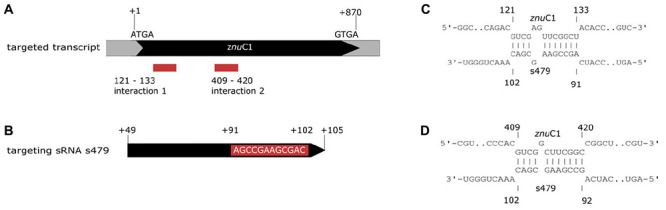
Interacting sequences of the *znu*C1 transcript and s479. **(A)** The two potential interaction sites for the *znu*C1 transcript are indicated by red boxes. The coding region is shown in black and numbering starts with the A of the start codon ATG. **(B)** The mature s479 is shown, and numbering is according to the full primary transcript as shown in Figure 3, where the mature s479 starts at +49. The part of s479 interacting with the *znu*C1 mRNA is shown in the red box, situated at the 3′ end. The predicted target–sRNA binding configuration is depicted in **(C)** for interaction 1 and in **(D)** for interaction 2.

As little is known about sRNA–target interactions in the archaeal domain and the few examples described so far reveal a diverse set of interaction modes, the potential interaction of s479 with the *znu*C1 coding region was further analyzed using bioinformatics. The sequence of the s479 (Figure 8B) was utilized to predict interactions with the *znu* operon transcript by the IntaRNA suite (Mann et al., 2017). We were able to predict two potential interaction sites, both within the *znu*C1 open reading frame, using standard settings (Figure 8A). Site 1 is located 120 nt downstream of the first nucleotide of the *znu*C1 coding sequence and site 2 is located 408 nt downstream of it with predicted energy gain of *E* = −7.82 for site 1 and *E* = −10.27 for site 2, respectively. The predicted interacting sequence of s479 is situated at the 3′ end.

**FIGURE 9:**
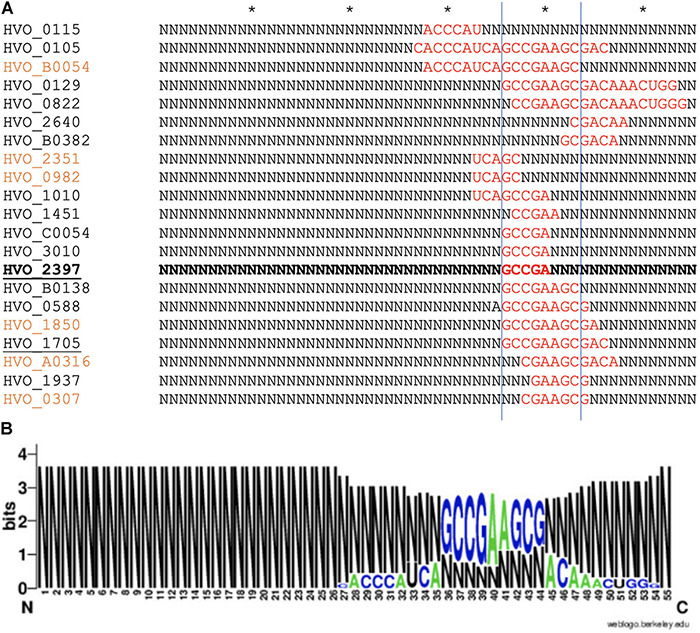
Predicted s479–target interaction sites for proteomic targets. **(A)** Results of the IntaRNA prediction of interacting sequences of s479 (Mann et al., 2017). The respective targeted mRNA is given at the left, and for the s479 sequence, all nucleotides not taking part in the predicted interaction (red) are depicted as N. The asterisk shown in the upper line marks 10 nucleotides. The proteomic targets included are those exclusively present or absent in Δ*s479* in comparison to H66. The *znu*A1 interaction site is given in bold, transporter targets are underlined, and targets predicted with seed length > 5 are given in orange. **(B)** The resulting conserved interaction sequence of s479 with its targets is shown as WebLogo at the right (Crooks, 2004).

As proteome analysis revealed additional potential targets of s479, the IntaRNA analysis was extended to the transcripts of these proteins as well. Since translational regulation commonly involves sequences at or in close proximity to the first codon, we incorporated the sequences from transcriptional start site to 50 nucleotides downstream^1^. Analysis was confined to proteins exclusively present or absent in the deletion strain, as these were the most affected. Using standard parameters, interaction with s479 was predicted for eight transcripts. We then modified the search parameters to also include seed sequences of only five nucleotides and relaxed specificity and this resulted in 21 predicted interactions. All of those interactions involve a similar region within s479 around position 44 to 54 at the 3′ end of the mature s479 (Figure 9). The WebLogo (Crooks, 2004) created for the s479 nucleotides involved in contacting all these transcripts highlights a nine-nucleotide consensus motif (GCCGAAGCG) corresponding to the interaction surface predicted for the s479–*znu*C1 contact.

### Verification of the s479–*znu*C1 Target Interaction

**FIGURE 10:**
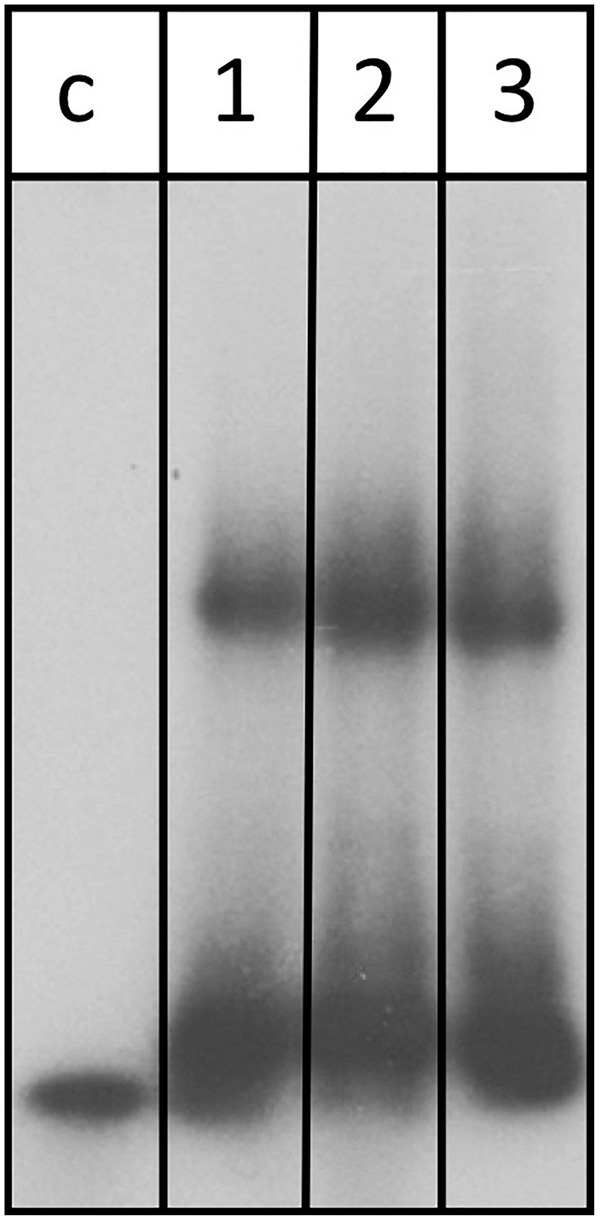
s479 binds to the *znu*C1 mRNA *in vitro*. Labeled s479 was incubated with increasing concentrations of a *znu*C1 mRNA fragment, containing the predicted interaction site 2 (Figure 7D). Reactions were loaded onto a nondenaturing polyacrylamide gel. Lane c: control reaction without the addition of *znu*C1, lane 1: addition of 50 pmol *znu*C1, lane 2: addition of 100 pmol *znu*C1, lane 3: addition of 200 pmol *znu*C1.

Assessment of the transcriptome by RNA-seq as well as northern blot analysis confirms that abundance of the *znu*C1 transcript is altered upon deletion of the s479 (Table 1 and Figure 7). Moreover, target site prediction suggests two interaction surfaces on the *znu*C1 mRNA (Figure 8). To validate whether the *znu*C1 transcript is a direct interaction partner of s479 and whether the predicted interaction site is correct, we performed an electrophoretic mobility shift assay (Figure 10).

Labeled s479 RNA was incubated with increasing amounts of an unlabeled *znu*C1 mRNA fragment, comprising the predicted interaction site 2 (Figure 8D) and flanking nucleotides (28 nt upstream, 40 nt downstream), and gel shift analysis shows that s479 binds to the *znu*C1 RNA (Figure 10). Competition experiments with unlabeled s479 were also performed (Supplementary Figure 3A) revealing that the unlabeled s479 competes effectively with the radioactively labeled one for binding to the *znu*C1 RNA. However, sRNA s479 does not bind to a *znu*C1 mutant RNA, which has the s479 interaction site (Figure 8D) deleted (Supplementary Figure 3B).

### s479 Is Bound by Cascade

The s479 precursor RNA contains almost a complete CRISPR repeat sequence (Supplementary Figure 2). Processing at the Cas6 cleavage site of this repeat sequence would yield the mature s479 RNAs containing a 5′ sequence identical to the characteristic eight nucleotide long 5′ handle of the mature *H. volcanii* crRNAs of locus P1 (5′ ATTGAAGC 3′) (Maier et al., 2013). This observation led us to investigate whether the Cas6 protein is involved in s479 biogenesis. Northern blot analysis shows that the short RNAs with about 50 nucleotides length are lost upon *cas6* deletion (Figure 4), and only an intermediate RNA of about 100 nucleotides is detected, suggesting that Cas6 indeed generates the mature sRNA.

We also analyzed whether s479 is present in a *cas7* deletion strain. An earlier investigation showed that Cas7 is the central subunit of the *Haloferax* Cas protein complex Cascade which binds the crRNAs. In a *cas7* deletion strain, the Cascade complex cannot form anymore; thus, crRNAs are not bound to Cascade and, therefore, are not stable anymore (Brendel et al., 2014). We used RNA from such a *cas7* deletion strain to monitor the s479 interplay with the Cascade complex. Indeed, in a Δ*cas7* strain, s479 is not detectable anymore, suggesting that s479 is bound and protected by Cascade as observed for crRNAs. Such an interaction with Cascade would be the first example for a non-crRNA bound by a type I-B Cascade used for internal gene expression regulation.

To determine whether the *znu* operon is upregulated in Δ*cas6* and Δ*cas7* strains, we probed a northern membrane containing RNA from these deletion strains with the *znu*C1 probe (Figure 6 and Supplementary Figure 4). Higher concentrations of *znu* mRNA are detected in the Δ*cas7* strain (Supplementary Figure 4), as expected when s479 is missing. Interestingly, in the Δ*cas6* strain, the *znu* RNA is not upregulated although the mature s479 is not present (Figure 4). Thus, the longer s479 intermediate with about 100 nucleotides length present in the Δ*cas6* strain seems to be also active in regulating the *znu* RNA.

## Discussion

Despite decades of research, archaeal sRNA networks are still enigmatic. It is well established that archaeal sRNAs play crucial roles in gene regulatory networks related to metabolism and, therefore, are essential players in stress responses, but pinpointing their interaction partners remained challenging (Buddeweg et al., 2018a). An inventory of small transcripts has been made for more than seven archaeal species, but sRNA–target pairs have been identified for only four of them (Buddeweg et al., 2018b; Gelsinger and DiRuggiero, 2018b; Orell et al., 2018). We report here data for the second sRNA–target RNA pair of *H. volcanii*.

### Deletion of the s479 Gene Has an Impact on Growth and the Transcriptome

*Haloferax volcanii* encodes two operons for putative zinc ABC transporters (*znu*A1-C1 and *znu*A2-C2 genes); without s479, a higher abundance of transcripts for one of the two operons is observed (*znu*A1-C1), which is confirmed by northern blot analysis. Moreover, proteome analysis comparing s479 deletion and wild type also identifies *znu*1 gene products as differentially regulated. In addition, our data support a direct interaction of s479 with the transcript of the zinc transporter gene *znu*C1. IntaRNA prediction reveals that the 3′ end of the s479 interacts with the coding region of *znu*C1. The energy gain predicted for the interaction implies a stable sRNA–target pairing (Kliemt et al., 2019). The few examples of archaeal sRNA–target pairs described to date suggest a nonuniversal mode of action and a wide variability in the site of target contact (Buddeweg et al., 2018b; Gomes-Filho et al., 2018). The binding site for s479 within the coding region of the targeted mRNA is a feature shared with the only other sRNA–target pair described for *H. volcanii* (sRNA s132) (Kliemt et al., 2019) and with examples from other species [*M. mazei* s154, *Sulfolobus acidocaldarius* RrrR(+)] (Prasse et al., 2017; Orell et al., 2018). EMSA analysis revealed s479 binding to the *znu*C1 RNA (Figure 10), confirming a direct interaction of both RNAs. This is the first *H. volcanii* sRNA–target pair for which the interaction has been confirmed by a gel shift experiment. We hereby also confirm that s479 is exerting direct control of the *znu*C1 transcript. This results in a negative effect on *znu*C1 abundance in the cellular context which can be released by s479 deletion as seen in northern blot analysis. Destabilizing the target by potentially binding within the coding sequence is also suggested for *S. acidocaldarius* RrrR(+) (Orell et al., 2018), but archaeal RNases have not been studied in great depth yet, and therefore, no candidate RNase is evident for direct degradation of dsRNA (no RNase III activity has been described in archaea) or for ssRNA cleavage upon dsRNA formation (Clouet-d’Orval et al., 2018).

The s479 deletion strain has a slight growth disadvantage compared to the wild type during late exponential growth. The growth rate of the deletion strain is retarded by 22% compared with wild type during late exponential phase resulting in a lower end point of growth as well, showing that the sRNA is required for normal growth. The fact that s479 only regulates one of the two *znu* operons might be the reason that deletion of the s479 gene has only a slight impact on growth. The second *znu* operon could be regulated by another sRNA or other factors and concerted regulation of both *znu* operons might require an as yet unknown master regulator.

### Deletion of the s479 Gene Has a Severe Impact on the Proteome

The differential proteome analysis revealed a much larger regulon for s479 on the protein level than seen on the transcript level suggesting that the primary effect of s479 is at the translational level. s479 is severely affecting the presence of more than 50 proteins. In contrast to the transcriptional level, where s479 acts as negative regulator, proteome analysis revealed proteins less abundant in the deletion strain, too. Such a duality in the direction of regulation achieved by a single sRNA has also been described for the other *H. volcanii* sRNA s132 (Kliemt et al., 2019). Also, like s132, s479 is implicated in both accumulation and depletion of certain proteins. Whether this effect is direct or indirect *via* intermediate gene products regulated by s479 must be analyzed in future experiments. However, it already demonstrates that sRNA-based regulation in *H. volcanii* is a complex and multimodal process in case of both sRNAs as they are addressing a multitude of targets.

### Involvement of s479 in a Transporter Regulation Network

The common theme reflected in the functions assigned to the proteins influenced by s479 reveals a network of transporters and transport-related proteins. Among the proteins exclusively present in the s479 deletion strain are six components of ABC transporters including proteins encoded by the *znu* operon, which is regulated by s479 on the transcriptional level. The transported substances besides zinc are phosphate and iron. All of them are influx transporters and suggest a role of s479 in regulating the cellular network of metal ion and phosphate transporters. Taking into consideration proteins, which are differentially expressed with log_2_ fold change 2, this is even more pronounced; 13 components of ABC transporters accumulated upon loss of s479 (Supplementary Table 2) including zinc, iron, phosphate, and peptide substrates. Therefore, a potential role for s479 might be in regulating metallostasis. A cross talk between the regulation of metal ion concentrations can be seen in bacteria, for instance for the regulatory networks of transcription factors Zur regulating zinc response and Fur regulating iron homeostasis in *Caulobacter crescentus* (Mazzon et al., 2014) or even within the transport itself, e.g., *Yersinia pestis* possesses a second Zn^2+^ transporter that engages components of the yersiniabactin (Ybt) siderophore-dependent transport system for iron (Bobrov et al., 2017). Further work is needed, to unravel, whether those translational effects are mediated directly or through secondary effects of other members of the regulon, e.g., the translation initiation factor aIF-5A. However, the direct effect of s479 is plausible for at least a subset of targets; as for 31 of the proteins on/off regulated in the s479 deletion strain, a site for physical interaction between the mRNA and the sRNA regulator could be predicted. Interestingly, the majority of interaction sites all map to the same part of the s479 sequence already predicted for the s479::*znu*C1 interaction (Figures 8, 9), which was confirmed as direct contact by gel shift analysis. However, as it is not yet resolved entirely how translation initiation ensues on the mostly leaderless transcripts of *H. volcanii* (Babski et al., 2016), future work is needed to unravel how translational control by small RNAs is exerted in this archaeal species. Interestingly, despite the large regulon of s479 especially on the protein level, deletion is not deleterious for the cell. Only under high zinc concentrations the deletion strain Δ*s479* shows substantial growth defects. This hints at an interdependent network of regulatory mechanisms that might involve other sRNAs or translational regulators balancing the cost of individual gene losses.

### The CRISPR–Cas Connection

**FIGURE 11:**
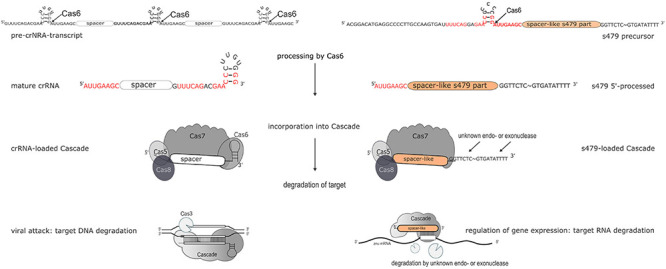
Maturation and function of crRNAs and s479. CRISPR arrays are transcribed into long precursors that are processed by Cas6 to yield the mature short crRNAs (left side). The mature crRNA is then loaded into the Cascade complex and guides Cascade to the invader to degrade the invader DNA. According to the data presented here, s479 is also transcribed into a precursor RNA and processed by Cas6. s479 seems to be bound by Cascade *via* the crRNA-like 5′ handle and s479 might guide Cascade to the target *znu*C1 mRNA triggering its degradation.

The s479 RNA is encoded between a CRISPR locus and the *cas* gene cluster. In addition, the s479 primary transcript contains a sequence highly similar to the CRISPR RNA repeat sequences (Supplementary Figure 2). CRISPR RNAs are processed by the endonuclease Cas6 at these repeat sequences to yield the functional crRNAs (Figure 11; Supplementary Figure 2; Maier et al., 2019). Northern experiments confirmed that cells without Cas6 cannot generate mature s479.

However, a 100-nucleotide RNA is still present and seems to be sufficient for regulating the *znu* mRNA. In addition, cells without Cas7 and thereby without Cascade contain neither the mature s479 nor the 100 nucleotide RNA (Figure 4), suggesting that s479 is bound and protected by Cascade. The *znu* RNA is clearly upregulated in a Δ*cas7* strain similar to the upregulation in the Δ*s479* strain. The fact that the mature s479 contains a typical crRNA 5′ handle and crRNAs are bound to Cascade *via* the conserved 5′ handle makes it even more likely that s479 is bound by Cascade. Such a dependence and interaction of s479 with Cas6 and Cascade, respectively, would be the first example for a non-crRNA bound by a type I-B Cascade used for internal gene expression regulation, pointing to a connection between the evolutionary origin of this sRNA as a drift from the CRISPR–Cas machinery, evolved to control gene expression at the mRNA level. A similar mode of gene regulation by Cas proteins has so far only been shown in detail for type II systems (Dugar et al., 2018; Ratner et al., 2019). In *Campylobacter jejunii* for instance, Cas9 is guided by a crRNA to mRNA targets, inducing RNA cleavage by Cas9 thereby regulating the expression of these genes (Dugar et al., 2018).

Further work will show whether s479 guides Cascade to the *znu*C1 mRNA and triggers degradation of the mRNA (Figure 11).

## Conclusion

s479 supports a role for sRNAs as substantial regulatory players within the metabolic networks of *H. volcanii* especially in regulating metabolite availability as s479 seems to harmonize the abundance of several influx transporters of the ABC-type regulating the zinc, iron, peptide, and phosphate flux of the cell. We demonstrate the direct interaction of s479 to its target *znu*C1 mRNA. Furthermore, our data show that s479 is linked to the CRISPR–Cas system and might act together with Cascade to regulate zinc transport proteins.

## Materials and Methods

### Strains and Growth Conditions

Strains and oligonucleotides used in this study are listed in Supplementary Tables 3, 4. A detailed description of the media used can be found in the Supplementary Data. Cloning procedures were performed using *E. coli* strain DH5α and standard culture (aerobically, 37°C, 2YT media) as well as molecular biological techniques (Sambrook, 2006). *H. volcanii* strains were grown aerobically at 45°C in either YPC, Hv-Ca, or Hv-MM with appropriate supplements (Allers et al., 2004; Duggin et al., 2015; de Silva et al., 2020). For in-depth comparison of growth, transcriptome, and proteome, H66 (Δ*pyr*E2, Δ*leu*B) was used as wild type, since in the s479 deletion strain, the s479 gene is replaced by a tryptophan marker in the genome of the parent strain H119 (Δ*pyr*E2, Δ*trp*A, and Δ*leu*B). Thus, Δ*s479* and H66 require both the addition of uracil and leucine to media for growth (Allers et al., 2004; Jaschinski et al., 2014).

### Growth Experiments

Growth experiments were carried out in microtiter plates using a heated plate reader (Epoch 2 NS Microplate Spectrophotometer, BioTek Instruments). Strains H66 (wild type) and Δ*s479* were precultured in Hv-Ca medium supplemented with uracil to OD_650 nm_ = 0.4–0.7 and then diluted to OD_650 nm_ = 0.05 and transferred to microtiter plates in triplicates. These were then cultured (aerobically, orbital shaking, 45°C) while OD_650 nm_ was measured every 30 min. Outer wells were filled with salt water as evaporation barriers (Jaschinski et al., 2014). For stress conditions, adjusted media preparations were used (see section *“*Strains and Growth Conditions*”*). Doubling time [*d* (h)] and growth rate [*μ* (h^–1^)] were calculated as growth rate *μ* = (ln(*x*_*t*_) − ln (*x*_0_)) / (*t* − *t*_0_) and doubling time *d* = ln(2) / *μ*. Calculations were carried out separately for all replicates before calculating mean value and standard deviation. Phases of exponential growth were identified using fitted trendlines and corresponding *R*^2^ values (Supplementary Figures 5B,C).

### Northern Blot Analysis

For the analysis of s479 transcripts (Figure 2), *Haloferax* strains H119 and Δ*s479* were cultivated in Hv-MM supplemented with leucin and uracil (for H119, tryptophan was also added); for the detection of *znu* mRNAs (Figure 7), *Haloferax* strains H66 and Δ*s479* were grown in YPC. For the detection of s479 transcripts in Figure 4, the *Haloferax* strain H119 was cultivated in Hv-MM supplemented with leucin, tryptophan, and uracil; deletion strains Δ*cas6* and Δ*cas7* were cultivated in YPC. For the investigation of *znu* mRNAs in Cas protein deletion strains Δ*cas6* and Δ*cas7* (Supplementary Figure 4) H119, Δ*cas6* and Δ*cas7* were grown in YPC. TRIzol reagent (Invitrogen, Thermo Fisher Scientific) or NucleoZOL^TM^ (Machery and Nagel) was used to isolate total RNA from *H. volcanii* cells. Ten micrograms of total RNA was separated using a 1.5% agarose (transcript size > 500 nt) or 8 % denaturing polyacrylamide gel (PAGE) and then transferred to a nylon membrane (Biodyne^®^ A, PALL or Hybond-N+, GE Healthcare). After transfer, the membrane of PAGE blots was hybridized with oligonucleotide s479spacerpart (primer sequences are listed in Supplementary Table 4) to detect the s479 transcript, and the membrane was subsequently hybridized with an oligonucleotide against the 5S rRNA, both radioactively labeled with [γ-^32^P]-ATP via polynucleotide kinase treatment. Membranes of agarose blots were hybridized with a probe against *znu*C1 generated by PCR using primers “probe znuC1 Hvo_2398 fw/rev” and genomic DNA as template, and the product was labeled using [α-^32^P]-dCTP and the random primed DNA labeling kit DECAprime^TM^II (Invitrogen). In addition, the membrane was hybridized with a probe against the 16S rRNA. The probe was generated with PCR using primers 16Sseqf and 16Sseqrev and genomic DNA from *H. volcanii* as template. Using the DECAprimeTMII kit (Invitrogen), the PCR fragment was radioactively labeled with [α-^32^P]-dCTP. For oligonucleotide probes and PCR primers, see Supplementary Table 4.

### Sample Preparation for Transcriptome Analysis and RNA-Seq Analysis

Three replicates of wild type (H66) and deletion strain (Δ*s479*) were cultured in Hv-Ca medium supplemented with uracil at 45°C and grown to OD_650 *nm*_ = 0.6–0.7. Total RNA was isolated using NucleoZOL^TM^ (Machery and Nagel), and RNA samples were sent to Vertis Biotechnologie AG (Martinsried, Germany) for further treatment. Total RNA was treated with T4 Polynucleotide Kinase (NEB) and rRNA depleted using an in-house protocol, and cDNA library preparation was preceded by ultrasonic fragmentation. After 3′ adapter ligation, first-strand cDNA synthesis was performed using 3′ adapter primer and M-MLV reverse transcriptase. After cDNA purification, the 5′ Illumina TruSeq sequencing adapter was ligated to the 3′ end of the antisense cDNA and the sample amplified to 10–20 ng/μl using a high-fidelity DNA polymerase. Finally, cDNA was purified using the Agencourt AMPure XP kit (Beckman Coulter Genomics), samples were pooled (equimolar), and the pool size fractionated (200–550 bp) by preparative agarose gel electrophoresis and sequenced on an Illumina NextSeq 500 system using 1 × 75 bp read length. TruSeq barcode sequences which are part of the 5′ TruSeq sequencing adapter are included in Supplementary Table 4. Sequencing reads are deposited at the European Nucleotide Archive (ENA) under the study accession number PRJEB41379. For data analysis, reads were mapped to the genome using bowtie2 (version 2.3.4.1) with the “–very-sensitive” option and defaults otherwise (Langmead et al., 2009). Then, reads per feature were counted using featureCounts (version 1.6.4) and analyzed for differential expression with DeSeq2 (version 1.2.11) (Liao et al., 2014; Love et al., 2014).

### Sample Preparation for MS/MS Analysis

Three biological replicates (250 ml) of the wild type (H66) and deletion strain (Δ*s479*) were cultivated in Hv-Ca media supplemented with uracil, 45°C, and grown to OD_650 *nm*_ = 0.6–0.74. Cells were harvested and washed in enriched PBS buffer (2.5 M NaCl, 150 mM MgCl_2_, 1 × PBS, 137 mM NaCl, 2.7 mM KCl, 8 mM Na_2_HPO_4_, 2 mM K_2_HPO_4_, pH 7.4). After cell lysis by ultrasonication in 10 ml lysis buffer [1 M NaCl, 100 mM Tris–HCl, pH 7.5, 1 mM EDTA, 10 mM MgCl_2_, 1 mM CaCl_2_, 13 μl/ml protease inhibitor mixture (Sigma)], cytosolic and membrane fractions were separated by ultracentrifugation at 100,000 × *g* and treated as separate samples. The cytosolic protein sample was directly used for 1D SDS-PAGE, whereas the pelleted membrane protein fraction was solubilized in 2 ml HTH buffer [(6 M thiourea/2 M urea); 10 min 37°C; 10 min 37°C ultrasonication]. Twenty micrograms of both samples were separated by 1D SDS-PAGE and in-gel digested as previously described (Bonn et al., 2014). Briefly, Coomassie-stained gel lanes were cut resulting in 10 gel pieces per sample before gel pieces were cut into smaller blocks and transferred into low binding tubes. Samples were destained and dried in a vacuum centrifuge before being covered with trypsin solution. Digestion was carried out at 37°C overnight before peptides were eluted in water by ultrasonication. The peptide-containing supernatant was transferred into a fresh tube and desiccated in a vacuum centrifuge, and peptides were resolubilized in 0.1% (v/v) acetic acid for mass spectrometric analysis.

### MS/MS Analysis

LC-MS/MS analyses were performed on an LTQ Orbitrap Velos Pro (Thermo Fisher Scientific, Waltham, Massachusetts, USA) using an EASY-nLC II liquid chromatography system. Tryptic peptides were subjected to liquid chromatography (LC) separation and electrospray ionization-based mass spectrometry (MS) applying the same injected volumes in order to allow for label-free relative protein quantification. Therefore, peptides were loaded on a self-packed analytical column (OD 360 μm, ID 100 μm, length 20 cm) filled with 3 μm diameter C18 particles (Dr. Maisch, Ammerbuch-Entringen, Germany) and eluted by a binary nonlinear gradient of 5–99% acetonitrile in 0.1% acetic acid over 86 min with a flow rate of 300 nl/min. For MS analysis, a full scan in the Orbitrap with a resolution of 30,000 was followed by collision-induced dissociation (CID) of the 20 most abundant precursor ions. MS2 experiments were acquired in the linear ion trap.

### MS Data Analysis

Database search was performed with MaxQuant 1.6.17.0 against a *H. volcanii* database (Jevtić et al., 2019) containing 4,106 entries. MaxQuant’s generic contamination list as well as reverse entries was added during the search. The following parameters were applied: digestion mode, trypsin/P with up to two missed cleavages; variable modification, methionine oxidation and N-terminal acetylation, and maximal number of five modifications per peptide; activated LFQ option with minimal ratio count of two and “match-between runs” feature. The false discovery rates of the peptide spectrum match and the protein level were set to 0.01. A protein was considered to be identified if two or more unique peptides were identified in a biological replicate. Only unique peptides were used for protein quantification.

The comparative proteome analyses based on MaxQuant LFQ values were performed separately for cytosolic and membrane protein samples. Proteins were considered to be quantified if a quantitative value based on at least two unique peptides was available in at least two biological replicates. LFQ values as proxy for protein abundance were used for statistical analysis. Student’s *t* test was performed to analyze changes in protein amounts between wild type and mutant. Proteins with significantly changed amount exhibited a *p* value < 0.01 and an average log_2_ fold change > |0.8|.

### Electrophoretic Mobility Shift Assay

For electrophoretic mobility shift assay (EMSA), RNAs were obtained from Biomers (Ulm, Germany) (sequences are listed in Supplementary Table 4). The s479 RNA was labeled at the 3′ end using [α-^32^P]-pCp and T4 RNA ligase (Fermentas, Thermo Fisher Scientific). For the EMSA in Figure 10, 100 cps labeled s479-RNA was mixed with 50, 100, or 200 pmol of the unlabeled *znu*C1 RNA fragment encompassing interaction site 2 (Figure 8D). For the EMSA in Supplementary Figure 3A, 100-cps labeled s479 RNA was mixed with 50 pmol unlabeled *znu*C1 RNA encompassing interaction site 2 (Figure 8D); in addition, 0, 50, 200, or 400 pmol of the unlabeled s479 was added. For the EMSA in Supplementary Figure 3B, 100-cps labeled s479 RNA was mixed with 50 pmol unlabeled *znu*C1 RNA encompassing interaction site 2 (Figure 8D) or 50, 200, or 400 pmol of unlabeled *znu*C1 RNA mutant, which has the s479 interaction site deleted (Figure 8D). All reactions were performed in 20 μl reaction volume containing 10 mM Tris–HCl pH 7.5, 5 mM MgCl_2_, and 100 mM KCl. After incubation at 37°C for 30 min, 1 μl 50% (vol/vol) glycerol containing 0.1% (w/vol) bromphenol blue was added, and the samples were separated on a native 8% (w/vol) polyacrylamide gel at 4°C which was subsequently analyzed by autoradiography.

### *In silico* Target Site Prediction

To predict the target sites of s479 *in silico*, we applied IntaRNA (version 3.2.0) (Mann et al., 2017). For the prediction of potential s479 interaction sites, we used the s479 sequence corresponding to pHV4: 207,716–207,770. This corresponds to the start point of the potential spacer sequence of the shortened s479 versions (Figure 3). The spacer is the sequence located downstream of the 5′ handle sequence within crRNAs. In crRNAs, the spacer sequence is the sequence used for target recognition. Therefore, we chose this part of the sequence for analysis and set the spacer length to 55 nt. IntaRNA was used with default settings for the prediction of the s479::*znu*C1 interaction sites. For the prediction of the interaction sites on the proteome–targets, we first used default settings and then also included predictions for a seed sequence of five nucleotides as this is the increment of protein contacts seen for spacer sequences within Cascade complexes (Maier et al., 2019).

## Data Availability Statement

The transcriptome data were deposited in the European Nucleotide Archive (ENA) at EMBL-EBI under study accession number: PRJEB41379. Proteome data was deposited to the ProteomeXchange Consortium via the PRIDE partner repository (Vizcaíno et al., 2016) with the dataset identifier PXD022750.

## Author Contributions

PM, L-KM, SM, CH, and JB did the experiments. BV carried out the bioinformatics analyses. PM, L-KM, AM, SM, DB, and BV performed data curation. AM conceptualized the project. L-KM, PM, and AM wrote the original draft. SM, JB, DB, BV, L-KM, and AM reviewed and edited the draft. DB, BV, and AM provided the resources and funding. All authors contributed to the article and approved the submitted version.

## Conflict of Interest

The authors declare that the research was conducted in the absence of any commercial or financial relationships that could be construed as a potential conflict of interest.

## Footnotes

1 For targets without a predicted TSS (Babski et al., 2016) or targets located in operons, we included 100 nt upstream of the first coding nucleotide.
