# Supplementary material for: A Small RNA Is Linking CRISPR–Cas and Zinc Transport
Source: Front Mol Biosci. 2021 May 13;8:640440. doi: 10.3389/fmolb.2021.640440 (PMC8155600; doi:10.3389/fmolb.2021.640440)
Supplement: Supplementary file 1 [file Data_Sheet_1.pdf]

Supplementary data

Supplementary Figures	page 2
Supplementary Methods and Materials	page 8
Supplementary Tables	page 9

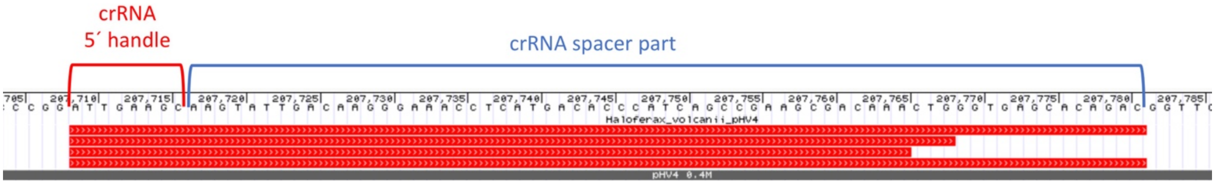

Supplementary Figure 1. RNA-seq analysis of small RNAs reveals shortened s479 variants. Sequencing data from an RNA-seq study dedicated to identification of small RNAs revealed reads mapping to the s479 locus all starting at position pHV4: 207,708. The reads extend to s479 length variants of 57, 60 and 73 nt. The sequence that is identical to the crRNA 5´ handle is indicated with a red bracket; this is the part that interacts with the Cas5 protein in the Cas protein complex Cascade. The sequence downstream of the 5´ handle corresponds to the crRNA spacer sequence and is indicated with a blue bracket.

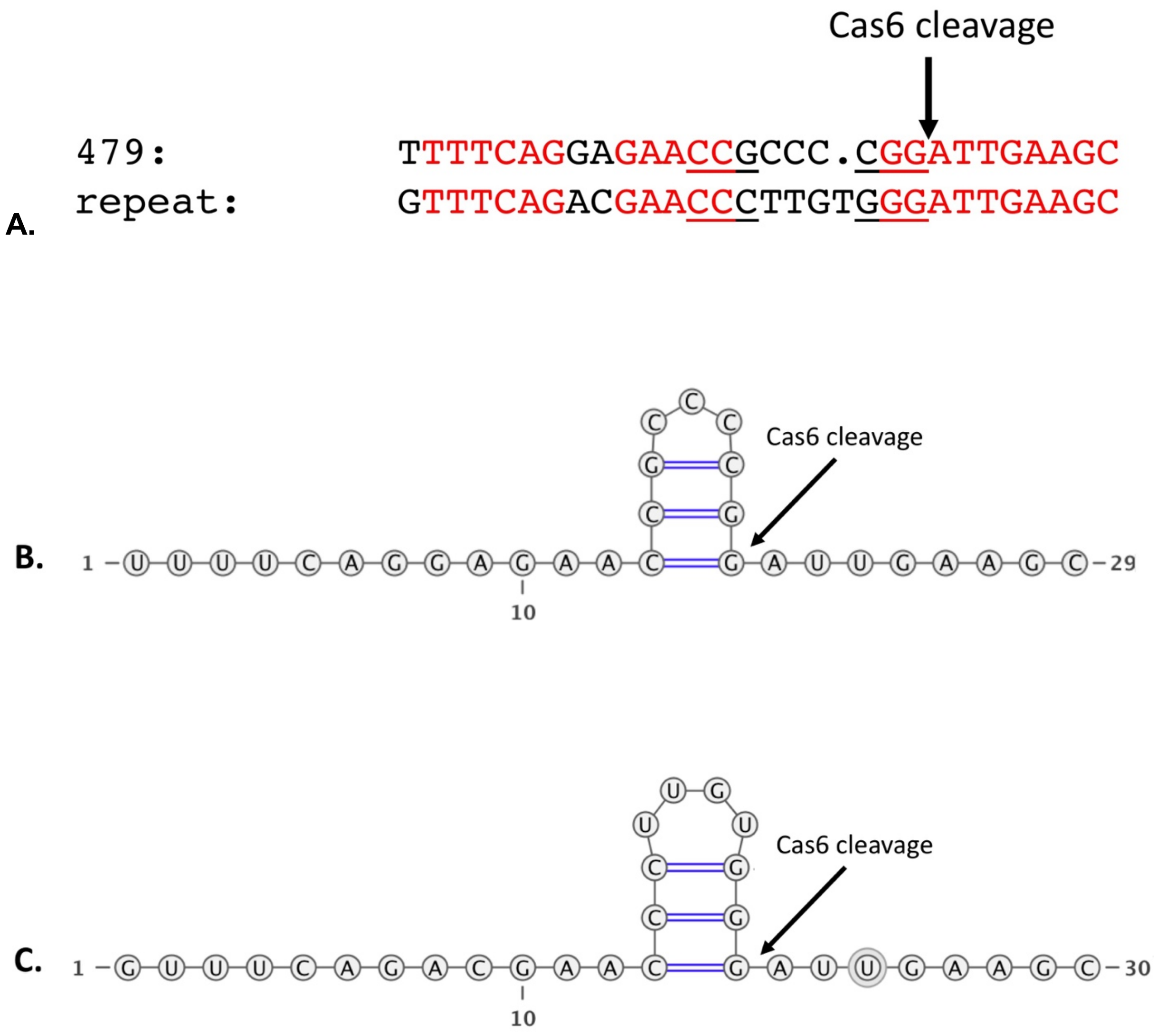

Supplementary Figure 2. Similarity of the s479 precursor and crRNA precursors (pre-crRNA). A. The s479 coding sequence of the precursor is highly similar (red nucleotides) to the repeat sequence of the CRISPR locus P1 flanking it upstream. The s479 precursor repeat-like sequence shows the potential to fold into a stem-loop structure (flanking nucleotides underlined). The Cas6 processing site is indicated by an arrow. **B.** The resulting stem-loop of the s479 precursor lies just upstream of the future handle sequence (AUUGAAGC) as seen for *H. volcanii* crRNAs (Maier et al., 2019). If the s479 precursor is processed by Cas6 like the CRISPR pre-crRNA, an endonucleolytic cut at the base of this stem-loop (shown by an arrow) yields the 5´-handle as seen in mature crRNAs of locus P1 and as seen in RNA-Seq. **C.** The structure of the CRISPR crRNA is shown for comparison.

A.

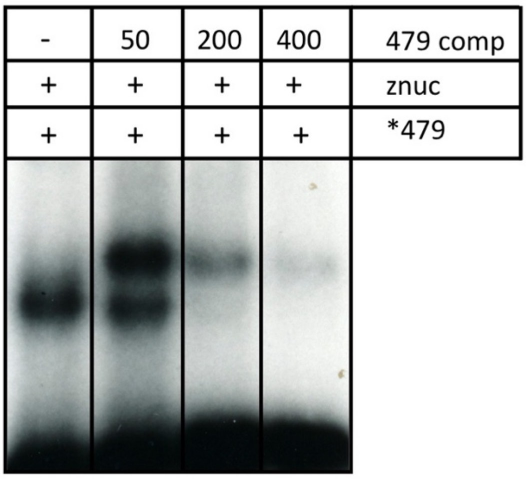

B.

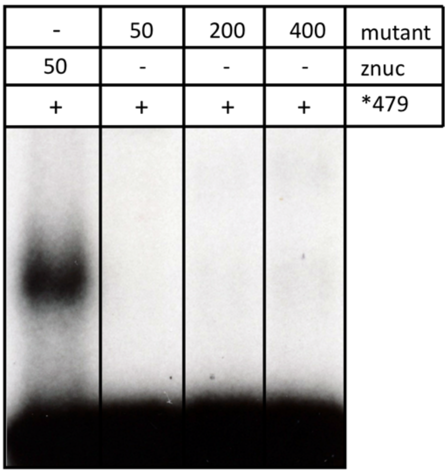

Supplementary Figure 3. Gel shift experiments with s479. Labelled s479 was incubated with different RNAs. Reactions were loaded onto a non-denaturing polyacrylamide gel to separate the molecules.

A. Addition of unlabelled sRNA s479 competes with binding of radioactively labelled sRNA s479 to *znu*C1 RNA. Radioactively labelled s479 (*479) was incubated with the *znu*C1 mRNA fragment (as shown in Figure 9.). Increasing amounts of unlabelled s479 were added (lanes 479 comp: 50, 200 and 400 pmol). The unlabelled s479 clearly competes with binding of the labelled s479. Addition of unlabelled s479 results in an additional bigger complex, which runs slower in the gel. This might be due to binding of more than one s479 molecules to the *znu*C1 mRNA.

B. s479 does not bind to a mutant *znu*C1 RNA without the binding site. Radioactively labelled s479 (*479) was incubated with a *znu*C1 RNA mutant, which has the s479 interaction site deleted. s479 does not bind to the mutant *znu*C1 RNA. Different amounts of the *znu*C1 mutant RNA were incubated with the radioactively labelled s479 (lanes mutant: 50 pmol, 200 pmol and 400 pmol). As positive control the radioactively labelled s479 was incubated with 50 pmol wild type *znu*C1 RNA (as shown in Figure 9) (lane znuc).

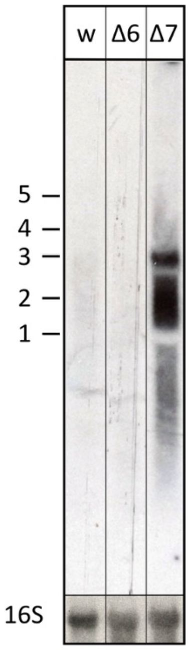

Supplementary Figure 4. Transcript levels of the *znu* operon in wild type and Cas protein deletion strains. We compared the transcript levels of *znu* in total RNA of wild type H119 (lane w), Δ*cas6* (lane Δ6) as well as Δ*cas7* cells (lane Δ7) using northern blot analysis. The probe used for hybridisation in the upper panel is located in the central part of the *znuC1* coding sequence (Figure 5). Signals at about 3,000 nucleotides correspond in length to the complete *znu* operon mRNA, signals at approximately 2,000 correspond in length to a bicistronic mRNA encompassing either *znu*A1 and *znu*C1 or *znu*C1 and *znu*B1. In the lower panel the blot was hybridised with a probe against the 16S rRNA. A size marker is given in kb at the left.

The wild type strain has almost no detectable *znu* mRNA, while high concentrations of the *znu* mRNA are visible in the Δ*s479* strain (Figure 6). Similar high concentrations are found in the Δ*cas7* strain confirming that Cas7 is not only important for a stable s479 as shown in Figure 10 but also for regulation of the *znu* mRNA. Interestingly, although Δ*cas6* is important for generating the mature s479 (Figure 10), the 100 nucleotide s479 intermediate seems sufficient for the regulation of the *znu* mRNA, since *znu* mRNA is not detected in a Δ*cas6* strain.

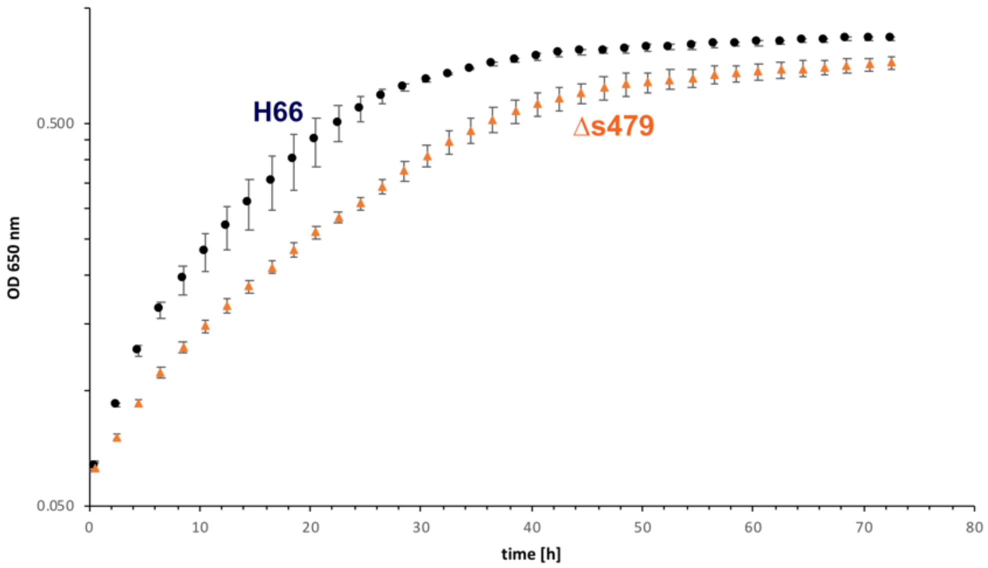
H66
Δs479
0.500
0.050
OD 650 nm
0 10 20 30 40 50 60 70 80
time [h]

A.

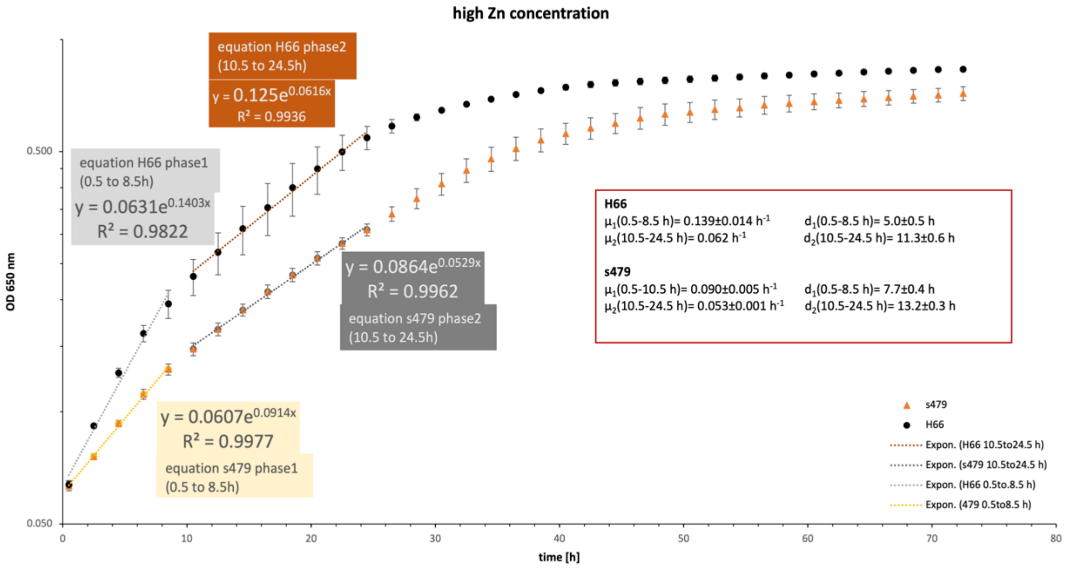
high Zn concentration
equation H66 phase2
(10.5 to 24.5h)
y = 0.125e^0.0616x
R² = 0.9936
equation H66 phase1
(0.5 to 8.5h)
y = 0.0631e^0.1403x
R² = 0.9822
y = 0.0864e^0.0529x
R² = 0.9962
equation s479 phase2
(10.5 to 24.5h)
y = 0.0607e^0.0914x
R² = 0.9977
equation s479 phase1
(0.5 to 8.5h)
H66
μ1(0.5-8.5 h)= 0.139±0.014 h-1
μ2(10.5-24.5 h)= 0.062 h-1
d1(0.5-8.5 h)= 5.0±0.5 h
d2(10.5-24.5 h)= 11.3±0.6 h
s479
μ1(0.5-10.5 h)= 0.090±0.005 h-1
μ2(10.5-24.5 h)= 0.053±0.001 h-1
d1(0.5-8.5 h)= 7.7±0.4 h
d2(10.5-24.5 h)= 13.2±0.3 h
s479
H66
Expon. (H66 10.5to24.5 h)
Expon. (s479 10.5to24.5 h)
Expon. (H66 0.5to.8.5 h)
Expon. (479 0.5to8.5 h)
0.500
0.050
OD 650 nm
0 10 20 30 40 50 60 70 80
time [h]

B.

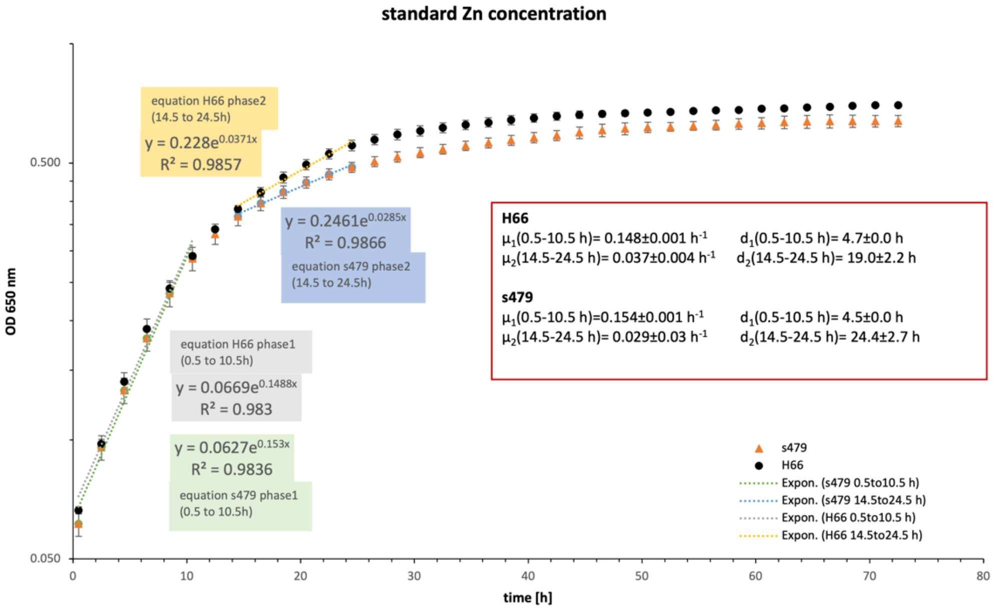

C.

Supplementary Figure 5. Growth of wild type and Δ*s479* strains in different media.

A. Growth in medium with high zinc concentrations. The deletion mutant has a delayed growth compared to the wild type strain. Growth curves show a diauxic growth behaviour, doubling times for phase 1 (0.5 h- 8.5 h) are 5 hours for wild type cells and 7.7 hours for the deletion strain. In phase 2 (10.5 h - 24.5 h) the doubling time for the wild type strain is 11.3 and for the deletion strain 13.2.

B. Determination of doubling times and growth rates. Slope analysis of the growth curves reveals a biphasic exponential phase. The trendline, line equation and R2 are given for phase 1 (0.5 h to 8.5 h) and phase 2 (10.5 h to 24.5 h) separately for each growth curve. The red box gives the calculated growth rate (μ) and doubling time (d) for each phase and strain as mean of the three replicates together with the respective standard deviation. Raw data and calculation are listed in Supplementary Table 6.

C. Determination of doubling times and growth rates for growth in standard medium (growth curve shown in Figure 4).

Slope analysis of the growth curves reveals a biphasic exponential phase. The trendline, line equation and R2 are given for phase 1 (0.5 h to 10.5 h) and phase 2 (14.5 h to 24.5 h) separately for each growth curve. The red box gives the calculated growth rate (μ) and doubling time (d) for each phase and strain as mean of the three replicates together with the respective standard deviation. Raw data and calculation are listed in Supplementary Table 5.

Supplementary Material and Methods

Media composition

Hv-YPC, Hv-Ca, and Hv-MM medium were used to grow *H. volcanii* (Allers *et al.*, 2004). Due to the sensitivity of *H. volcanii* to different ingredients Bacto yeast extract, casamino acids and peptone were used only from Oxoid. Unless otherwise stated, all components were autoclaved at 121 °C for 20 minutes.

YPC. For one liter of YPC liquid medium 600 ml 30% salt water (SW) (4.1 M NaCl, 148 mM $MgCl_2$, 142 mM $MgSO_4$, 94 mM KCl, 20 mM Tris/HCl pH 7.5), 300 ml double-distilled water and 99 ml 10x YPC stock solution (0.52% (w/v) Bacto yeast extract (Oxoid), 0.1% (w/v) peptone (Oxoid), 0.1% (w/v) casamino acids (Oxoid), adjust pH to 7.5 with KOH) were mixed and autoclaved. After cooling and before use 3 ml 1 M $CaCl_2$ (3 mM) was added.

Hv-Ca. For one liter of Hv-Ca medium, 600 ml SW, 225 ml double-distilled water and 30 ml 1M Tris-HCl/pH 7.5 were mixed and autoclaved. After cooling, 99 ml sterile-filtered 10x casamino acids (6% (w/v) casamino acids, 29.5 mM KOH.), 25.5 ml Hv-MM carbon source (10% sodium-DL-lactate, 9% succinic acid, 1% glycerol, adjust pH to 7.5 with NaOH), 12 ml Hv-MM salts (30 ml 1M NH_4Cl, 18 ml 1M $CaCl_2$, 6 ml trace elements, 18 ml double-distilled water); (trace elements: 0.0036 mg/ml $MnCl_2x4H_2O$, 0.0044 mg/ml $ZnSO_4x7H_2O$, 0.023 mg/ml $FeSO_4x7H_2O$, 0.0005 mg/ml $CuSO_4x5H_2O$), 1.95 ml 0.5 M KPO_4-buffer (83.4 ml 1 M K_2HPO_4, 16.6 ml 1 M KH_2PO_4, 100 ml double-distilled water) and 900 µl vitamin solution (1 mg/ml thiamine and 0.1 mg/ml biotin) were added to the medium. In addition, 1.02 ml (50 µg/ml) of sterile filtered uracil was added to the medium before use. To increase the zinc concentration for high-zinc medium, double the amount of $ZnSO_4$ was added in the trace elements. Please note that Hv-Ca medium contains a source of leucin.

Hv-MM. For one liter of Hv-MM medium, 600 ml of SW and 325 ml double-distilled water and 30 ml 1M Tris-HCl/pH 7.5 were autoclaved. After autoclaving, the following sterile filtered components were added, 25.5 ml Hv-MM carbon source (10% sodium-DL-lactate, 9% succinic acid, 1% glycerol, adjust pH to 7.5 with NaOH), 12 ml Hv-MM salts (30 ml 1 M NH_4Cl, 18 ml 1 M $CaCl_2$, 6 ml trace elements, 18 ml double-distilled water); (trace elements: 0.0036 mg/ml $MnCl_2x4H_2O$, 0.0044 mg/ml $ZnSO_4x7H_2O$, 0.023 mg/ml $FeSO_4x7H_2O$, 0.5 µg/ml $CuSO_4x5H_2O$), 1.95 ml 0.5 M KPO_4-buffer (83.4 ml 1 M K_2HPO_4, 16.6 ml 1 M KH_2PO_4, 100 ml double-distilled water) and 900 µl vitamin solution (9.6 ml thiamine 1 mg/ml, 1.2 ml biotin 1 mg/ml,) were added to the medium. In addition, if required 1.02 ml (50 µg/ml) of sterile filtered uracil, 5.1 ml (50 µg/ml) of sterile filtered tryptophan and 5.1 ml (50 µg/ml) of sterile filtered leucine was added to the medium before use.

Supplementary Table 1. Transcripts that are up- and down-regulated in the Δ*s479* strain. The log_2 fold change (column log_2) deletion vs. wild type is given alongside the HVO- gene number (column gene), p-value (column p.adj) and gene product name (column product). Green and red: up- and down-regulation, respectively.

gene	product	log_2	p.adj
s479	sRNA s479	-4.30	2.70E-31
HVO_B0158	hypothetical protein	-1.36	0.0439214
HVO_0943	ba3-type terminal oxidase subunit CbaD	-1.30	0.0083068
HVO_0930	hypothetical protein	-1.29	0.0351432
HVO_B0371	aldehyde dehydrogenase, AldH1	1.18	0.0227347
HVO_2395	ATP:cob(I)alamin adenosyltransferase, PduO	1.29	0.0439214
HVO_B0248	short-chain family oxidoreductase	1.47	0.0439214
HVO_B0071	alcohol dehydrogenase, Adh2	1.53	0.0164927
HVO_2469	SNF family transport protein	1.54	0.0017482
HVO_2397	ABC-type transport system periplasmic substrate-binding protein (probable substrate zinc), ZnuA1	1.81	5.62E-05
HVO_2399	ABC-type transport system permease protein (probable substrate zinc), ZnuB1	1.98	3.15E-07
HVO_2400	small CPxCG-related zinc finger protein	2.08	1.73E-08
HVO_2398	ABC-type transport system ATP-binding protein (probable substrate zinc), ZnuC1	2.11	1.58E-07
HVO_2396	glutaredoxin, Grx4	2.16	6.51E-07
HVO_2401	glycine cleavage system protein P beta subunit, GcvP2	3.27	1.66E-19

Supplementary Table 2. Proteins that accumulated or were depleted in the Δ*s479* strain. Differential proteome analysis comparing the s479 deletion strain and wild type H66 was used determining the $\log_2$ fold change (column on/off/$\log_2$). If detected only in the deletion strain the protein is listed as "on", if detected only in the wild type strain the protein is listed as "off". Additionally, HVO-gene numbers (column gene) encoding the proteins are given alongside the gene product (column gene product) and Kegg/COG assignments (columns Kegg pathway/ COG) informing on the metabolic pathway(s). The MS sample the protein was measured in (column S; c: cytoplasmic, m: membrane) and the predicted localization (column local; C: cytoplasm, M: membrane) are also included. Orange: off or depleted in Δ*s479* samples. Green: on or enriched in Δ*s479* samples. Italic: association with tryptophan metabolism.

S	gene product	gene	loc	cn/ off/ $\log_2$ (Δs479 / WT)	Kegg pathway	COG
c	A-type ATP synthase subunit E	HVO_0313	C	off	hvo00190 - Oxidative phosphorylation; hvo00680 - Methane metabolism;	
c	acid phosphatase	HVO_1314	C	off	hvo00230 - Purine metabolism; hvo00240 - Pyrimidine metabolism; hvo00760 - Nicotinate and nicotinamide metabolism; hvo01110 - Biosynthesis of secondary metabolites;	
c	glutamate dehydrogenase	HVO_1451	C	off	hvo00250 - Alanine, aspartate and glutamate metabolism; hvo00330 - Arginine and proline metabolism; hvo00910 - Nitrogen metabolism;	
c	*N-(5phosphoribosyl)anthranilate isomerase*	*HVO_2455*	*C*	*off*	*hvo00400 - Phenylalanine, tyrosine and tryptophan biosynthesis /hvo01110 - Biosynthesis of secondary metabolites*	
c	5,10-methylenetetrahydromethanopterin reductase	HVO_1937	C	off	hvo00680 - Methane metabolism; hvo01120 - Microbial metabolism in diverse environments;	
m	cobalamin 5-phosphate synthase	HVO_0588	C/M	off	hvo00860 - Porphyrin and chlorophyll metabolism	
c	50S ribosomal protein L39.eR	HVO_0115	C	off	hvo03010 - Ribosome;	
c	endonuclease IV	HVO_0573	C	off	vo03410 - Base excision repair	
c	acyl-CoA synthetase	HVO_A0551	C	off		COG0318 Code IQ Acyl-CoA synthetases (AMP-forming)/AMP-acid ligases II
c	cytochrome P450	HVO_1558	C	off		OG2124 Code Q Cytochrome P450
c	deoxyhypusine synthase	HVO_2297	C	off		OG1899 Code O Deoxyhypusine synthase
c	DnaJ domain-containing protein	HVO_1040	C	off		COG0484 Code O DnaJ-class molecular chaperone with C-terminal Zn finger domain
c	ExsB protein	HVO_1716	Unknown	off		COG0603 Code R Predicted PP-loop superfamily ATPase
c	hypothetical protein	HVO_2024	C	off		
c	hypothetical protein	HVO_2640	C	off		

c	colbalt chelase thioredoxin	HVO_B0054	C	off		COG2138 Code S Uncharacterized conserved protein
c	PQQ repeat protein	HVO_B0138	C	off		COG1520 Code S FOG: WD40-like repeat
c	Xaa-Pro aminopeptidase, M24 family protein	HVO_0414	Unknown	off		COG0006 Code E Xaa-Pro aminopeptidase
c	3-dehydroquinate synthase	HVO_0822	C	on		COG0371 Code C Glycerol dehydrogenase and related enzymes
c	NADH dehydrogenase-like complex subunit I	HVO_0982	C	on	hvo00190 - Oxidative phosphorylation;	
m	glycerol-3-phosphate dehydrogenase subunit B	HVO_1539	C/M	on	hvo00564 - Glycerophospholipid metabolism	
m	ABC-type transport system periplasmic substrate-binding protein (probable substrate iron-III)	HVO_B0198	Unknown	on	hvo02010 - ABC transporters	
m	ABC-type transport system periplasmic substrate-binding protein (probable substrate iron-III)	HVO_B0047	Extra-cellular	on	hvo02010 - ABC transporters	
m	putative phosphate ABC transporter periplasmic substrate-binding protein	HVO_2375	C/M	on	hvo02010 - ABC transporters	
m	putative zinc ABC transporter periplasmic substrate-binding protein	HVO_2397	C/M	on	hvo02010 - ABC transporters	
m	putative iron-III ABC transporter periplasmic substrate-binding protein	HVO_1705	Unknown	on	hvo02010 - ABC transporters;	
c	TATA-binding transcription initiation factor	HVO_B0382	C	on	hvo03022 - Basal transcription factors;	
m	ATP-dependent RNA helicase/nuclease Hef	HVO_3010	Unknown	on		COG1111 Code L ERCC4-like helicases
m	flavoprotein reductase-like protein	HVO_0105	C/M	on		COG: COG1252 Code C NADH dehydrogenase, FAD-containing subunit
m	FxsA-like protein	HVO_2351	C/M	on		COG3030 Code R Protein affecting phage T7 exclusion by the F plasmid
m	hypothetical	HVO_0307	Unknown	on		
m	hypothetical	HVO_0129	C/M	on		
m	hypothetical	HVO_1010	C/M	on		
m	hypothetical	HVO_1307	C/M	on		
m	hypothetical	HVO_1850	C/M	on		
m	hypothetical	HVO_2513	C/M	on		
m	hypothetical	HVO_A0316	C/M	on		
m	Tat (twin-arginine translocation) pathway signalsequence domain protein	HVO_C0054	Unknown	on		
c	UspA domain protein	HVO_A0047	C	on		COG0589 Code T Universal stress protein UspA and related nucleotide-binding proteins

c	UspA domain-containing protein	HVO_1198	C	on		COG0589 Code T Universal stress protein UspA and related nucleotide-binding proteins
m	cationic amino acid transporter	HVO_A0175	C/M	**-3.5**		COG0531 Code E Amino acid transporters
c	DNA polymerase D polymerase subunit DP2	HVO_0065	C	**-3.5**	hvo00230 - Purine metabolism; hvo00240 - Pyrimidine metabolism	
c	PQQ repeat protein	HVO_B0052	Unknown	**-2.7**		COG1520 Code S FOG: WD40-like repeat
c	*tryptophan synthase subunit beta*	*HVO_0788*	*C*	**-2.2**	*hvo00260 - Glycine, serine and threonine metabolism; hvo00400 - Phenylalanine, tyrosine and tryptophan biosynthesis; hvo01110 - Biosynthesis of secondary metabolites;*	
c	acyl-CoA dehydrogenase	HVO_1199	C	-1.8		COG1960 Code I Acyl-CoA dehydrogenases
m	cytochrome d ubiquinol oxidase subunit II	HVO_0461	C/M	-1.8	hvo00190 - Oxidative phosphorylation	
m	hypothetical	HVO_B0359	C/M	-1.8		
c	*tryptophan synthase subunit alpha*	*HVO_0789*	*C*	*-1.8*	*hvo00260 - Glycine, serine and threonine metabolism; hvo00400 - Phenylalanine, tyrosine and tryptophan biosynthesis; hvo01110 - Biosynthesis of secondary metabolites; hvo00400 - Phenylalanine, tyrosine and tryptophan biosynthesis; hvo01110 - Biosynthesis of secondary metabolites;*	
c	isopentenyl-diphosphate delta-isomerase	HVO_2506	C	-1.8	hvo00900 - Terpenoid backbone biosynthesis; hvo01110 - Biosynthesis of secondary metabolites;	
c	50S ribosomal protein L1	HVO_2757	C	-1.8	hvo03010 - Ribosome	
c	50S ribosomal protein L15.eR	HVO_0561	C	-1.8	hvo03010 - Ribosome	
m	PQQ repeat-containing protein	HVO_2606	Unknown	-1.8		COG1520 Code S FOG: WD40-like repeat
c	3-ketoacyl-CoA thiolase	HVO_1914	C	-1.7	hvo00071 - Fatty acid metabolism; hvo00280 - Valine, leucine and isoleucine degradation; hvo00281 - Geraniol degradation; hvo000362 - Benzoate degradation; hvo00592 - alpha-Linolenic acid metabolism; hvo01110 - Biosynthesis of secondary metabolites;	
c	CbiG	HVO_B0059	C	-1.7	hvo00860 - Porphyrin and chlorophyll metabolism;	
m	hypothetical	HVO_1103	C/M	-1.7		
c	hypothetical protein	HVO_2203	Unknown	-1.7		
c	DNA N-glycosylase	HVO_1681	C	-1.6	hvo03410 - Base excision repair;	
c	propionyl-CoA carboxylase complex B chain	HVO_1447	C	-1.6	hvo00280 - Valine, leucine and isoleucine degradation; hvo00630 - Glyoxylate and dicarboxylate metabolism; hvo00640 - Propanoate metabolism; hvo01120 - Microbial metabolism in diverse environments;	
m	heavy-metal transporting CPx-type ATPase	HVO_0940	C/M	-1.6		OG2217 Code P Cation transport ATPase
m	SpoIVFB-type metallopeptidase	HVO_0285	C/M	-1.5		OG1994 Code R Zn-dependent proteases
c	carbamoyl-phosphate synthase large subunit	HVO_2361	C	-1.4	hvo00240 - Pyrimidine metabolism; hvo00250 - Alanine, aspartate and glutamate metabolism;	
c	acyl-CoA synthetase	HVO_0894	C	-1.4	hvo00010 - Glycolysis / Gluconeogenesis; hvo00620 - Pyruvate metabolism; hvo00640 - Propanoate metabolism; hvo00680 - Methane metabolism; hvo01110 - Biosynthesis of secondary metabolites	

c	ATP-dependent DNA helicase	HVO_1333	C	-1.4		COG1201 Code R Lhr-like helicases
c	L-fuculose phosphate aldolase	HVO_A0268	C	-1.4	hvo00051 - Fructose and mannose metabolism	
c	precorrin-3B C17-methyltransferase	HVO_B0057	C	-1.3	hvo00860 - Porphyrin and chlorophyll metabolism;	
c	diphthamide synthase subunit	HVO_1631	C	-1.3		COG0411 Code E ABC-type branched-chain amino acid transport systems, ATPase component
m	NADH dehydrogenase-like complex subunit B	HVO_0979	C/M	-1.3	hvo00190 - Oxidative phosphorylation	
c	ornithine carbamoyltransferase	HVO_0041	C	-1.3		COG0078 Code E Ornithine carbamoyltransferase
c	GTP cyclohydrolase III 1	HVO_1284	C	-1.3	hvo00740 - Riboflavin metabolism	
m	ba3-type terminal oxidase subunit II	HVO_0944	C/M	-1.3	hvo00190 - Oxidative phosphorylation	
c	acyl-CoA synthetase	HVO_1374	C	-1.3		COG0318 Code IQ Acyl-CoA synthetases (AMP-forming)/AMP-acid ligases II
m	Manganese transport protein mntH	HVO_2674	C/M	-1.3		COG1914 Code P Mn2+ and Fe2+ transporters of the NRAMP family
m	putative dipeptides/oligopeptides ABC transporter ATP-binding protein	HVO_0627	C/M	-1.3	hvo02010 - ABC transporters	
c	glycosyltransferase AglI	HVO_1528	C	-1.3		COG1215 Code M Glycosyltransferases, probably involved in cell wall biogenesis
c	*acyl-CoA dehydrogenase*	*HVO_0209*	*C*	*-1.3*	*hvo00071 - Fatty acid metabolism; hvo00310 - Lysine degradation; hvo00362 - Benzoate degradation; hvo00380 - Tryptophan metabolism; hvo01120 - Microbial metabolism in diverse environments;*	
m	FAD-linked oxidase domain-containing protein	HVO_1697	C/M	-1.2		COG0277 Code C FAD/FMN-containing dehydrogenases
c	GTP-binding protein Era	HVO_3014	C	-1.2		COG1100 Code R GTPase SAR1 and related small G proteins
c	cystathionine gamma-synthase	HVO_2946	C	-1.2	hvo00260 - Glycine, serine and threonine metabolism; hvo00270 - Cysteine and methionine metabolism; hvo00450 - Selenoamino acid metabolism; hvo00910 - Nitrogen metabolism;	
c	farnesyl-diphosphate farnesyltransferase	HVO_1139	C	-1.2	; hvo01110 - Biosynthesis of secondary metabolites	
m	hypothetical	HVO_0453	C/M	-1.2		
c	putative inositol-1(or 4)-monophosphatase / fructose-1,6-bisphosphatase, archaeal type	HVO_2857	C	-1.2	hvo00521 - Streptomycin biosynthesis; hvo00562 - Inositol phosphate metabolism; hvo01110 - Biosynthesis of secondary metabolites	
c	glutamate dehydrogenase	HVO_B0266	C	-1.2	hvo00250 - Alanine, aspartate and glutamate metabolism; hvo00330 - Arginine and proline metabolism; hvo00910 - Nitrogen metabolism;	
c	phosphoribosylamine--glycine ligase	HVO_1657	C	-1.2	hvo00230 - Purine metabolism; hvo01110 - Biosynthesis of secondary metabolites	
c	malate synthase	HVO_B0200	C	-1.1		COG2301 Code G Citrate lyase beta subunit
c	DJ-1/PfpI/ThiJ superfamily protein	HVO_1073	C	-1.1		COG0693 Code R Putative intracellular protease/amidase
c	*anthranilate phosphoribosyltransferase*	*HVO_2456*	*C*	*-1.1*	*hvo00400 - Phenylalanine, tyrosine and tryptophan biosynthesis; hvo01110 - Biosynthesis of secondary metabolites*	
c	MiaB-like tRNA modifying enzyme, archaeal-type	HVO_2605	C	-1.1		COG0621 Code J 2-methylthioadenine synthetase

m	PBS lyase HEAT-like repeat domain-containing protein	HVO_1020	Unknown	-1.1		COG1413 Code C FOG: HEAT repeat
c	precorrin-3B C17-methyltransferase	HVO_B0058	Unknown	-1.1	hvo00860 - Porphyrin and chlorophyll metabolism;	
c	cobyric acid synthase CobQ	HVO_A0553	C	-1.1	hvo00860 - Porphyrin and chlorophyll metabolism;	
c	repair helicase	HVO_0415	C	-1.1	hvo03420 - Nucleotide excision repair; hvo03430 - Mismatch repair	
c	uridine phosphorylase	HVO_2614	C	-1.1	hvo00240 - Pyrimidine metabolism	
m	hypothetical	HVO_0941	C/M	-1.0		
c	acyl-CoA synthetase	HVO_1585	C	-1.0	hvo00010 - Glycolysis / Gluconeogenesis; hvo00620 - Pyruvate metabolism; hvo00640 - Propanoate metabolism; hvo00680 - Methane metabolism; hvo01110 - Biosynthesis of secondary metabolites	
c	methylmalonyl-CoA mutase subunit A	HVO_0893	C	-1.0	hvo00280 - Valine, leucine and isoleucine degradation; hvo00640 - Propanoate metabolism; hvo01120 - Microbial metabolism in diverse environments;	
c	DNA binding protein	HVO_0662	C	-1.0		OG1992 Code S Uncharacterized conserved protein
c	phosphoribosylaminoimidazole-succinocarboxamide synthase	HVO_2193	C	-1.0	hvo00230 - Purine metabolism; hvo01110 - Biosynthesis of secondary metabolites	
c	pyrroline-5-carboxylate reductase	HVO_1372	C	-1.0	hvo00330 - Arginine and proline metabolism; hvo01110 - Biosynthesis of secondary metabolites	
c	quinolinate synthetase complex, A subunit	HVO_2581	C	-1.0	hvo00760 - Nicotinate and nicotinamide metabolism	
c	possible polygalacturonase, putative	HVO_B0085	C	-1.0		COG5434 Code M Endopolygalacturonase
m	hypothetical	HVO_1927	C/M	-1.0		
c	ferredoxin--nitrite reductase	HVO_1788	C	-1.0	hvo00910 - Nitrogen metabolism; hvo01120 - Microbial metabolism in diverse environments	
c	aminotransferase class V	HVO_A0485	C	-1.0		COG0075 Code E Serine-pyruvate aminotransferase/archaeal aspartate aminotransferase
c	FolD bifunctional protein	HVO_2865	C	-1.0	hvo00670 - One carbon pool by folate; hvo01120 - Microbial metabolism in diverse environments	
m	cyanide insensitive terminal oxidase chain cioA	HVO_0462	C/M	-1.0	hvo00190 - Oxidative phosphorylation	
c	diphosphomevalonate decarboxylase	HVO_1412	C	-0.9	hvo00900 - Terpenoid backbone biosynthesis; hvo01110 - Biosynthesis of secondary metabolites	
c	50S ribosomal protein L10.eR	HVO_0484	C	-0.9	hvo03010 - Ribosome	
c	Fic protein family, putative	HVO_A0401	C	-0.9		**COG:** COG3177 Code S Uncharacterized conserved protein
c	50S ribosomal protein L18	HVO_2545	Unknown	-0.9	hvo03010 - Ribosome	
c	ArcR family transcription regulator	HVO_A0266	Unknown	-0.9	ArcR transcriptional regulator	COG1414 Code K Transcriptional regulator
c	FAD binding domain	HVO_2580	C	-0.9	hvo00250 - Alanine, aspartate and glutamate metabolism; hvo00760 - Nicotinate and nicotinamide metabolism	
c	citrate synthase	HVO_0466	C	-0.9	hvo00020 - Citrate cycle (TCA cycle); hvo00630 - Glyoxylate and dicarboxylate metabolism; hvo01110 - Biosynthesis of secondary metabolites	
c	30S ribosomal protein S4	HVO_2783	C	-0.9	hvo03010 - Ribosome	

c	hypothetical protein	HVO_2080	Unknown	-0.9		
m	HTR-like protein	HVO_A0160	C/M	-0.8		COG0642 Code T Signal transduction histidine kinase
c	aspartyl-tRNA(Asn) amidotransferase subunit A	HVO_1054	C	-0.8	hvo00970 - Aminoacyl-tRNA biosynthesis	
m	cation-transporting ATPase	HVO_0933	C/M	-0.8		OG0474 Code P Cation transport ATPase
c	cell surface glycoprotein	HVO_2072	Cellwall	-0.8		
c	*anthranilate synthase component I*	*HVO_2454*	*C*	*-0.8*	*hvo00400 - Phenylalanine, tyrosine and tryptophan biosynthesis; hvo01110 - Biosynthesis of secondary metabolites*	
c	deoxyhypusine synthase	HVO_B0182	C	-0.8		COG1899 Code O Deoxyhypusine synthase
c	*tryptophanase*	*HVO_0009*	*C*	***4.3***	*hvo00380 - Tryptophan metabolism; hvo00910 - Nitrogen metabolism;*	
m	hypothetical	HVO_B0064	C/M	**4.2**		
c	putative zinc ABC transporter ATP-binding protein	HVO_2398	C	**2.5**	hvo02010 - ABC transporters	
m	cox-type terminal oxidase subunit I	HVO_0907	C/M	**2.4**	hvo00190 - Oxidative phosphorylation	
c	halocyanin	HVO_1228	C	**2.4**		COG3794 Code C Plastocyanin
c	hypothetical protein	HVO_0377	Unknown	**2.4**		
m	pantothenate permease	HVO_2324	C/M	**2.1**		COG0591 Code ER Na+/proline symporter
c	translation initiation factor aIF-5A	HVO_2300	C	2.0		COG0231 Code J Translation elongation factor P (EF-P)/translation initiation factor 5A (eIF-5A)
m	hypothetical	HVO_0229	C/M	1.8		
c	sugar nucleotidyltransferase	HVO_A0586	C	1.8	hvo00520 - Amino sugar and nucleotide sugar metabolism	
c	coenzyme PQQ synthesis protein E-like protein	HVO_1121	C	1.8		COG0535 Code R Predicted Fe-S oxidoreductases
m	preprotein translocase Sec61 alpha subunit	HVO_A0174	C/M	1.8	hvo03060 - Protein export; hvo03070 - Bacterial secretion system	
c	putative iron-III ABC transporter periplasmic substrate-binding protein	HVO_1705	Unknown	1.7	hvo02010 - ABC transporters	
c	pyridine nucleotide-disulfide oxidoreductase, class II, putative	HVO_A0501	Unknown	1.7		COG2226 Code H Methylase involved in ubiquinone/menaquinone biosynthesis
c	UspA domain protein	HVO_A0496	C	1.7		COG0589 Code T Universal stress protein UspA and related nucleotide-binding proteins
c	hypothetical protein	HVO_A0633	Unknown	1.7		
m	anion permease	HVO_2996	C/M	1.7		COG0306 Code P Phosphate/sulphate permeases
c M S	ABC-type transport system periplasmic substrate-binding protein (probable substrate dipeptides/oligopeptides)	HVO_A0339	Cellwall	1.6	hvo02010 - ABC transporters	

c	hypothetical protein	HVO_2327	C	1.6		
m	preprotein translocase Sec61 subunit gamma	HVO_0718	C/M	1.6	hvo03060 - Protein export	
c	dioxgenase	HVO_2662	C	1.6		COG0346 Code E Lactoylglutathione lyase and related lyases
c	cobyrinic acid ac-diamide synthase	HVO_0999	C	1.5		COG0857 Code R BioD-like N-terminal domain of phosphotransacetylase
c	*anthranilate phosphoribosyltransferase*	*HVO_2226*	*C*	*1.5*	*hvo00400 - Phenylalanine, tyrosine and tryptophan biosynthesis; vo01110 - Biosynthesis of secondary metabolites;*	
c	deoxyribodipyrimidine photolyase	HVO_2911	C	1.5		COG0415 Code L Deoxyribodipyrimidine photolyase
m	1,4-dihydroxy-2-naphthoateoctaprenyltransferase	HVO_1462	C/M	1.5	hvo00130 - Ubiquinone and other terpenoid-quinone biosynthesis; hvo01110 - Biosynthesis of secondary metabolites;	
m	A-type ATP synthase subunit I	HVO_0311	C/M	1.4	hvo00190 - Oxidative phosphorylation; hvo00680 - Methane metabolism;	
m	hypothetical	HVO_1182	C/M	1.4		
m	glycosyltransferase AglD	HVO_0798	C/M	1.4		COG0463 Code M Glycosyltransferases involved in cell wall biogenesis
c	thioredoxin reductase	HVO_1758	C	1.4		COG0492 Code O Thioredoxin reductase
c	HpcH/HpaI aldolase family protein	HVO_2665	C	1.4		OG3836 Code G 2,4-dihydroxyhept-2-ene-1,7-dioic acid aldolase
m	putative dipeptides/oligopeptides ABC transporter permease	HVO_0629	C/M	1.4	hvo02010 - ABC transporters	
m	hypothetical	HVO_1274	C/M	1.4		
m	xanthine/uracil permease family protein	HVO_0335	C/M	1.3		COG2252 Code R Permeases
m	dolichyl-phosphate-mannose-proteinmannosyltransfe rase	HVO_0975	C/M	1.3		COG4745 Code O Predicted m-bound mannosyltransferase
c	O-acetylhomoserine aminocarboxypropyltransferase	HVO_2997	C	1.3	hvo00270 - Cysteine and methionine metabolism;	
c	4-hydroxythreonine-4-phosphate dehydrogenase	HVO_2111	C	1.3	hvo00750 - Vitamin B6 metabolism	
c	NADH dehydrogenase/oxidoreductase-like protein	HVO_2205	C	1.3	hvo00190 - Oxidative phosphorylation;	
c	molybdopterin biosynthesis protein moeA	HVO_2304	C	1.3		COG0303 Code H Molybdopterin biosynthesis enzyme
c	NADH-quinone oxidoreductase chain c/d	HVO_0968	C	1.3	hvo00190 - Oxidative phosphorylation;	
c	chlorite dismutase family protein	HVO_1871	Unknown	1.2		COG3253 Code S Uncharacterized conserved protein
c	cetyl-CoA synthetase	HVO_1000	C	1.2		COG1042 Code C Acyl-CoA synthetase (NDP forming)
c	SAM-dependent methyltransferase	HVO_1389	Unknown	1.2		COG2226 Code H Methylase involved in ubiquinone/menaquinone biosynthesis
c	ba3-type terminal oxidase subunit CbaD	HVO_0943	Unknown	1.2		

c	molybdopterin biosynthesis protein moeA	HVO_2305	C	1.2		COG0303 Code H Molybdopterin biosynthesis enzyme
c M S	hypothetical	HVO_1431	Extra-cellular	1.2		
m	gluconate permease GntP	HVO_2190	C/M	1.2		COG2610 Code GE H+/gluconate symporter and related permeases
c	hypothetical protein	HVO_2770	Unknown	1.1		
m	ABC-type transport system permease protein (probable substrate dipeptides/oligopeptides)	HVO_B0081	C/M	1.1	hvo02010 - ABC transporters	
c	HydD	HVO_1568	Unknown	1.1		COG0596 Code R Predicted hydrolases or acyltransferases (alpha/beta hydrolase superfamily)
m	twin arginine translocation system subunit TatAt	HVO_1162	Unknown	1.1	hvo03060 - Protein export; hvo03070 - Bacterial secretion system	
c	UspA domain-containing protein	HVO_2156	C	1.1		COG0589 Code T Universal stress protein UspA and related nucleotide-binding proteins
c	CBS domain pair	HVO_2384	C	1.1		COG3448 Code T CBS-domain-containing m protein; homolog=chloride channel
c	hypothetical protein	HVO_1749	Unknown	1.1		
c	threonine ammonia-lyase	HVO_0464	C	1.0	hvo00260 - Glycine, serine and threonine metabolism; hvo00290 - Valine, leucine and isoleucine biosynthesis; hvo01110 - Biosynthesis of secondary metabolites	
m	putative phosphate ABC transporter ATP-binding protein	HVO_2378	C/M	1.0	hvo02010 - ABC transporters	
m	ABC-type transport system permease protein (probable substrate dipeptides/oligopeptides)	HVO_B0080	C/M	1.0	hvo02010 - ABC transporters	
c	ATP-binding protein Mrp	HVO_2790	C	1.0		COG0489 Code D ATPases involved in chromosome partitioning
c	sugar nucleotidyltransferase	HVO_1076	C	1.0	hvo00521 - Streptomycin biosynthesis; hvo00523 - Polyketide sugar unit biosynthesis; hvo01110 - Biosynthesis of secondary metabolites	
c M S	hypothetical	HVO_0972	Extra-cellular	1.0		
c	hypothetical protein	HVO_1660	C	0.9		
c	dihydrolipoyl dehydrogenase	HVO_2961	C	0.9	hvo00010 - Glycolysis / Gluconeogenesis; hvo00020 - Citrate cycle (TCA cycle); hvo00260 - Glycine, serine and threonine metabolism; hvo00280 - Valine, leucine and isoleucine degradation; hvo00620 - Pyruvate metabolism; hvo01110 - Biosynthesis of secondary metabolites	
c	glutamate dehydrogenase	HVO_1453	C	0.9	hvo00250 - Alanine, aspartate and glutamate metabolism; hvo00330 - Arginine and proline metabolism; hvo00910 - Nitrogen metabolism;	
c	ATP phosphoribosyltransferase	HVO_0161	C	0.9	hvo00340 - Histidine metabolism; hvo01110 - Biosynthesis of secondary metabolites	
c	peptidyl-prolyl cis-trans isomerase	HVO_1637	C	0.9		COG1047 Code O FKBP-type peptidyl-prolyl cis-trans isomerases 2
c	indole-3-acetyl-L-aspartic acid hydrolase	HVO_1395	C	0.9		COG1473 Code R Metal-dependent amidase/aminoacylase/carboxypeptidase

c<br>M<br>S	aminopeptidase	HVO_0836	Extra-cellular	0.9		OG2234 Code R Predicted aminopeptidases
m	hypothetical	HVO_0958	C/M	0.9		
c	TATA-binding transcription initiation factor	HVO_1727	C	0.9	hvo03022 - Basal transcription factors	
m	ABC transporter permease	HVO_2084	C/M	0.9		COG4591 Code M ABC-type transport system, involved in lipoprotein release, permease component
c	hypothetical protein	HVO_A0181	Unknown	0.9		
m	hypothetical	HVO_0702	C/M	0.9		
c	cysteine desulfurase	HVO_0109	C	0.9	hvo00450 - Selenoamino acid metabolism; hvo00730 - Thiamine metabolism	
c	phenylalanyl-tRNA synthetase subunit alpha	HVO_2948	C	0.9	hvo00970 - Aminoacyl-tRNA biosynthesis	
c<br>M<br>S	ABC-type transport system periplasmic substrate-binding protein (probable substrate dipeptides/oligopeptides)	HVO_A0380	Cellwall	0.9	hvo02010 - ABC transporters	
m	Na+/H+ antiporter NhaC	HVO_1394	C/M	0.8		OG1757 Code C Na+/H+ antiporter
m	sugar transporter	HVO_2097	C/M	0.8		COG2223 Code P Nitrate/nitrite transporter
c	hypothetical protein	HVO_1803	Unknown	0.8		
c	imidazoleglycerol phosphate synthase, cyclase subunit	HVO_1044	C	0.8	hvo00340 - Histidine metabolism; hvo01110 - Biosynthesis of secondary metabolites	
c	putative yjeF family carbohydrate kinase	HVO_1348	C	0.8		COG0062 Code S Uncharacterized conserved protein
c	thioredoxin-disulfide reductase	HVO_1123	C	0.8	hvo00240 - Pyrimidine metabolism; hvo00450 - Selenoamino acid metabolism	
c	2-oxo-3-methylvalerate dehydrogenase E1 component subunit alpha	HVO_2958	C	0.8	hvo00010 - Glycolysis / Gluconeogenesis; hvo00020 - Citrate cycle (TCA cycle); hvo00290 - Valine, leucine and isoleucine biosynthesis; hvo00620 - Pyruvate metabolism; hvo00650 - Butanoate metabolism; hvo01110 - Biosynthesis of secondary metabolites	
c	phosphoenolpyruvate-protein phosphotransferase	HVO_1496	C	0.8	hvo02060 - Phosphotransferase system (PTS)	
m	putative glutamine ABC transporter permease	HVO_2431	C/M	0.8		COG0765 Code E ABC-type amino acid transport system, permease component
c	pyruvate--ferredoxin oxidoreductase subunit alpha	HVO_1305	C	0.8	hvo00020 - Citrate cycle (TCA cycle); hvo01120 - Microbial metabolism in diverse environments	
c	metal-dependent carboxypeptidase	HVO_0417	C	0.8		COG2317 Code E Zn-dependent carboxypeptidase

Supplementary Table 3. Strains

Strain	Genotype	Source
DH5α	F^-, φ80d*lacZ*ΔM15, Δ(*lacZYA-argF*)U169, *deoR*, *recA*1, *endA*1, *hsdR*17(r_k^-, m_k^+), *phoA*, *supE*44,λ^-, *thi*-1, *gyrA*96, *relA*1	Invitrogen
H119	DS70 (ΔpHV2), Δ*pyrE*2, Δ*trpA*, Δ*leuB*	Allers et al., 2004
H66	DS70 (ΔpHV2), Δ*pyrE*2, Δ*leuB*	Allers et al., 2004
Δ*s479*	DS70 (ΔpHV2), Δ*pyrE2*, Δ*trpA*, Δ*leuB*, Δ*s479::trpA*$^+$	Jaschinski et al., 2014
Δ*cas6*	Δ*pyr*E2, Δ*trp*A, Δ*leu*B, Δ*cas6*	Brendel et al., 2014
Δ*cas7*	Δ*pyr*E2, Δ*trp*A, Δ*leu*B, Δ*cas*7	Brendel et al., 2014

Supplementary Table 4. Oligonucleotides and Sequencing Barcodes

Primer	Sequence	Used for
TruSeq_Sense_primer	5'-AATGATACGGCGACCACCGAGATCTACAC-NNNNNNNN-ACACTCTTTCCCTACACGACGCTCTTCCGATCT-3'; NNN=i5 Barcode	RNA-Seq
TruSeq_Antisense_primer	5'-CAAGCAGAAGACGGCATACGAGAT-NNNNNNNN-GTGACTGGAGTTCAGACGTGTGCTCTTCCGATCT-3'; NNN= i7 Index	RNA-Seq
Delta-s479-Nr1	i5 Barcode AGGCTATA	RNA-Seq
	i7 Index ATTACTCG	
Delta-s479-Nr2	i5 Barcode GCCTCTAT	RNA-Seq
	i7 Index ATTACTCG	
Delta-s479-Nr3	i5 Barcode GTCAGTAC	RNA-Seq
	i7 Index ATTACTCG	
H66-Nr1	i5 Barcode AGGATAGG	RNA-Seq
	i7 Index ATTACTCG	
H66-Nr2	i5 Barcode TCAGAGCC	RNA-Seq
	i7 Index ATTACTCG	
H66-Nr3	i5 Barcode ATAGAGAG	RNA-Seq
	i7 Index ATTACTCG	
probe znuC1 Hvo_2398fw	CCGCGAGGTCGTGAAGATGGGTCGG	northern blot
probe znuC1 Hvo_2398rev	AGGTCGTGTTCGATGAGGAGAATCG	northern blot
16Sseqf	CGCTAGGTGTGACACAGGCTACG	northern blot
16Sseqrev	GTGAGATGTCCGGCGTTGAGTCC	northern blot
Hvo5Sp	CGCAGGTGAGCTTAACTTCCGTGTTCGGG	northern blot
s479spacerpart	CGCTTCGGCTGATGGGTGTCATGAGGTTTCCCTTGTCAATACTT	northern blot
s479 RNA	AAGUAUUGACAAGGGAAACCUCAUGACACCCAUCAGCCGAAGCGACAAACUGGGU	EMSA
Znucmid RNA	GGUCGUGAAGAUGGGUCGGUUCCCCCACGUCGGCUUCGGCCGGCUCUCGGCCGAAGACCACCGCAUCGUCGACGAGGCGC	EMSA
Znucmid mutant RNA	GGUCGUGAAGAUGGGUCGGUUCCCCCAC-CGGCUCUCGGCCGAAGACCACCGCAUCGUCGACGAGGCGC	EMSA

Supplementary Table 5. Data and calculations for growth experiments at standard zinc concentration. Supplementary Table 5A1. and 5A2. list the data used to compile the growth curves in Figure 4. Supplementary Table 5B. shows the calculations of growth rate and doubling time for wildtype and deletion strain (Δ*s479*). Supplementary Table 5C1. and 5C2. list all the raw data collected for the growth experiments. For better readability, all numbers were cut to three decimals or less.

Supplementary Table 5A1. Mean growth and standard deviation used to compile the wildtype strain (H66) growth curve in Figure 4. The mean OD_{650nm} is given for all three biological replicates of wildtype strain H66 (H66-1 to H66-3) alongside the overall mean OD_{650nm} and standard deviation per time interval.

H66-1	H66-2	H66-3	H66	H66	
**Mean OD650**	**Mean OD650**	**Mean OD650**	**Mean OD650**	**SD**	**time [h]**
0.066	0.068	0.065	**0.066**	0.001	**0.5**
0.100	0.099	0.094	**0.098**	0.003	**2.5**
0.147	0.143	0.130	**0.140**	0.007	**4.5**
0.201	0.196	0.175	**0.190**	0.011	**6.5**
0.246	0.248	0.229	**0.241**	0.009	**8.5**
0.289	0.297	0.287	**0.291**	0.004	**10.5**
0.336	0.342	0.344	**0.340**	0.003	**12.5**
0.372	0.385	0.391	**0.383**	0.008	**14.5**
0.405	0.431	0.430	**0.422**	0.012	**16.5**
0.441	0.477	0.462	**0.460**	0.015	**18.5**
0.480	0.516	0.490	**0.496**	0.015	**20.5**
0.520	0.549	0.514	**0.528**	0.015	**22.5**
0.551	0.575	0.534	**0.554**	0.017	**24.5**
0.574	0.595	0.552	**0.574**	0.017	**26.5**
0.592	0.611	0.567	**0.590**	0.018	**28.5**
0.607	0.622	0.579	**0.603**	0.018	**30.5**
0.618	0.632	0.591	**0.614**	0.017	**32.5**
0.630	0.642	0.601	**0.624**	0.017	**34.5**
0.640	0.650	0.610	**0.633**	0.017	**36.5**
0.649	0.659	0.619	**0.642**	0.017	**38.5**
0.657	0.665	0.628	**0.650**	0.016	**40.5**
0.663	0.671	0.636	**0.656**	0.015	**42.5**
0.668	0.676	0.643	**0.662**	0.014	**44.5**
0.668	0.678	0.649	**0.665**	0.012	**46.5**
0.666	0.681	0.655	**0.667**	0.011	**48.5**
0.669	0.681	0.660	**0.670**	0.009	**50.5**
0.672	0.684	0.664	**0.673**	0.008	**52.5**
0.675	0.688	0.667	**0.677**	0.008	**54.5**
0.677	0.689	0.671	**0.679**	0.008	**56.5**
0.680	0.691	0.673	**0.681**	0.007	**58.5**
0.684	0.696	0.677	**0.685**	0.008	**60.5**
0.686	0.698	0.679	**0.688**	0.008	**62.5**
0.689	0.700	0.683	**0.691**	0.007	**64.5**
0.692	0.703	0.686	**0.694**	0.007	**66.5**
0.696	0.705	0.690	**0.697**	0.006	**68.5**
0.700	0.706	0.693	**0.700**	0.005	**70.5**
0.697	0.707	0.695	**0.700**	0.005	**72.5**

Supplementary Table 5A2. Mean growth and standard deviation used to compile the deletion strain (Δ*s479*) growth curve in Figure 4. The mean OD_{650nm} is given for all three biological replicates of deletion strain Δ*s479* (s479-1 to s479-3) alongside the overall mean OD_{650nm} and standard deviation per time interval.

s479-1	s479-2	s479-3	s479	s479 SD	
**Mean OD650**	**Mean OD650**	**Mean OD650**	**Mean OD650**		**time [h]**
0.065	0.055	0.064	**0.061**	0.004	**0.5**
0.101	0.086	0.100	**0.096**	0.007	**2.5**
0.142	0.120	0.138	**0.133**	0.010	**4.5**
0.192	0.162	0.187	**0.180**	0.013	**6.5**
0.251	0.210	0.243	**0.235**	0.018	**8.5**
0.306	0.260	0.295	**0.287**	0.020	**10.5**
0.352	0.305	0.337	**0.331**	0.020	**12.5**
0.387	0.341	0.371	**0.366**	0.019	**14.5**
0.413	0.373	0.401	**0.396**	0.017	**16.5**
0.437	0.401	0.429	**0.422**	0.015	**18.5**
0.457	0.426	0.457	**0.447**	0.015	**20.5**
0.478	0.448	0.481	**0.469**	0.015	**22.5**
0.495	0.468	0.501	**0.488**	0.014	**24.5**
0.510	0.484	0.519	**0.505**	0.015	**26.5**
0.522	0.498	0.535	**0.518**	0.015	**28.5**
0.534	0.511	0.549	**0.531**	0.015	**30.5**
0.543	0.523	0.561	**0.542**	0.015	**32.5**
0.553	0.534	0.572	**0.553**	0.015	**34.5**
0.561	0.543	0.583	**0.562**	0.016	**36.5**
0.568	0.553	0.592	**0.571**	0.016	**38.5**
0.577	0.561	0.602	**0.580**	0.017	**40.5**
0.584	0.568	0.610	**0.587**	0.017	**42.5**
0.590	0.576	0.624	**0.597**	0.020	**44.5**
0.597	0.582	0.635	**0.605**	0.022	**46.5**
0.602	0.590	0.639	**0.610**	0.021	**48.5**
0.608	0.597	0.640	**0.615**	0.018	**50.5**
0.612	0.601	0.638	**0.617**	0.015	**52.5**
0.615	0.604	0.642	**0.620**	0.016	**54.5**
0.617	0.607	0.648	**0.624**	0.017	**56.5**
0.619	0.610	0.654	**0.628**	0.019	**58.5**
0.619	0.613	0.659	**0.630**	0.020	**60.5**
0.621	0.614	0.666	**0.634**	0.023	**62.5**
0.623	0.615	0.670	**0.636**	0.024	**64.5**
0.623	0.617	0.672	**0.637**	0.024	**66.5**
0.625	0.619	0.670	**0.638**	0.023	**68.5**
0.626	0.620	0.668	**0.638**	0.021	**70.5**
0.626	0.621	0.667	**0.638**	0.021	**72.5**

Supplementary Table 5B. Calculation of growth rate and doubling time at standard zinc concentration. Growth rate and doubling time were calculated as growth rate µ = (ln(xt) – ln (x0)) / (t – t0) and doubling time d= ln(2) / µ. Calculations were carried out separately for all replicates before calculating mean value (red) and standard deviation (red). Phases of exponential growth were identified using fitted trendlines and corresponding R^2-values (Supplementary Figure 5C.). Values are given separately for H66 (grey) and Δ*s479* (orange) and for each of the two phases.

phase1 0.5 to 10.5 h

growth rate µ(H66) [h^{-1}] time interval 0.5 h to 10.5 h with R² = 0.983

**H66-1**	**H66-2**	**H66-3**	**µ(H66) [h^{-1}]**	**SD(µ(H66)) [h^{-1}]**
0.147	0.148	0.149	**0.148**	**0.001**

doubling time / generation time d(H66) [h]

**H66-1**	**H66-2**	**H66-3**	**d(H66) [h]**	**SD(d(H66)) [h]**
4.7	4.7	4.7	**4.7**	**0.0**

growth rate µ(s479) [h^{-1}] time interval 0.5 h to 10.5 h with R² = 0.9836

**s479-1**	**s479-2**	**s479-3**	**µ(s479) [h^{-1}]**	**SD(µ(s479)) [h^{-1}]**
0.155	0.155	0.153	**0.154**	**0.001**

doubling time / generation time d(H66) [h]

**s479-1**	**s479-2**	**s479-3**	**d(s479) [h]**	**SD(d(s479)) [h]**
4.5	4.5	4.5	**4.5**	**0.0**

phase2 14.5 to 24.5 h

growth rate µ(H66) [h^{-1}] time interval 14.5 h to 24.5 h with R² = 0.9857

**H66-1**	**H66-2**	**H66-3**	**µ(H66) [h^{-1}]**	**SD(µ(H66)) [h^{-1}]**
0.039	0.040	0.031	**0.037**	**0.004**

doubling time / generation time d(H66) [h]

**H66-1**	**H66-2**	**H66-3**	**d(H66) [h]**	**SD(d(H66)) [h]**
17.6	17.3	22.1	**19.0**	**2.2**

growth rate µ(s479) [h^{-1}] time interval 14.5 h to 24.5 h with R² = 0.9866

**s479-1**	**s479-2**	**s479-3**	**µ(s479) [h^{-1}]**	**SD(µ(s479)) [h^{-1}]**
0.025	0.032	0.030	**0.029**	**0.003**

doubling time / generation time d(H66) [h]

**s479-1**	**s479-2**	**s479-3**	**d(s479) [h]**	**SD(d(s479)) [h]**
28.1	21.8	23.1	**24.4**	**2.7**

Supplementary Table 5C1. Raw growth data measured for the wildtype strain (H66) at standard zinc concentration. For each time point, OD_{650nm} of three biological (replicate 1-3; H66-1 to H66-3), with three technical replicates (A-C) each, are given. Additionally, the mean OD_{650nm} over all technical replicates is given for each biological replicate.

	replicate 1					replicate 2					replicate 3			
	**H66-1A**	**H66-1B**	**H66-1C**	**H66-1**		**H66-2A**	**H66-2B**	**H66-2C**	**H66-2**		**H66-3A**	**H66-3B**	**H66-3C**	**H66-3**
**time [h]**	OD650	OD650	OD650	**Mean OD650**		OD650	OD650	OD650	**Mean OD650**		OD650	OD650	OD650	**Mean OD650**
**0.5**	0.068	0.061	0.07	0.066		0.065	0.066	0.073	0.068		0.069	0.066	0.059	0.065
**1**	0.074	0.065	0.075	0.071		0.068	0.071	0.079	0.073		0.073	0.071	0.063	0.069
**1.5**	0.083	0.074	0.084	0.080		0.079	0.079	0.087	0.082		0.082	0.08	0.071	0.078
**2**	0.094	0.084	0.093	0.090		0.088	0.089	0.097	0.091		0.092	0.089	0.079	0.087
**2.5**	0.101	0.095	0.103	0.100		0.097	0.097	0.104	0.099		0.099	0.097	0.086	0.094
**3**	0.112	0.106	0.111	0.110		0.106	0.107	0.113	0.109		0.108	0.106	0.094	0.103
**3.5**	0.124	0.119	0.121	0.121		0.117	0.119	0.122	0.119		0.117	0.115	0.101	0.111
**4**	0.137	0.132	0.133	0.134		0.129	0.131	0.132	0.131		0.127	0.125	0.109	0.120
**4.5**	0.15	0.146	0.146	0.147		0.142	0.144	0.144	0.143		0.135	0.136	0.119	0.130
**5**	0.163	0.159	0.16	0.161		0.155	0.158	0.154	0.156		0.146	0.147	0.128	0.140
**5.5**	0.178	0.174	0.174	0.175		0.17	0.171	0.165	0.169		0.156	0.159	0.138	0.151
**6**	0.191	0.186	0.186	0.188		0.184	0.185	0.178	0.182		0.168	0.171	0.148	0.162
**6.5**	0.204	0.198	0.201	0.201		0.199	0.197	0.191	0.196		0.18	0.184	0.16	0.175
**7**	0.217	0.207	0.214	0.213		0.214	0.21	0.205	0.210		0.192	0.197	0.17	0.186
**7.5**	0.23	0.215	0.228	0.224		0.228	0.221	0.219	0.223		0.205	0.211	0.183	0.200
**8**	0.242	0.223	0.244	0.236		0.241	0.231	0.233	0.235		0.219	0.228	0.195	0.214
**8.5**	0.255	0.229	0.254	0.246		0.255	0.241	0.249	0.248		0.234	0.243	0.209	0.229
**9**	0.267	0.234	0.271	0.257		0.268	0.252	0.264	0.261		0.247	0.26	0.224	0.244
**9.5**	0.28	0.239	0.285	0.268		0.28	0.261	0.282	0.274		0.26	0.276	0.237	0.258
**10**	0.294	0.243	0.298	0.278		0.294	0.268	0.294	0.285		0.275	0.292	0.251	0.273
**10.5**	0.309	0.247	0.312	0.289		0.305	0.277	0.31	0.297		0.288	0.308	0.265	0.287
**11**	0.325	0.253	0.326	0.301		0.314	0.284	0.323	0.307		0.3	0.323	0.28	0.301
**11.5**	0.344	0.257	0.341	0.314		0.324	0.293	0.336	0.318		0.313	0.337	0.294	0.315
**12**	0.363	0.261	0.352	0.325		0.338	0.303	0.351	0.331		0.328	0.354	0.307	0.330
**12.5**	0.379	0.263	0.366	0.336		0.35	0.311	0.364	0.342		0.342	0.368	0.321	0.344
**13**	0.392	0.268	0.376	0.345		0.361	0.321	0.376	0.353		0.353	0.381	0.333	0.356
**13.5**	0.404	0.27	0.389	0.354		0.371	0.331	0.388	0.363		0.366	0.393	0.348	0.369
**14**	0.415	0.275	0.397	0.362		0.383	0.341	0.398	0.374		0.376	0.403	0.36	0.380
**14.5**	0.426	0.281	0.408	0.372		0.394	0.352	0.41	0.385		0.387	0.414	0.371	0.391
**15**	0.436	0.287	0.416	0.380		0.407	0.366	0.419	0.397		0.396	0.425	0.381	0.401
**15.5**	0.442	0.294	0.427	0.388		0.419	0.379	0.43	0.409		0.408	0.433	0.391	0.411
**16**	0.451	0.302	0.434	0.396		0.43	0.39	0.437	0.419		0.418	0.441	0.402	0.420
**16.5**	0.458	0.312	0.445	0.405		0.441	0.404	0.449	0.431		0.427	0.45	0.412	0.430
**17**	0.466	0.323	0.452	0.414		0.451	0.418	0.458	0.442		0.438	0.456	0.422	0.439
**17.5**	0.47	0.337	0.461	0.423		0.464	0.432	0.465	0.454		0.446	0.464	0.431	0.447
**18**	0.476	0.352	0.469	0.432		0.475	0.448	0.472	0.465		0.455	0.473	0.439	0.456
**18.5**	0.479	0.366	0.477	0.441		0.486	0.464	0.48	0.477		0.461	0.479	0.446	0.462
**19**	0.484	0.38	0.485	0.450		0.496	0.477	0.488	0.487		0.469	0.486	0.455	0.470
**19.5**	0.492	0.397	0.493	0.461		0.506	0.492	0.496	0.498		0.476	0.493	0.462	0.477
**20**	0.497	0.411	0.499	0.469		0.515	0.502	0.501	0.506		0.482	0.5	0.469	0.484

**20.5**	0.503	0.431	0.507	0.480		0.524	0.516	0.508	0.516		0.489	0.506	0.476	0.490
**21**	0.507	0.453	0.513	0.491		0.531	0.526	0.513	0.523		0.497	0.511	0.481	0.496
**21.5**	0.512	0.472	0.52	0.501		0.541	0.538	0.521	0.533		0.502	0.517	0.488	0.502
**22**	0.517	0.493	0.527	0.512		0.549	0.55	0.526	0.542		0.508	0.523	0.494	0.508
**22.5**	0.521	0.508	0.532	0.520		0.557	0.559	0.532	0.549		0.515	0.528	0.5	0.514
**23**	0.525	0.523	0.538	0.529		0.564	0.568	0.537	0.556		0.519	0.534	0.504	0.519
**23.5**	0.532	0.532	0.544	0.536		0.569	0.574	0.544	0.562		0.525	0.538	0.512	0.525
**24**	0.538	0.544	0.55	0.544		0.576	0.583	0.548	0.569		0.529	0.543	0.516	0.529
**24.5**	0.543	0.556	0.555	0.551		0.581	0.591	0.554	0.575		0.534	0.548	0.521	0.534
**25**	0.548	0.566	0.56	0.558		0.586	0.597	0.557	0.580		0.539	0.553	0.526	0.539
**25.5**	0.552	0.572	0.565	0.563		0.591	0.603	0.563	0.586		0.544	0.558	0.531	0.544
**26**	0.558	0.577	0.569	0.568		0.596	0.607	0.567	0.590		0.547	0.561	0.535	0.548
**26.5**	0.563	0.585	0.574	0.574		0.599	0.613	0.572	0.595		0.552	0.565	0.539	0.552
**27**	0.568	0.59	0.579	0.579		0.604	0.619	0.576	0.600		0.556	0.569	0.543	0.556
**27.5**	0.572	0.596	0.583	0.584		0.608	0.623	0.577	0.603		0.56	0.573	0.547	0.560
**28**	0.576	0.6	0.586	0.587		0.611	0.625	0.583	0.606		0.563	0.575	0.552	0.563
**28.5**	0.58	0.605	0.591	0.592		0.615	0.632	0.586	0.611		0.567	0.579	0.555	0.567
**29**	0.584	0.609	0.595	0.596		0.617	0.633	0.589	0.613		0.57	0.581	0.559	0.570
**29.5**	0.587	0.613	0.599	0.600		0.619	0.637	0.593	0.616		0.573	0.584	0.561	0.573
**30**	0.592	0.618	0.602	0.604		0.623	0.641	0.597	0.620		0.576	0.586	0.566	0.576
**30.5**	0.595	0.62	0.606	0.607		0.624	0.642	0.6	0.622		0.578	0.589	0.569	0.579
**31**	0.599	0.623	0.609	0.610		0.629	0.646	0.604	0.626		0.581	0.592	0.573	0.582
**31.5**	0.601	0.625	0.613	0.613		0.63	0.648	0.606	0.628		0.584	0.596	0.576	0.585
**32**	0.606	0.627	0.616	0.616		0.633	0.651	0.611	0.632		0.587	0.599	0.58	0.589
**32.5**	0.608	0.627	0.62	0.618		0.633	0.651	0.613	0.632		0.588	0.601	0.583	0.591
**33**	0.612	0.628	0.622	0.621		0.636	0.652	0.616	0.635		0.59	0.603	0.585	0.593
**33.5**	0.614	0.631	0.626	0.624		0.639	0.656	0.618	0.638		0.592	0.606	0.587	0.595
**34**	0.617	0.634	0.629	0.627		0.64	0.657	0.621	0.639		0.595	0.607	0.59	0.597
**34.5**	0.621	0.638	0.631	0.630		0.642	0.66	0.624	0.642		0.598	0.611	0.593	0.601
**35**	0.623	0.639	0.634	0.632		0.644	0.661	0.627	0.644		0.601	0.614	0.595	0.603
**35.5**	0.624	0.642	0.638	0.635		0.646	0.663	0.63	0.646		0.603	0.615	0.597	0.605
**36**	0.626	0.646	0.64	0.637		0.647	0.665	0.632	0.648		0.605	0.617	0.599	0.607
**36.5**	0.628	0.649	0.643	0.640		0.649	0.666	0.635	0.650		0.608	0.619	0.603	0.610
**37**	0.631	0.652	0.646	0.643		0.653	0.67	0.637	0.653		0.61	0.622	0.604	0.612
**37.5**	0.632	0.654	0.649	0.645		0.653	0.67	0.64	0.654		0.612	0.624	0.608	0.615
**38**	0.634	0.655	0.651	0.647		0.654	0.671	0.643	0.656		0.614	0.626	0.61	0.617
**38.5**	0.636	0.658	0.653	0.649		0.657	0.674	0.645	0.659		0.617	0.627	0.612	0.619
**39**	0.637	0.661	0.656	0.651		0.657	0.675	0.647	0.660		0.619	0.63	0.615	0.621
**39.5**	0.639	0.663	0.658	0.653		0.66	0.678	0.649	0.662		0.623	0.632	0.618	0.624
**40**	0.641	0.666	0.661	0.656		0.661	0.679	0.652	0.664		0.624	0.634	0.619	0.626
**40.5**	0.642	0.666	0.663	0.657		0.662	0.68	0.652	0.665		0.626	0.635	0.622	0.628
**41**	0.643	0.667	0.664	0.658		0.664	0.68	0.655	0.666		0.629	0.638	0.625	0.631
**41.5**	0.644	0.668	0.666	0.659		0.665	0.683	0.657	0.668		0.632	0.639	0.626	0.632
**42**	0.647	0.67	0.669	0.662		0.667	0.683	0.659	0.670		0.633	0.641	0.627	0.634
**42.5**	0.647	0.67	0.671	0.663		0.667	0.684	0.661	0.671		0.636	0.642	0.63	0.636
**43**	0.648	0.671	0.671	0.663		0.669	0.685	0.662	0.672		0.637	0.643	0.63	0.637
**43.5**	0.65	0.672	0.674	0.665		0.67	0.685	0.664	0.673		0.639	0.645	0.634	0.639
**44**	0.651	0.672	0.676	0.666		0.671	0.686	0.668	0.675		0.641	0.647	0.634	0.641
**44.5**	0.653	0.672	0.678	0.668		0.673	0.687	0.668	0.676		0.643	0.649	0.637	0.643
**45**	0.654	0.67	0.68	0.668		0.673	0.687	0.67	0.677		0.645	0.65	0.638	0.644
**45.5**	0.655	0.669	0.68	0.668		0.672	0.686	0.671	0.676		0.647	0.652	0.64	0.646

**46**	0.657	0.668	0.681	0.669		0.674	0.687	0.672	0.678		0.648	0.654	0.641	0.648
**46.5**	0.657	0.666	0.682	0.668		0.675	0.685	0.673	0.678		0.65	0.656	0.642	0.649
**47**	0.657	0.662	0.683	0.667		0.676	0.686	0.674	0.679		0.652	0.657	0.644	0.651
**47.5**	0.659	0.659	0.685	0.668		0.676	0.687	0.676	0.680		0.654	0.658	0.646	0.653
**48**	0.66	0.653	0.687	0.667		0.676	0.686	0.676	0.679		0.654	0.659	0.646	0.653
**48.5**	0.661	0.65	0.687	0.666		0.677	0.687	0.679	0.681		0.657	0.66	0.649	0.655
**49**	0.663	0.648	0.689	0.667		0.678	0.686	0.678	0.681		0.658	0.661	0.649	0.656
**49.5**	0.664	0.65	0.69	0.668		0.679	0.687	0.68	0.682		0.661	0.663	0.651	0.658
**50**	0.665	0.65	0.69	0.668		0.679	0.686	0.681	0.682		0.661	0.664	0.651	0.659
**50.5**	0.666	0.651	0.69	0.669		0.679	0.685	0.68	0.681		0.663	0.665	0.653	0.660
**51**	0.666	0.652	0.692	0.670		0.679	0.685	0.684	0.683		0.664	0.666	0.654	0.661
**51.5**	0.667	0.652	0.692	0.670		0.68	0.686	0.681	0.682		0.665	0.667	0.655	0.662
**52**	0.669	0.653	0.692	0.671		0.682	0.685	0.684	0.684		0.666	0.668	0.657	0.664
**52.5**	0.669	0.654	0.692	0.672		0.682	0.686	0.684	0.684		0.666	0.669	0.657	0.664
**53**	0.671	0.654	0.694	0.673		0.683	0.687	0.684	0.685		0.667	0.67	0.659	0.665
**53.5**	0.671	0.655	0.694	0.673		0.685	0.686	0.686	0.686		0.668	0.671	0.66	0.666
**54**	0.672	0.656	0.695	0.674		0.684	0.685	0.685	0.685		0.667	0.671	0.66	0.666
**54.5**	0.673	0.656	0.696	0.675		0.687	0.688	0.688	0.688		0.668	0.673	0.661	0.667
**55**	0.673	0.658	0.695	0.675		0.687	0.688	0.686	0.687		0.669	0.674	0.663	0.669
**55.5**	0.675	0.657	0.698	0.677		0.688	0.688	0.688	0.688		0.669	0.674	0.663	0.669
**56**	0.675	0.657	0.699	0.677		0.688	0.688	0.69	0.689		0.67	0.675	0.665	0.670
**56.5**	0.674	0.658	0.699	0.677		0.689	0.689	0.69	0.689		0.67	0.676	0.666	0.671
**57**	0.677	0.659	0.7	0.679		0.69	0.691	0.692	0.691		0.671	0.677	0.668	0.672
**57.5**	0.676	0.659	0.7	0.678		0.69	0.69	0.691	0.690		0.671	0.678	0.669	0.673
**58**	0.676	0.659	0.701	0.679		0.691	0.691	0.692	0.691		0.671	0.678	0.668	0.672
**58.5**	0.677	0.659	0.703	0.680		0.691	0.69	0.692	0.691		0.672	0.679	0.668	0.673
**59**	0.678	0.661	0.705	0.681		0.693	0.691	0.694	0.693		0.673	0.68	0.671	0.675
**59.5**	0.679	0.661	0.706	0.682		0.694	0.693	0.695	0.694		0.674	0.68	0.672	0.675
**60**	0.68	0.662	0.707	0.683		0.695	0.693	0.696	0.695		0.675	0.681	0.672	0.676
**60.5**	0.68	0.662	0.709	0.684		0.694	0.695	0.698	0.696		0.676	0.681	0.673	0.677
**61**	0.681	0.661	0.71	0.684		0.696	0.695	0.698	0.696		0.676	0.682	0.674	0.677
**61.5**	0.681	0.663	0.711	0.685		0.695	0.696	0.698	0.696		0.677	0.683	0.675	0.678
**62**	0.682	0.664	0.711	0.686		0.696	0.697	0.7	0.698		0.678	0.684	0.677	0.680
**62.5**	0.682	0.664	0.712	0.686		0.697	0.697	0.699	0.698		0.677	0.684	0.676	0.679
**63**	0.683	0.665	0.714	0.687		0.697	0.698	0.702	0.699		0.679	0.684	0.678	0.680
**63.5**	0.683	0.666	0.713	0.687		0.699	0.699	0.7	0.699		0.68	0.685	0.679	0.681
**64**	0.684	0.667	0.715	0.689		0.698	0.699	0.703	0.700		0.681	0.686	0.68	0.682
**64.5**	0.685	0.668	0.715	0.689		0.698	0.699	0.704	0.700		0.682	0.687	0.681	0.683
**65**	0.685	0.67	0.716	0.690		0.699	0.7	0.704	0.701		0.683	0.688	0.682	0.684
**65.5**	0.685	0.67	0.716	0.690		0.701	0.699	0.706	0.702		0.683	0.687	0.682	0.684
**66**	0.685	0.672	0.717	0.691		0.7	0.701	0.707	0.703		0.685	0.689	0.684	0.686
**66.5**	0.686	0.673	0.718	0.692		0.7	0.701	0.707	0.703		0.685	0.689	0.684	0.686
**67**	0.686	0.677	0.718	0.694		0.702	0.702	0.708	0.704		0.686	0.69	0.685	0.687
**67.5**	0.687	0.678	0.72	0.695		0.701	0.703	0.707	0.704		0.687	0.69	0.687	0.688
**68**	0.687	0.679	0.72	0.695		0.702	0.703	0.709	0.705		0.688	0.69	0.686	0.688
**68.5**	0.688	0.678	0.721	0.696		0.702	0.704	0.71	0.705		0.689	0.692	0.688	0.690
**69**	0.688	0.688	0.721	0.699		0.702	0.703	0.71	0.705		0.69	0.692	0.688	0.690
**69.5**	0.688	0.691	0.722	0.700		0.702	0.703	0.711	0.705		0.69	0.693	0.689	0.691
**70**	0.688	0.691	0.721	0.700		0.703	0.703	0.711	0.706		0.692	0.694	0.689	0.692
**70.5**	0.69	0.688	0.723	0.700		0.703	0.702	0.713	0.706		0.692	0.695	0.691	0.693
**71**	0.689	0.686	0.722	0.699		0.704	0.702	0.713	0.706		0.693	0.695	0.691	0.693
**71.5**	0.689	0.686	0.722	0.699		0.704	0.701	0.714	0.706		0.694	0.695	0.693	0.694

**72**	0.689	0.683	0.722	0.698		0.704	0.701	0.714	0.706		0.694	0.697	0.693	0.695
**72.5**	0.69	0.677	0.723	0.697		0.705	0.701	0.714	0.707		0.695	0.697	0.694	0.695

Supplementary Table 5C2. Raw growth data measured for the deletion strain (Δ*s479*) at standard zinc concentration. For each time point, OD_{650nm} of three biological (replicate 1-3; s479-1 to s479-3), with three technical replicates (A to C) each, are given. Additionally, the mean OD_{650nm} over all technical replicates is given for each biological replicate.

	**replicate 1**					**replicate 2**					**replicate 3**			
	**s479-1A**	**s479-1B**	**s479-1C**	**s479-1**		**s479-2A**	**s479-2B**	**s479-2C**	**s479-2**		**s479-3A**	**s479-3B**	**s479-3C**	**s479-3**
**time [h]**	OD650	OD650	OD650	**Mean OD650**		OD650	OD650	OD650	**Mean OD650**		OD650	OD650	OD650	**Mean OD650**
**0.5**	0.064	0.065	0.065	0.065		0.056	0.054	0.056	0.055		0.067	0.063	0.062	0.064
**1**	0.066	0.065	0.065	0.065		0.061	0.059	0.061	0.060		0.074	0.071	0.069	0.071
**1.5**	0.08	0.08	0.08	0.080		0.069	0.067	0.068	0.068		0.085	0.082	0.079	0.082
**2**	0.091	0.092	0.091	0.091		0.077	0.074	0.077	0.076		0.095	0.091	0.089	0.092
**2.5**	0.1	0.101	0.101	0.101		0.087	0.084	0.087	0.086		0.103	0.1	0.098	0.100
**3**	0.109	0.111	0.11	0.110		0.095	0.092	0.095	0.094		0.111	0.109	0.106	0.109
**3.5**	0.119	0.12	0.121	0.120		0.102	0.099	0.103	0.101		0.12	0.118	0.115	0.118
**4**	0.13	0.132	0.133	0.132		0.11	0.107	0.111	0.109		0.13	0.127	0.124	0.127
**4.5**	0.141	0.143	0.143	0.142		0.12	0.117	0.122	0.120		0.142	0.139	0.132	0.138
**5**	0.154	0.155	0.155	0.155		0.13	0.128	0.131	0.130		0.151	0.148	0.144	0.148
**5.5**	0.167	0.167	0.167	0.167		0.14	0.139	0.142	0.140		0.164	0.16	0.158	0.161
**6**	0.178	0.18	0.181	0.180		0.151	0.148	0.155	0.151		0.178	0.173	0.171	0.174
**6.5**	0.191	0.192	0.193	0.192		0.161	0.16	0.166	0.162		0.19	0.186	0.185	0.187
**7**	0.204	0.206	0.205	0.205		0.173	0.171	0.179	0.174		0.202	0.203	0.198	0.201
**7.5**	0.218	0.22	0.221	0.220		0.186	0.183	0.191	0.187		0.215	0.216	0.213	0.215
**8**	0.231	0.234	0.237	0.234		0.197	0.195	0.203	0.198		0.228	0.228	0.228	0.228
**8.5**	0.248	0.25	0.255	0.251		0.208	0.207	0.215	0.210		0.242	0.245	0.242	0.243
**9**	0.26	0.264	0.271	0.265		0.219	0.219	0.229	0.222		0.255	0.259	0.255	0.256
**9.5**	0.273	0.278	0.289	0.280		0.231	0.231	0.242	0.235		0.27	0.273	0.268	0.270
**10**	0.286	0.289	0.303	0.293		0.242	0.244	0.254	0.247		0.282	0.283	0.281	0.282
**10.5**	0.299	0.302	0.317	0.306		0.255	0.257	0.267	0.260		0.294	0.297	0.293	0.295
**11**	0.31	0.312	0.327	0.316		0.267	0.267	0.279	0.271		0.307	0.308	0.306	0.307
**11.5**	0.323	0.326	0.341	0.330		0.278	0.28	0.29	0.283		0.319	0.317	0.315	0.317
**12**	0.335	0.338	0.353	0.342		0.289	0.292	0.301	0.294		0.329	0.328	0.327	0.328
**12.5**	0.345	0.348	0.362	0.352		0.3	0.302	0.312	0.305		0.339	0.336	0.335	0.337
**13**	0.355	0.358	0.371	0.361		0.311	0.312	0.322	0.315		0.349	0.345	0.346	0.347
**13.5**	0.365	0.367	0.38	0.371		0.321	0.322	0.331	0.325		0.359	0.353	0.353	0.355
**14**	0.374	0.375	0.387	0.379		0.329	0.33	0.339	0.333		0.367	0.36	0.361	0.363
**14.5**	0.381	0.383	0.396	0.387		0.337	0.338	0.347	0.341		0.377	0.368	0.369	0.371
**15**	0.39	0.391	0.404	0.395		0.347	0.347	0.356	0.350		0.386	0.376	0.376	0.379
**15.5**	0.397	0.398	0.409	0.401		0.354	0.355	0.363	0.357		0.392	0.383	0.385	0.387
**16**	0.403	0.402	0.415	0.407		0.362	0.363	0.372	0.366		0.4	0.39	0.392	0.394
**16.5**	0.409	0.409	0.421	0.413		0.37	0.371	0.379	0.373		0.406	0.397	0.401	0.401
**17**	0.416	0.417	0.428	0.420		0.378	0.377	0.387	0.381		0.414	0.405	0.409	0.409
**17.5**	0.419	0.423	0.432	0.425		0.385	0.385	0.394	0.388		0.421	0.411	0.415	0.416
**18**	0.428	0.427	0.439	0.431		0.392	0.393	0.399	0.395		0.428	0.419	0.423	0.423
**18.5**	0.433	0.434	0.443	0.437		0.398	0.399	0.406	0.401		0.433	0.424	0.431	0.429
**19**	0.437	0.439	0.448	0.441		0.405	0.406	0.413	0.408		0.44	0.432	0.439	0.437
**19.5**	0.442	0.444	0.455	0.447		0.411	0.412	0.419	0.414		0.447	0.439	0.447	0.444
**20**	0.448	0.448	0.459	0.452		0.417	0.417	0.424	0.419		0.453	0.445	0.453	0.450
**20.5**	0.454	0.455	0.463	0.457		0.423	0.424	0.43	0.426		0.459	0.451	0.46	0.457

**21**	0.458	0.459	0.468	0.462		0.429	0.429	0.435	0.431		0.464	0.459	0.467	0.463
**21.5**	0.463	0.465	0.474	0.467		0.434	0.435	0.441	0.437		0.47	0.463	0.473	0.469
**22**	0.469	0.472	0.48	0.474		0.44	0.442	0.447	0.443		0.476	0.471	0.48	0.476
**22.5**	0.474	0.476	0.483	0.478		0.446	0.446	0.451	0.448		0.481	0.476	0.485	0.481
**23**	0.477	0.48	0.488	0.482		0.451	0.451	0.457	0.453		0.486	0.481	0.491	0.486
**23.5**	0.482	0.485	0.493	0.487		0.455	0.456	0.462	0.458		0.491	0.486	0.497	0.491
**24**	0.486	0.489	0.496	0.490		0.46	0.461	0.467	0.463		0.495	0.491	0.502	0.496
**24.5**	0.491	0.493	0.5	0.495		0.466	0.466	0.472	0.468		0.499	0.496	0.508	0.501
**25**	0.495	0.498	0.504	0.499		0.47	0.47	0.476	0.472		0.503	0.501	0.513	0.506
**25.5**	0.499	0.502	0.507	0.503		0.474	0.474	0.48	0.476		0.508	0.506	0.518	0.511
**26**	0.502	0.505	0.511	0.506		0.477	0.476	0.484	0.479		0.512	0.51	0.523	0.515
**26.5**	0.507	0.509	0.515	0.510		0.482	0.482	0.489	0.484		0.514	0.515	0.528	0.519
**27**	0.511	0.512	0.519	0.514		0.485	0.485	0.492	0.487		0.519	0.519	0.532	0.523
**27.5**	0.514	0.516	0.521	0.517		0.489	0.488	0.495	0.491		0.521	0.523	0.536	0.527
**28**	0.517	0.518	0.523	0.519		0.492	0.492	0.497	0.494		0.525	0.526	0.54	0.530
**28.5**	0.52	0.521	0.526	0.522		0.497	0.496	0.501	0.498		0.53	0.53	0.545	0.535
**29**	0.523	0.524	0.528	0.525		0.5	0.498	0.504	0.501		0.533	0.533	0.548	0.538
**29.5**	0.526	0.527	0.531	0.528		0.503	0.502	0.507	0.504		0.536	0.537	0.552	0.542
**30**	0.529	0.529	0.532	0.530		0.508	0.506	0.51	0.508		0.54	0.539	0.556	0.545
**30.5**	0.533	0.533	0.535	0.534		0.511	0.51	0.513	0.511		0.543	0.543	0.56	0.549
**31**	0.536	0.535	0.538	0.536		0.514	0.513	0.515	0.514		0.546	0.546	0.562	0.551
**31.5**	0.538	0.537	0.54	0.538		0.517	0.516	0.518	0.517		0.548	0.549	0.567	0.555
**32**	0.541	0.541	0.543	0.542		0.521	0.519	0.522	0.521		0.552	0.554	0.57	0.559
**32.5**	0.543	0.542	0.543	0.543		0.524	0.522	0.524	0.523		0.555	0.554	0.573	0.561
**33**	0.546	0.545	0.546	0.546		0.526	0.525	0.525	0.525		0.556	0.557	0.575	0.563
**33.5**	0.548	0.547	0.548	0.548		0.529	0.527	0.528	0.528		0.559	0.56	0.579	0.566
**34**	0.55	0.55	0.55	0.550		0.533	0.53	0.531	0.531		0.561	0.563	0.581	0.568
**34.5**	0.553	0.552	0.553	0.553		0.536	0.533	0.534	0.534		0.564	0.566	0.585	0.572
**35**	0.556	0.555	0.555	0.555		0.538	0.535	0.535	0.536		0.567	0.568	0.588	0.574
**35.5**	0.557	0.555	0.557	0.556		0.541	0.538	0.538	0.539		0.569	0.571	0.592	0.577
**36**	0.558	0.558	0.559	0.558		0.544	0.539	0.539	0.541		0.572	0.574	0.594	0.580
**36.5**	0.562	0.561	0.56	0.561		0.546	0.542	0.542	0.543		0.573	0.577	0.598	0.583
**37**	0.564	0.563	0.562	0.563		0.548	0.544	0.544	0.545		0.576	0.58	0.601	0.586
**37.5**	0.566	0.565	0.564	0.565		0.55	0.546	0.546	0.547		0.577	0.582	0.602	0.587
**38**	0.568	0.567	0.566	0.567		0.553	0.548	0.548	0.550		0.582	0.584	0.606	0.591
**38.5**	0.57	0.567	0.566	0.568		0.555	0.551	0.552	0.553		0.581	0.586	0.61	0.592
**39**	0.572	0.57	0.569	0.570		0.557	0.553	0.553	0.554		0.584	0.588	0.613	0.595
**39.5**	0.575	0.573	0.572	0.573		0.559	0.555	0.555	0.556		0.587	0.59	0.616	0.598
**40**	0.577	0.575	0.572	0.575		0.561	0.557	0.557	0.558		0.588	0.594	0.619	0.600
**40.5**	0.579	0.576	0.575	0.577		0.563	0.559	0.56	0.561		0.589	0.595	0.623	0.602
**41**	0.579	0.576	0.576	0.577		0.564	0.561	0.562	0.562		0.593	0.596	0.629	0.606
**41.5**	0.583	0.58	0.577	0.580		0.565	0.563	0.565	0.564		0.592	0.602	0.635	0.610
**42**	0.585	0.581	0.58	0.582		0.567	0.564	0.567	0.566		0.596	0.601	0.625	0.607
**42.5**	0.588	0.583	0.581	0.584		0.569	0.566	0.569	0.568		0.597	0.603	0.629	0.610
**43**	0.589	0.583	0.583	0.585		0.571	0.569	0.571	0.570		0.599	0.608	0.636	0.614
**43.5**	0.591	0.586	0.582	0.586		0.573	0.569	0.572	0.571		0.601	0.609	0.641	0.617
**44**	0.592	0.588	0.584	0.588		0.575	0.572	0.574	0.574		0.603	0.609	0.646	0.619
**44.5**	0.595	0.589	0.587	0.590		0.576	0.574	0.577	0.576		0.605	0.613	0.654	0.624
**45**	0.596	0.591	0.588	0.592		0.578	0.575	0.577	0.577		0.607	0.613	0.661	0.627
**45.5**	0.599	0.593	0.591	0.594		0.58	0.577	0.579	0.579		0.607	0.614	0.668	0.630
**46**	0.601	0.595	0.593	0.596		0.581	0.579	0.581	0.580		0.607	0.616	0.674	0.632

**46.5**	0.602	0.596	0.592	0.597		0.584	0.58	0.583	0.582		0.608	0.619	0.677	0.635
**47**	0.604	0.597	0.595	0.599		0.586	0.582	0.585	0.584		0.61	0.619	0.679	0.636
**47.5**	0.605	0.598	0.596	0.600		0.587	0.585	0.587	0.586		0.611	0.621	0.68	0.637
**48**	0.607	0.601	0.598	0.602		0.589	0.586	0.588	0.588		0.611	0.621	0.681	0.638
**48.5**	0.607	0.601	0.599	0.602		0.59	0.588	0.591	0.590		0.613	0.62	0.684	0.639
**49**	0.609	0.603	0.601	0.604		0.593	0.591	0.592	0.592		0.615	0.622	0.685	0.641
**49.5**	0.611	0.605	0.602	0.606		0.595	0.592	0.594	0.594		0.617	0.621	0.685	0.641
**50**	0.612	0.606	0.603	0.607		0.596	0.594	0.594	0.595		0.618	0.624	0.683	0.642
**50.5**	0.613	0.608	0.604	0.608		0.598	0.596	0.596	0.597		0.619	0.624	0.678	0.640
**51**	0.613	0.607	0.606	0.609		0.6	0.597	0.596	0.598		0.62	0.626	0.673	0.640
**51.5**	0.615	0.609	0.607	0.610		0.601	0.598	0.597	0.599		0.622	0.625	0.666	0.638
**52**	0.616	0.61	0.608	0.611		0.603	0.599	0.598	0.600		0.621	0.627	0.662	0.637
**52.5**	0.617	0.611	0.608	0.612		0.604	0.6	0.599	0.601		0.624	0.626	0.663	0.638
**53**	0.618	0.612	0.61	0.613		0.605	0.601	0.599	0.602		0.624	0.627	0.664	0.638
**53.5**	0.618	0.612	0.61	0.613		0.606	0.602	0.601	0.603		0.625	0.627	0.668	0.640
**54**	0.618	0.612	0.61	0.613		0.607	0.603	0.6	0.603		0.626	0.628	0.668	0.641
**54.5**	0.62	0.614	0.611	0.615		0.607	0.604	0.602	0.604		0.627	0.63	0.668	0.642
**55**	0.62	0.614	0.612	0.615		0.608	0.605	0.603	0.605		0.627	0.629	0.669	0.642
**55.5**	0.62	0.615	0.612	0.616		0.609	0.606	0.603	0.606		0.628	0.63	0.674	0.644
**56**	0.622	0.615	0.613	0.617		0.609	0.607	0.603	0.606		0.63	0.63	0.678	0.646
**56.5**	0.622	0.616	0.613	0.617		0.61	0.607	0.604	0.607		0.63	0.631	0.682	0.648
**57**	0.622	0.616	0.615	0.618		0.612	0.608	0.605	0.608		0.631	0.632	0.685	0.649
**57.5**	0.621	0.616	0.614	0.617		0.612	0.609	0.606	0.609		0.632	0.632	0.689	0.651
**58**	0.622	0.617	0.615	0.618		0.612	0.609	0.607	0.609		0.632	0.634	0.693	0.653
**58.5**	0.623	0.618	0.615	0.619		0.613	0.609	0.608	0.610		0.634	0.632	0.696	0.654
**59**	0.623	0.617	0.616	0.619		0.613	0.61	0.609	0.611		0.634	0.634	0.698	0.655
**59.5**	0.622	0.618	0.616	0.619		0.613	0.611	0.61	0.611		0.635	0.635	0.701	0.657
**60**	0.622	0.619	0.616	0.619		0.614	0.612	0.61	0.612		0.636	0.637	0.704	0.659
**60.5**	0.622	0.619	0.617	0.619		0.614	0.613	0.611	0.613		0.636	0.638	0.702	0.659
**61**	0.622	0.619	0.617	0.619		0.615	0.613	0.611	0.613		0.637	0.64	0.688	0.655
**61.5**	0.623	0.62	0.617	0.620		0.615	0.613	0.611	0.613		0.638	0.64	0.705	0.661
**62**	0.623	0.62	0.617	0.620		0.615	0.614	0.612	0.614		0.638	0.64	0.714	0.664
**62.5**	0.624	0.621	0.619	0.621		0.615	0.613	0.613	0.614		0.639	0.64	0.72	0.666
**63**	0.624	0.621	0.619	0.621		0.616	0.614	0.613	0.614		0.64	0.64	0.725	0.668
**63.5**	0.624	0.622	0.619	0.622		0.616	0.614	0.613	0.614		0.641	0.642	0.725	0.669
**64**	0.624	0.623	0.62	0.622		0.615	0.615	0.614	0.615		0.641	0.643	0.724	0.669
**64.5**	0.625	0.623	0.62	0.623		0.616	0.616	0.614	0.615		0.641	0.643	0.725	0.670
**65**	0.625	0.624	0.62	0.623		0.617	0.616	0.615	0.616		0.643	0.645	0.726	0.671
**65.5**	0.625	0.623	0.621	0.623		0.617	0.616	0.615	0.616		0.643	0.646	0.725	0.671
**66**	0.625	0.624	0.621	0.623		0.618	0.617	0.616	0.617		0.643	0.645	0.725	0.671
**66.5**	0.626	0.624	0.62	0.623		0.618	0.617	0.616	0.617		0.644	0.647	0.724	0.672
**67**	0.626	0.625	0.622	0.624		0.619	0.617	0.616	0.617		0.645	0.649	0.722	0.672
**67.5**	0.626	0.624	0.622	0.624		0.619	0.618	0.617	0.618		0.644	0.648	0.72	0.671
**68**	0.626	0.625	0.621	0.624		0.62	0.619	0.617	0.619		0.645	0.647	0.72	0.671
**68.5**	0.627	0.625	0.623	0.625		0.62	0.619	0.618	0.619		0.645	0.649	0.717	0.670
**69**	0.627	0.626	0.622	0.625		0.62	0.619	0.617	0.619		0.646	0.648	0.715	0.670
**69.5**	0.627	0.626	0.623	0.625		0.621	0.62	0.618	0.620		0.646	0.646	0.714	0.669
**70**	0.628	0.626	0.623	0.626		0.622	0.62	0.619	0.620		0.646	0.645	0.713	0.668
**70.5**	0.628	0.627	0.623	0.626		0.622	0.621	0.618	0.620		0.645	0.645	0.713	0.668
**71**	0.628	0.627	0.623	0.626		0.622	0.621	0.619	0.621		0.647	0.646	0.712	0.668
**71.5**	0.628	0.627	0.623	0.626		0.623	0.622	0.619	0.621		0.647	0.643	0.712	0.667
**72**	0.628	0.627	0.622	0.626		0.624	0.622	0.62	0.622		0.647	0.644	0.711	0.667

**72.5**	0.628	0.627	0.624	0.626		0.624	0.621	0.619	0.621		0.647	0.644	0.711	0.667

Supplementary Table 6. Data and calculations for growth experiments at high zinc concentration. Supplementary Table 6A1. and 6A2. list the data used to compile the growth curves in Supplementary Figure 5A. Supplementary Table 6B. shows the calculations of growth rate and doubling time for wildtype and deletion strain (Δ*s479*). Supplementary Table 6C1. and 6C2. list all the raw data collected for the growth experiments at high zinc concentration. For better readability, all numbers were cut to three decimals or less. **Supplementary Table 6A1. Mean growth and standard deviation used to compile the wildtype strain (H66) growth curve in Supplementary Figure 5A.** The mean OD_{650nm} is given for all three biological replicates of wildtype strain H66 (H66-1 to H66-3) alongside the overall mean OD_{650nm} and standard deviation per time interval.

H66-1	H66-2	H66-3	H66	H66	
**Mean OD650**	**Mean OD650**	**Mean OD650**	**Mean OD650**	**SD**	**time [h]**
0.066	0.064	0.062	**0.064**	0.002	**0.5**
0.090	0.093	0.093	**0.092**	0.001	**2.5**
0.122	0.129	0.131	**0.127**	0.004	**4.5**
0.151	0.165	0.171	**0.162**	0.008	**6.5**
0.172	0.200	0.213	**0.195**	0.017	**8.5**
0.196	0.239	0.257	**0.231**	0.026	**10.5**
0.220	0.285	0.299	**0.268**	0.035	**12.5**
0.245	0.339	0.347	**0.310**	0.046	**14.5**
0.274	0.393	0.394	**0.354**	0.056	**16.5**
0.308	0.444	0.447	**0.400**	0.065	**18.5**
0.357	0.496	0.495	**0.449**	0.065	**20.5**
0.423	0.538	0.537	**0.499**	0.054	**22.5**
0.489	0.573	0.573	**0.545**	0.039	**24.5**
0.550	0.601	0.604	**0.585**	0.025	**26.5**
0.599	0.626	0.629	**0.618**	0.014	**28.5**
0.637	0.648	0.651	**0.645**	0.006	**30.5**
0.670	0.668	0.672	**0.670**	0.001	**32.5**
0.697	0.686	0.691	**0.692**	0.005	**34.5**
0.721	0.704	0.710	**0.712**	0.007	**36.5**
0.742	0.717	0.728	**0.729**	0.010	**38.5**
0.760	0.730	0.741	**0.744**	0.013	**40.5**
0.775	0.741	0.754	**0.757**	0.014	**42.5**
0.784	0.746	0.763	**0.765**	0.016	**44.5**
0.791	0.753	0.771	**0.771**	0.016	**46.5**
0.798	0.758	0.775	**0.777**	0.017	**48.5**
0.802	0.763	0.781	**0.782**	0.016	**50.5**
0.806	0.768	0.788	**0.787**	0.015	**52.5**
0.808	0.775	0.794	**0.793**	0.014	**54.5**
0.814	0.781	0.800	**0.798**	0.013	**56.5**
0.815	0.786	0.805	**0.802**	0.012	**58.5**
0.820	0.792	0.812	**0.808**	0.012	**60.5**
0.823	0.796	0.817	**0.812**	0.012	**62.5**
0.827	0.802	0.822	**0.817**	0.011	**64.5**
0.831	0.807	0.827	**0.822**	0.011	**66.5**
0.834	0.812	0.832	**0.826**	0.010	**68.5**
0.839	0.817	0.837	**0.831**	0.010	**70.5**

	0.821	0.842	**0.831**	0.010	**72.5**

Supplementary Table 6A2. Mean growth and standard deviation used to compile the deletion strain (Δ*s479*) growth curve in Supplementary Figure 5A. The mean OD_{650nm} is given for all three biological replicates of deletion strain Δ*s479* (s479-1 to s479-3) alongside the overall mean OD_{650nm} and standard deviation per time interval.

s479-1	s479-2	s479-3	s479	s479 SD	
Mean OD650	Mean OD650	Mean OD650	Mean OD650	SD	time [h]
0.066	0.063	0.061	**0.063**	0.002	**0.5**
0.078	0.075	0.075	**0.076**	0.001	**2.5**
0.095	0.094	0.091	**0.093**	0.002	**4.5**
0.112	0.116	0.107	**0.112**	0.003	**6.5**
0.129	0.136	0.125	**0.130**	0.004	**8.5**
0.143	0.156	0.143	**0.147**	0.006	**10.5**
0.162	0.176	0.162	**0.167**	0.007	**12.5**
0.183	0.197	0.183	**0.188**	0.007	**14.5**
0.205	0.223	0.204	**0.210**	0.009	**16.5**
0.227	0.247	0.226	**0.233**	0.010	**18.5**
0.252	0.273	0.250	**0.259**	0.010	**20.5**
0.281	0.297	0.272	**0.283**	0.010	**22.5**
0.308	0.322	0.295	**0.308**	0.011	**24.5**
0.353	0.348	0.319	**0.340**	0.015	**26.5**
0.402	0.375	0.345	**0.374**	0.023	**28.5**
0.445	0.405	0.378	**0.409**	0.027	**30.5**
0.489	0.435	0.412	**0.446**	0.032	**32.5**
0.527	0.464	0.443	**0.478**	0.036	**34.5**
0.561	0.495	0.474	**0.510**	0.037	**36.5**
0.589	0.523	0.499	**0.537**	0.038	**38.5**
0.609	0.546	0.522	**0.559**	0.037	**40.5**
0.631	0.560	0.543	**0.578**	0.038	**42.5**
0.647	0.577	0.563	**0.596**	0.037	**44.5**
0.673	0.590	0.582	**0.615**	0.041	**46.5**
0.688	0.603	0.598	**0.630**	0.042	**48.5**
0.693	0.610	0.609	**0.637**	0.040	**50.5**
0.703	0.621	0.621	**0.648**	0.039	**52.5**
0.708	0.630	0.630	**0.656**	0.037	**54.5**
0.718	0.640	0.641	**0.666**	0.036	**56.5**
0.722	0.648	0.649	**0.673**	0.034	**58.5**
0.728	0.655	0.658	**0.681**	0.034	**60.5**
0.730	0.661	0.669	**0.687**	0.031	**62.5**
0.738	0.667	0.673	**0.693**	0.032	**64.5**
0.741	0.673	0.679	**0.698**	0.031	**66.5**
0.745	0.680	0.687	**0.704**	0.029	**68.5**
0.750	0.682	0.694	**0.709**	0.029	**70.5**
0.758	0.688	0.702	**0.716**	0.030	**72.5**

Supplementary Table 6B. Calculation of growth rate and doubling time at high zinc concentration. Growth rate and doubling time were calculated as growth rate μ = (ln(xt) – ln (x0)) / (t – t0) and doubling time d= ln(2) / μ. Calculations were carried out separately for all replicates before calculating mean value (red) and standard deviation (red). Phases of exponential growth were identified using fitted trendlines and corresponding R^2-values (Supplementary Figure 5B.). Values are given separately for H66 (grey) and Δ*s479* (orange) and for each of the two phases.

Phase 1: 0.5 h-8.5 h

growth rate μ(H66) [h^{-1}] time interval 0.5 h to 8.5 h with R² =

H66-1	H66-2	H66-3	μ(H66) [h^{-1}]	SD(μ(H66)) [h^{-1}]
0.121	0.142	0.155	0.139	0.014

doubling time / generation time d(H66) [h]

H66-1	H66-2	H66-3	d(H66) [h]	SD(d(H66)) [h]
5.7	4.9	4.5	5.0	0.5

growth rate μ(s479) [h^{-1}] time interval 0.5 h to 8.5 h with R² =

s479-1	s479-2	s479-3	μ(s479) [h^{-1}]	SD(μ(s479)) [h^{-1}]
0.084	0.097	0.089	0.090	0.005

doubling time / generation time d(H66) [h]

s479-1	s479-2	s479-3	d(s479) [h]	SD(d(s479)) [h]
8.2	7.2	7.8	7.7	0.4

Phase2: 10.5 h-24.5 h

growth rate μ(H66) [h^{-1}] time interval 10.5 h to 24.5 h with R² = 0.9936

H66-1	H66-2	H66-3	μ(H66) [h^{-1}]	SD(μ(H66)) [h^{-1}]
0.065	0.062	0.057	0.062	0.003

doubling time / generation time d(H66) [h]

H66-1	H66-2	H66-3	d(H66) [h]	SD(d(H66)) [h]
10.6	11.1	12.1	11.3	0.6

growth rate μ(s479) [h^{-1}] time interval 10.5 h to 24.5 h with R² = 0.9962

s479-1	s479-2	s479-3	μ(s479) [h^{-1}]	SD(μ(s479)) [h^{-1}]
0.055	0.052	0.051	0.053	0.001

doubling time / generation time d(H66) [h]

s479-1	s479-2	s479-3	d(s479) [h]	SD(d(s479)) [h]
12.7	13.3	13.5	13.2	0.3

Supplementary Table 6C1. Raw growth data measured for the wildtype strain (H66) at high zinc concentration. For each time point, OD_{650nm} of three biological (replicate 1-3; H66-1 to H66-3), with three technical replicates (A-C) each, are given. Additionally, the mean OD_{650nm} over all technical replicates is given for each biological replicate.

	**replicate 1**					**replicate 2**					**replicate 3**			
	**H66-1A**	**H66-1B**	**H66-1C**	**H66-1**		**H66-2A**	**H66-2B**	**H66-2C**	**H66-2**		**H66-3A**	**H66-3B**	**H66-3C**	**H66-3**
**time [h]**	OD650	OD650	OD650	**Mean OD650**		OD650	OD650	OD650	**Mean OD650**		OD650	OD650	OD650	**Mean OD650**
**0.5**	0.063	0.066	0.068	0.066		0.055	0.064	0.073	0.064		0.059	0.059	0.067	0.062
**1**	0.065	0.069	0.073	0.069		0.059	0.069	0.081	0.070		0.065	0.064	0.073	0.067
**1.5**	0.07	0.075	0.078	0.074		0.065	0.077	0.088	0.077		0.073	0.071	0.081	0.075
**2**	0.077	0.083	0.086	0.082		0.072	0.084	0.096	0.084		0.081	0.079	0.089	0.083
**2.5**	0.084	0.091	0.095	0.090		0.08	0.093	0.105	0.093		0.091	0.088	0.099	0.093
**3**	0.091	0.099	0.103	0.098		0.087	0.102	0.115	0.101		0.1	0.097	0.108	0.102
**3.5**	0.098	0.106	0.111	0.105		0.096	0.113	0.124	0.111		0.109	0.106	0.117	0.111
**4**	0.105	0.115	0.12	0.113		0.104	0.122	0.133	0.120		0.12	0.115	0.127	0.121
**4.5**	0.113	0.124	0.13	0.122		0.113	0.131	0.143	0.129		0.13	0.126	0.136	0.131
**5**	0.121	0.133	0.138	0.131		0.122	0.141	0.151	0.138		0.142	0.136	0.145	0.141
**5.5**	0.129	0.141	0.147	0.139		0.131	0.151	0.158	0.147		0.154	0.146	0.154	0.151
**6**	0.135	0.148	0.153	0.145		0.14	0.161	0.166	0.156		0.166	0.156	0.162	0.161
**6.5**	0.141	0.155	0.158	0.151		0.151	0.169	0.174	0.165		0.177	0.167	0.169	0.171
**7**	0.147	0.161	0.164	0.157		0.16	0.178	0.18	0.173		0.19	0.178	0.177	0.182
**7.5**	0.153	0.167	0.17	0.163		0.17	0.189	0.187	0.182		0.202	0.189	0.185	0.192
**8**	0.159	0.173	0.174	0.169		0.182	0.197	0.194	0.191		0.217	0.202	0.192	0.204
**8.5**	0.164	0.176	0.177	0.172		0.193	0.206	0.2	0.200		0.23	0.213	0.197	0.213
**9**	0.171	0.183	0.183	0.179		0.205	0.216	0.208	0.210		0.243	0.224	0.204	0.224
**9.5**	0.174	0.191	0.188	0.184		0.213	0.226	0.213	0.217		0.256	0.236	0.21	0.234
**10**	0.182	0.196	0.193	0.190		0.225	0.237	0.221	0.228		0.269	0.25	0.218	0.246
**10.5**	0.188	0.203	0.198	0.196		0.239	0.249	0.229	0.239		0.283	0.263	0.226	0.257
**11**	0.192	0.208	0.205	0.202		0.249	0.26	0.239	0.249		0.294	0.276	0.229	0.266
**11.5**	0.198	0.214	0.21	0.207		0.26	0.273	0.248	0.260		0.305	0.288	0.238	0.277
**12**	0.206	0.221	0.217	0.215		0.273	0.285	0.258	0.272		0.317	0.302	0.245	0.288
**12.5**	0.21	0.228	0.222	0.220		0.285	0.301	0.269	0.285		0.327	0.317	0.254	0.299
**13**	0.215	0.235	0.228	0.226		0.297	0.314	0.279	0.297		0.337	0.33	0.262	0.310
**13.5**	0.222	0.241	0.233	0.232		0.311	0.328	0.292	0.310		0.35	0.343	0.273	0.322
**14**	0.227	0.249	0.24	0.239		0.325	0.342	0.307	0.325		0.362	0.358	0.285	0.335
**14.5**	0.235	0.255	0.245	0.245		0.339	0.354	0.324	0.339		0.372	0.37	0.299	0.347
**15**	0.243	0.264	0.254	0.254		0.349	0.368	0.338	0.352		0.38	0.383	0.313	0.359
**15.5**	0.25	0.271	0.259	0.260		0.362	0.381	0.354	0.366		0.388	0.395	0.328	0.370
**16**	0.259	0.279	0.265	0.268		0.372	0.394	0.37	0.379		0.398	0.408	0.343	0.383
**16.5**	0.266	0.285	0.27	0.274		0.385	0.406	0.387	0.393		0.405	0.418	0.36	0.394
**17**	0.274	0.294	0.277	0.282		0.396	0.42	0.406	0.407		0.417	0.432	0.378	0.409
**17.5**	0.282	0.303	0.284	0.290		0.406	0.433	0.423	0.421		0.427	0.443	0.393	0.421
**18**	0.29	0.313	0.291	0.298		0.421	0.446	0.438	0.435		0.435	0.456	0.406	0.432
**18.5**	0.3	0.325	0.3	0.308		0.426	0.456	0.451	0.444		0.445	0.468	0.428	0.447
**19**	0.311	0.336	0.31	0.319		0.443	0.47	0.469	0.461		0.455	0.48	0.441	0.459
**19.5**	0.322	0.349	0.321	0.331		0.45	0.479	0.482	0.470		0.466	0.492	0.459	0.472
**20**	0.334	0.364	0.333	0.344		0.465	0.493	0.495	0.484		0.474	0.504	0.473	0.484

**20.5**	0.347	0.377	0.347	0.357		0.477	0.502	0.509	0.496		0.485	0.514	0.487	0.495
**21**	0.362	0.395	0.359	0.372		0.486	0.514	0.522	0.507		0.492	0.524	0.502	0.506
**21.5**	0.377	0.413	0.375	0.388		0.498	0.525	0.532	0.518		0.502	0.535	0.514	0.517
**22**	0.394	0.43	0.392	0.405		0.508	0.531	0.544	0.528		0.512	0.545	0.526	0.528
**22.5**	0.41	0.448	0.41	0.423		0.514	0.544	0.555	0.538		0.52	0.554	0.537	0.537
**23**	0.428	0.465	0.426	0.440		0.526	0.552	0.565	0.548		0.53	0.565	0.547	0.547
**23.5**	0.446	0.481	0.444	0.457		0.535	0.561	0.575	0.557		0.538	0.573	0.559	0.557
**24**	0.462	0.496	0.461	0.473		0.544	0.568	0.585	0.566		0.545	0.581	0.566	0.564
**24.5**	0.476	0.513	0.479	0.489		0.55	0.576	0.592	0.573		0.553	0.589	0.577	0.573
**25**	0.492	0.529	0.494	0.505		0.558	0.584	0.601	0.581		0.563	0.597	0.586	0.582
**25.5**	0.506	0.543	0.514	0.521		0.566	0.592	0.608	0.589		0.569	0.605	0.593	0.589
**26**	0.519	0.557	0.531	0.536		0.573	0.598	0.616	0.596		0.575	0.61	0.601	0.595
**26.5**	0.535	0.57	0.546	0.550		0.578	0.603	0.623	0.601		0.584	0.618	0.61	0.604
**27**	0.546	0.583	0.561	0.563		0.586	0.611	0.63	0.609		0.589	0.625	0.616	0.610
**27.5**	0.559	0.595	0.576	0.577		0.59	0.617	0.636	0.614		0.596	0.631	0.622	0.616
**28**	0.568	0.605	0.59	0.588		0.596	0.623	0.642	0.620		0.603	0.636	0.629	0.623
**28.5**	0.576	0.616	0.604	0.599		0.601	0.629	0.647	0.626		0.61	0.641	0.637	0.629
**29**	0.587	0.626	0.616	0.610		0.607	0.634	0.654	0.632		0.616	0.646	0.642	0.635
**29.5**	0.595	0.634	0.628	0.619		0.613	0.64	0.66	0.638		0.621	0.652	0.648	0.640
**30**	0.602	0.643	0.636	0.627		0.617	0.645	0.665	0.642		0.626	0.656	0.652	0.645
**30.5**	0.61	0.651	0.649	0.637		0.624	0.651	0.67	0.648		0.633	0.662	0.659	0.651
**31**	0.618	0.661	0.66	0.646		0.63	0.655	0.675	0.653		0.639	0.667	0.664	0.657
**31.5**	0.625	0.667	0.67	0.654		0.635	0.66	0.679	0.658		0.645	0.672	0.666	0.661
**32**	0.631	0.675	0.679	0.662		0.64	0.665	0.683	0.663		0.651	0.677	0.673	0.667
**32.5**	0.638	0.682	0.69	0.670		0.647	0.67	0.688	0.668		0.658	0.681	0.677	0.672
**33**	0.644	0.689	0.698	0.677		0.651	0.674	0.691	0.672		0.662	0.687	0.682	0.677
**33.5**	0.65	0.695	0.706	0.684		0.656	0.679	0.696	0.677		0.666	0.692	0.685	0.681
**34**	0.655	0.701	0.712	0.689		0.661	0.684	0.7	0.682		0.673	0.697	0.689	0.686
**34.5**	0.662	0.708	0.722	0.697		0.666	0.689	0.704	0.686		0.678	0.701	0.694	0.691
**35**	0.667	0.713	0.731	0.704		0.671	0.693	0.708	0.691		0.684	0.706	0.699	0.696
**35.5**	0.673	0.719	0.737	0.710		0.677	0.697	0.712	0.695		0.688	0.71	0.703	0.700
**36**	0.679	0.725	0.743	0.716		0.681	0.702	0.716	0.700		0.694	0.716	0.707	0.706
**36.5**	0.684	0.73	0.75	0.721		0.687	0.707	0.719	0.704		0.7	0.719	0.711	0.710
**37**	0.689	0.735	0.755	0.726		0.692	0.71	0.72	0.707		0.706	0.725	0.716	0.716
**37.5**	0.694	0.742	0.761	0.732		0.695	0.713	0.724	0.711		0.71	0.731	0.719	0.720
**38**	0.7	0.746	0.766	0.737		0.699	0.716	0.725	0.713		0.714	0.735	0.722	0.724
**38.5**	0.705	0.754	0.767	0.742		0.704	0.719	0.729	0.717		0.719	0.739	0.725	0.728
**39**	0.708	0.756	0.774	0.746		0.706	0.721	0.731	0.719		0.722	0.743	0.727	0.731
**39.5**	0.712	0.761	0.778	0.750		0.707	0.726	0.734	0.722		0.725	0.747	0.73	0.734
**40**	0.719	0.767	0.781	0.756		0.712	0.729	0.738	0.726		0.728	0.752	0.733	0.738
**40.5**	0.724	0.771	0.786	0.760		0.717	0.732	0.741	0.730		0.731	0.754	0.737	0.741
**41**	0.729	0.776	0.788	0.764		0.72	0.736	0.745	0.734		0.733	0.758	0.738	0.743
**41.5**	0.733	0.78	0.792	0.768		0.723	0.737	0.747	0.736		0.737	0.761	0.742	0.747
**42**	0.736	0.783	0.795	0.771		0.725	0.741	0.749	0.738		0.74	0.763	0.743	0.749
**42.5**	0.739	0.788	0.799	0.775		0.727	0.744	0.752	0.741		0.744	0.768	0.75	0.754
**43**	0.743	0.79	0.8	0.778		0.73	0.746	0.752	0.743		0.748	0.771	0.749	0.756
**43.5**	0.745	0.792	0.802	0.780		0.732	0.748	0.751	0.744		0.75	0.774	0.753	0.759
**44**	0.747	0.794	0.803	0.781		0.735	0.75	0.754	0.746		0.753	0.778	0.754	0.762
**44.5**	0.751	0.796	0.806	0.784		0.737	0.75	0.752	0.746		0.756	0.779	0.754	0.763
**45**	0.756	0.799	0.806	0.787		0.74	0.751	0.756	0.749		0.758	0.782	0.753	0.764

**45.5**	0.757	0.797	0.804	0.786		0.741	0.752	0.757	0.750		0.76	0.783	0.754	0.766
**46**	0.76	0.797	0.807	0.788		0.743	0.754	0.757	0.751		0.763	0.786	0.758	0.769
**46.5**	0.765	0.8	0.807	0.791		0.744	0.755	0.759	0.753		0.765	0.788	0.759	0.771
**47**	0.764	0.8	0.807	0.790		0.744	0.756	0.76	0.753		0.764	0.789	0.758	0.770
**47.5**	0.77	0.803	0.81	0.794		0.747	0.757	0.761	0.755		0.767	0.79	0.761	0.773
**48**	0.77	0.803	0.809	0.794		0.748	0.759	0.762	0.756		0.768	0.792	0.762	0.774
**48.5**	0.775	0.806	0.813	0.798		0.749	0.76	0.764	0.758		0.769	0.792	0.763	0.775
**49**	0.773	0.804	0.813	0.797		0.75	0.76	0.763	0.758		0.772	0.793	0.762	0.776
**49.5**	0.773	0.807	0.813	0.798		0.752	0.762	0.765	0.760		0.773	0.794	0.767	0.778
**50**	0.775	0.81	0.814	0.800		0.752	0.764	0.767	0.761		0.774	0.795	0.765	0.778
**50.5**	0.778	0.811	0.816	0.802		0.753	0.766	0.77	0.763		0.777	0.798	0.769	0.781
**51**	0.78	0.812	0.817	0.803		0.751	0.767	0.768	0.762		0.776	0.797	0.773	0.782
**51.5**	0.785	0.815	0.814	0.805		0.754	0.769	0.771	0.765		0.779	0.8	0.775	0.785
**52**	0.783	0.813	0.814	0.803		0.754	0.77	0.773	0.766		0.78	0.801	0.777	0.786
**52.5**	0.786	0.814	0.817	0.806		0.759	0.771	0.775	0.768		0.782	0.803	0.778	0.788
**53**	0.787	0.816	0.821	0.808		0.762	0.772	0.776	0.770		0.783	0.804	0.781	0.789
**53.5**	0.787	0.815	0.82	0.807		0.762	0.774	0.779	0.772		0.785	0.806	0.781	0.791
**54**	0.788	0.815	0.82	0.808		0.763	0.775	0.779	0.772		0.785	0.807	0.78	0.791
**54.5**	0.789	0.816	0.82	0.808		0.766	0.777	0.782	0.775		0.788	0.808	0.787	0.794
**55**	0.786	0.816	0.824	0.809		0.766	0.778	0.782	0.775		0.789	0.809	0.783	0.794
**55.5**	0.79	0.817	0.824	0.810		0.77	0.779	0.785	0.778		0.79	0.812	0.79	0.797
**56**	0.793	0.819	0.828	0.813		0.771	0.781	0.784	0.779		0.792	0.814	0.788	0.798
**56.5**	0.794	0.82	0.827	0.814		0.773	0.783	0.788	0.781		0.793	0.816	0.792	0.800
**57**	0.793	0.821	0.83	0.815		0.774	0.784	0.788	0.782		0.797	0.817	0.788	0.801
**57.5**	0.795	0.823	0.829	0.816		0.775	0.785	0.79	0.783		0.797	0.816	0.793	0.802
**58**	0.795	0.821	0.83	0.815		0.776	0.787	0.791	0.785		0.799	0.819	0.795	0.804
**58.5**	0.794	0.82	0.83	0.815		0.777	0.788	0.793	0.786		0.801	0.82	0.795	0.805
**59**	0.794	0.82	0.833	0.816		0.779	0.79	0.795	0.788		0.803	0.821	0.796	0.807
**59.5**	0.795	0.821	0.835	0.817		0.78	0.791	0.795	0.789		0.803	0.823	0.796	0.807
**60**	0.798	0.822	0.835	0.818		0.782	0.793	0.796	0.790		0.806	0.825	0.801	0.811
**60.5**	0.797	0.825	0.837	0.820		0.784	0.793	0.798	0.792		0.807	0.828	0.802	0.812
**61**	0.8	0.824	0.837	0.820		0.785	0.793	0.798	0.792		0.808	0.826	0.802	0.812
**61.5**	0.799	0.825	0.838	0.821		0.786	0.795	0.8	0.794		0.81	0.828	0.803	0.814
**62**	0.803	0.827	0.84	0.823		0.787	0.797	0.803	0.796		0.812	0.829	0.807	0.816
**62.5**	0.801	0.828	0.84	0.823		0.788	0.797	0.803	0.796		0.813	0.831	0.807	0.817
**63**	0.805	0.831	0.841	0.826		0.791	0.799	0.804	0.798		0.815	0.832	0.805	0.817
**63.5**	0.804	0.83	0.843	0.826		0.792	0.8	0.807	0.800		0.816	0.834	0.809	0.820
**64**	0.803	0.831	0.843	0.826		0.793	0.801	0.808	0.801		0.818	0.836	0.81	0.821
**64.5**	0.804	0.832	0.844	0.827		0.795	0.803	0.809	0.802		0.819	0.836	0.811	0.822
**65**	0.806	0.833	0.844	0.828		0.794	0.805	0.811	0.803		0.819	0.836	0.814	0.823
**65.5**	0.806	0.833	0.848	0.829		0.796	0.805	0.812	0.804		0.823	0.838	0.814	0.825
**66**	0.803	0.833	0.848	0.828		0.799	0.807	0.812	0.806		0.823	0.84	0.816	0.826
**66.5**	0.806	0.837	0.85	0.831		0.799	0.808	0.813	0.807		0.825	0.841	0.816	0.827
**67**	0.808	0.835	0.848	0.830		0.8	0.809	0.815	0.808		0.826	0.843	0.819	0.829
**67.5**	0.806	0.835	0.849	0.830		0.801	0.81	0.817	0.809		0.828	0.842	0.82	0.830
**68**	0.815	0.84	0.853	0.836		0.804	0.812	0.819	0.812		0.83	0.844	0.823	0.832
**68.5**	0.81	0.838	0.855	0.834		0.804	0.813	0.82	0.812		0.83	0.845	0.822	0.832
**69**	0.815	0.841	0.855	0.837		0.806	0.814	0.821	0.814		0.834	0.847	0.826	0.836
**69.5**	0.816	0.837	0.854	0.836		0.805	0.816	0.822	0.814		0.833	0.847	0.824	0.835
**70**	0.814	0.841	0.856	0.837		0.807	0.815	0.824	0.815		0.834	0.848	0.828	0.837
**70.5**	0.818	0.841	0.858	0.839		0.808	0.818	0.826	0.817		0.835	0.849	0.826	0.837
**71**	0.82	0.843	0.859	0.841		0.809	0.819	0.826	0.818		0.838	0.849	0.83	0.839

**71.5**	0.818	0.839	0.86	0.839		0.81	0.818	0.826	0.818		0.838	0.849	0.829	0.839
**72**	0.818	0.839	0.86	0.839		0.812	0.821	0.829	0.821		0.84	0.851	0.831	0.841
**72.5**	0.82	0.841	0.863	0.841		0.813	0.821	0.829	0.821		0.841	0.851	0.833	0.842

Supplementary Table 6C2. Raw growth data measured for the deletion strain (Δ*s479*) at high zinc concentration. For each time point, OD_{650nm} of three biological (replicate 1-3; s479-1 to s479-3), with three technical replicates (A to C) each, are given. Additionally, the mean OD_{650nm} over all technical replicates is given for each biological replicate.

	**replicate 1**					**replicate 2**					**replicate 3**			
	s479-1A	s479-1B	**s479-1C**	**s479-1**		**s479-2A**	**s479-2B**	**s479-2C**	**s479-2**		**s479-3A**	**s479-3B**	**s479-3C**	**s479-3**
**time [h]**	OD650	OD650	OD650	**Mean OD650**		OD650	OD650	OD650	**Mean OD650**		OD650	OD650	OD650	**Mean OD650**
**0.5**	0.058	0.071	0.068	0.066		0.066	0.062	0.06	0.063		0.061	0.06	0.063	0.061
**1**	0.062	0.07	0.067	0.066		0.069	0.064	0.063	0.065		0.064	0.063	0.066	0.064
**1.5**	0.064	0.076	0.072	0.071		0.072	0.068	0.066	0.069		0.064	0.065	0.069	0.066
**2**	0.067	0.081	0.076	0.075		0.075	0.071	0.071	0.072		0.068	0.068	0.075	0.070
**2.5**	0.07	0.084	0.079	0.078		0.077	0.074	0.074	0.075		0.072	0.071	0.081	0.075
**3**	0.074	0.086	0.083	0.081		0.082	0.078	0.079	0.080		0.073	0.075	0.087	0.078
**3.5**	0.079	0.091	0.087	0.086		0.085	0.082	0.084	0.084		0.077	0.079	0.093	0.083
**4**	0.083	0.097	0.092	0.091		0.091	0.087	0.09	0.089		0.08	0.082	0.097	0.086
**4.5**	0.087	0.101	0.097	0.095		0.096	0.091	0.094	0.094		0.083	0.087	0.102	0.091
**5**	0.092	0.106	0.102	0.100		0.101	0.098	0.1	0.100		0.086	0.091	0.108	0.095
**5.5**	0.097	0.11	0.108	0.105		0.105	0.102	0.105	0.104		0.089	0.096	0.113	0.099
**6**	0.1	0.115	0.111	0.109		0.113	0.108	0.112	0.111		0.092	0.1	0.118	0.103
**6.5**	0.103	0.118	0.114	0.112		0.12	0.111	0.116	0.116		0.095	0.105	0.122	0.107
**7**	0.107	0.123	0.119	0.116		0.121	0.117	0.121	0.120		0.098	0.11	0.126	0.111
**7.5**	0.111	0.126	0.123	0.120		0.13	0.121	0.128	0.126		0.101	0.115	0.131	0.116
**8**	0.118	0.129	0.126	0.124		0.133	0.125	0.132	0.130		0.103	0.121	0.136	0.120
**8.5**	0.122	0.134	0.13	0.129		0.141	0.13	0.137	0.136		0.107	0.127	0.142	0.125
**9**	0.124	0.138	0.134	0.132		0.146	0.137	0.144	0.142		0.11	0.133	0.147	0.130
**9.5**	0.127	0.144	0.136	0.136		0.149	0.139	0.147	0.145		0.113	0.136	0.151	0.133
**10**	0.131	0.146	0.142	0.140		0.156	0.143	0.153	0.151		0.117	0.143	0.156	0.139
**10.5**	0.134	0.149	0.146	0.143		0.161	0.148	0.158	0.156		0.12	0.149	0.161	0.143
**11**	0.138	0.154	0.151	0.148		0.166	0.152	0.164	0.161		0.123	0.154	0.167	0.148
**11.5**	0.141	0.16	0.156	0.152		0.172	0.157	0.169	0.166		0.127	0.16	0.171	0.153
**12**	0.146	0.164	0.162	0.157		0.175	0.161	0.174	0.170		0.13	0.166	0.176	0.157
**12.5**	0.149	0.169	0.167	0.162		0.181	0.167	0.18	0.176		0.134	0.172	0.181	0.162
**13**	0.152	0.174	0.171	0.166		0.185	0.17	0.183	0.179		0.137	0.178	0.186	0.167
**13.5**	0.155	0.18	0.176	0.170		0.192	0.178	0.19	0.187		0.142	0.186	0.191	0.173
**14**	0.161	0.187	0.182	0.177		0.197	0.184	0.195	0.192		0.147	0.192	0.197	0.179
**14.5**	0.167	0.193	0.189	0.183		0.203	0.188	0.201	0.197		0.149	0.197	0.202	0.183
**15**	0.171	0.199	0.193	0.188		0.209	0.194	0.208	0.204		0.154	0.203	0.205	0.187
**15.5**	0.176	0.204	0.197	0.192		0.216	0.201	0.212	0.210		0.156	0.211	0.211	0.193
**16**	0.181	0.21	0.205	0.199		0.222	0.206	0.218	0.215		0.161	0.216	0.216	0.198
**16.5**	0.186	0.215	0.213	0.205		0.231	0.212	0.225	0.223		0.169	0.221	0.221	0.204
**17**	0.193	0.221	0.22	0.211		0.237	0.219	0.231	0.229		0.175	0.228	0.227	0.210
**17.5**	0.199	0.226	0.225	0.217		0.241	0.223	0.238	0.234		0.176	0.233	0.231	0.213
**18**	0.204	0.23	0.231	0.222		0.251	0.229	0.243	0.241		0.186	0.239	0.236	0.220
**18.5**	0.208	0.235	0.237	0.227		0.256	0.235	0.25	0.247		0.192	0.245	0.241	0.226
**19**	0.219	0.24	0.24	0.233		0.262	0.241	0.256	0.253		0.198	0.251	0.246	0.232
**19.5**	0.226	0.246	0.246	0.239		0.267	0.248	0.261	0.259		0.204	0.257	0.251	0.237
**20**	0.233	0.251	0.249	0.244		0.276	0.255	0.268	0.266		0.211	0.264	0.257	0.244
**20.5**	0.247	0.256	0.254	0.252		0.283	0.262	0.275	0.273		0.218	0.27	0.262	0.250

**21**	0.257	0.264	0.261	0.261		0.288	0.267	0.281	0.279		0.224	0.276	0.267	0.256
**21.5**	0.265	0.272	0.267	0.268		0.294	0.271	0.286	0.284		0.228	0.282	0.273	0.261
**22**	0.275	0.278	0.271	0.275		0.301	0.277	0.294	0.291		0.234	0.289	0.278	0.267
**22.5**	0.278	0.287	0.277	0.281		0.307	0.284	0.299	0.297		0.239	0.295	0.283	0.272
**23**	0.284	0.296	0.283	0.288		0.314	0.29	0.306	0.303		0.244	0.302	0.288	0.278
**23.5**	0.286	0.305	0.29	0.294		0.32	0.296	0.313	0.310		0.248	0.308	0.294	0.283
**24**	0.289	0.314	0.299	0.301		0.326	0.301	0.318	0.315		0.255	0.314	0.3	0.290
**24.5**	0.292	0.323	0.308	0.308		0.334	0.307	0.326	0.322		0.26	0.319	0.305	0.295
**25**	0.298	0.335	0.319	0.317		0.339	0.313	0.332	0.328		0.266	0.326	0.31	0.301
**25.5**	0.306	0.346	0.329	0.327		0.346	0.319	0.338	0.334		0.271	0.332	0.315	0.306
**26**	0.319	0.357	0.342	0.339		0.354	0.325	0.345	0.341		0.278	0.34	0.319	0.312
**26.5**	0.337	0.367	0.355	0.353		0.362	0.332	0.351	0.348		0.284	0.346	0.327	0.319
**27**	0.352	0.379	0.367	0.366		0.37	0.337	0.357	0.355		0.289	0.353	0.331	0.324
**27.5**	0.359	0.39	0.381	0.377		0.378	0.343	0.363	0.361		0.296	0.36	0.338	0.331
**28**	0.369	0.405	0.395	0.390		0.387	0.351	0.37	0.369		0.303	0.368	0.343	0.338
**28.5**	0.38	0.417	0.409	0.402		0.394	0.356	0.375	0.375		0.311	0.375	0.35	0.345
**29**	0.389	0.429	0.42	0.413		0.404	0.362	0.383	0.383		0.32	0.385	0.357	0.354
**29.5**	0.399	0.442	0.433	0.425		0.414	0.37	0.387	0.390		0.331	0.392	0.363	0.362
**30**	0.408	0.455	0.444	0.436		0.422	0.376	0.394	0.397		0.34	0.399	0.37	0.370
**30.5**	0.416	0.465	0.454	0.445		0.433	0.383	0.399	0.405		0.352	0.407	0.376	0.378
**31**	0.426	0.473	0.466	0.455		0.441	0.389	0.406	0.412		0.365	0.414	0.384	0.388
**31.5**	0.438	0.484	0.473	0.465		0.45	0.397	0.412	0.420		0.375	0.421	0.391	0.396
**32**	0.466	0.492	0.484	0.481		0.46	0.407	0.418	0.428		0.385	0.428	0.398	0.404
**32.5**	0.476	0.499	0.493	0.489		0.467	0.413	0.426	0.435		0.396	0.434	0.406	0.412
**33**	0.48	0.507	0.503	0.497		0.473	0.419	0.432	0.441		0.405	0.441	0.411	0.419
**33.5**	0.509	0.515	0.512	0.512		0.481	0.427	0.44	0.449		0.415	0.448	0.421	0.428
**34**	0.522	0.523	0.52	0.522		0.49	0.438	0.449	0.459		0.426	0.453	0.428	0.436
**34.5**	0.524	0.529	0.528	0.527		0.493	0.443	0.456	0.464		0.435	0.459	0.435	0.443
**35**	0.537	0.537	0.538	0.537		0.5	0.454	0.465	0.473		0.446	0.464	0.444	0.451
**35.5**	0.546	0.545	0.546	0.546		0.505	0.464	0.473	0.481		0.455	0.471	0.45	0.459
**36**	0.555	0.554	0.553	0.554		0.513	0.473	0.481	0.489		0.465	0.474	0.461	0.467
**36.5**	0.56	0.561	0.561	0.561		0.516	0.481	0.488	0.495		0.474	0.48	0.467	0.474
**37**	0.567	0.566	0.568	0.567		0.521	0.488	0.497	0.502		0.48	0.487	0.476	0.481
**37.5**	0.572	0.579	0.576	0.576		0.527	0.496	0.506	0.510		0.49	0.491	0.483	0.488
**38**	0.577	0.584	0.581	0.581		0.532	0.505	0.513	0.517		0.495	0.495	0.491	0.494
**38.5**	0.585	0.594	0.589	0.589		0.54	0.511	0.519	0.523		0.499	0.501	0.496	0.499
**39**	0.591	0.605	0.596	0.597		0.542	0.519	0.527	0.529		0.51	0.505	0.504	0.506
**39.5**	0.589	0.61	0.602	0.600		0.549	0.526	0.533	0.536		0.518	0.51	0.513	0.514
**40**	0.59	0.617	0.61	0.606		0.551	0.531	0.538	0.540		0.513	0.516	0.518	0.516
**40.5**	0.59	0.619	0.617	0.609		0.555	0.538	0.544	0.546		0.518	0.523	0.525	0.522
**41**	0.601	0.624	0.625	0.617		0.555	0.543	0.55	0.549		0.525	0.529	0.528	0.527
**41.5**	0.606	0.635	0.632	0.624		0.561	0.547	0.552	0.553		0.539	0.533	0.532	0.535
**42**	0.61	0.642	0.637	0.630		0.564	0.549	0.554	0.556		0.541	0.537	0.537	0.538
**42.5**	0.604	0.645	0.643	0.631		0.567	0.554	0.559	0.560		0.547	0.541	0.541	0.543
**43**	0.61	0.655	0.649	0.638		0.573	0.559	0.564	0.565		0.558	0.546	0.545	0.550
**43.5**	0.608	0.654	0.653	0.638		0.577	0.562	0.567	0.569		0.568	0.55	0.549	0.556
**44**	0.611	0.651	0.664	0.642		0.584	0.567	0.57	0.574		0.567	0.55	0.553	0.557
**44.5**	0.615	0.66	0.666	0.647		0.586	0.571	0.574	0.577		0.574	0.557	0.557	0.563
**45**	0.63	0.668	0.677	0.658		0.59	0.573	0.577	0.580		0.582	0.56	0.561	0.568
**45.5**	0.635	0.669	0.679	0.661		0.595	0.579	0.581	0.585		0.587	0.566	0.564	0.572

**46**	0.64	0.673	0.685	0.666		0.598	0.583	0.584	0.588		0.591	0.571	0.568	0.577
**46.5**	0.652	0.677	0.69	0.673		0.599	0.586	0.586	0.590		0.597	0.576	0.573	0.582
**47**	0.65	0.676	0.693	0.673		0.603	0.59	0.587	0.593		0.6	0.579	0.574	0.584
**47.5**	0.666	0.681	0.696	0.681		0.608	0.593	0.591	0.597		0.606	0.583	0.579	0.589
**48**	0.67	0.685	0.698	0.684		0.608	0.596	0.594	0.599		0.609	0.588	0.582	0.593
**48.5**	0.674	0.689	0.702	0.688		0.612	0.6	0.596	0.603		0.619	0.59	0.585	0.598
**49**	0.674	0.694	0.703	0.690		0.614	0.601	0.598	0.604		0.617	0.594	0.588	0.600
**49.5**	0.683	0.694	0.703	0.693		0.616	0.603	0.6	0.606		0.621	0.598	0.59	0.603
**50**	0.675	0.692	0.705	0.691		0.619	0.604	0.603	0.609		0.623	0.6	0.593	0.605
**50.5**	0.68	0.693	0.707	0.693		0.622	0.604	0.605	0.610		0.627	0.603	0.596	0.609
**51**	0.681	0.7	0.708	0.696		0.632	0.608	0.606	0.615		0.633	0.604	0.599	0.612
**51.5**	0.688	0.701	0.712	0.700		0.631	0.612	0.609	0.617		0.637	0.606	0.602	0.615
**52**	0.687	0.7	0.713	0.700		0.635	0.612	0.611	0.619		0.638	0.61	0.604	0.617
**52.5**	0.691	0.702	0.717	0.703		0.636	0.615	0.612	0.621		0.644	0.612	0.606	0.621
**53**	0.689	0.703	0.716	0.703		0.637	0.616	0.615	0.623		0.644	0.614	0.609	0.622
**53.5**	0.691	0.704	0.716	0.704		0.641	0.621	0.617	0.626		0.648	0.615	0.612	0.625
**54**	0.693	0.706	0.721	0.707		0.646	0.623	0.619	0.629		0.648	0.621	0.614	0.628
**54.5**	0.696	0.706	0.721	0.708		0.646	0.623	0.621	0.630		0.655	0.619	0.616	0.630
**55**	0.692	0.707	0.722	0.707		0.648	0.629	0.623	0.633		0.658	0.624	0.618	0.633
**55.5**	0.701	0.714	0.725	0.713		0.652	0.629	0.624	0.635		0.666	0.624	0.621	0.637
**56**	0.706	0.714	0.728	0.716		0.653	0.634	0.627	0.638		0.668	0.628	0.624	0.640
**56.5**	0.705	0.718	0.73	0.718		0.657	0.634	0.629	0.640		0.67	0.628	0.626	0.641
**57**	0.709	0.722	0.73	0.720		0.66	0.637	0.631	0.643		0.678	0.63	0.629	0.646
**57.5**	0.704	0.721	0.732	0.719		0.661	0.639	0.632	0.644		0.674	0.635	0.63	0.646
**58**	0.708	0.723	0.733	0.721		0.665	0.641	0.633	0.646		0.673	0.637	0.633	0.648
**58.5**	0.707	0.725	0.733	0.722		0.668	0.642	0.635	0.648		0.676	0.637	0.635	0.649
**59**	0.709	0.725	0.732	0.722		0.669	0.642	0.637	0.649		0.677	0.642	0.638	0.652
**59.5**	0.711	0.726	0.734	0.724		0.671	0.644	0.639	0.651		0.677	0.644	0.64	0.654
**60**	0.718	0.733	0.74	0.730		0.673	0.647	0.641	0.654		0.686	0.645	0.641	0.657
**60.5**	0.716	0.732	0.736	0.728		0.675	0.648	0.643	0.655		0.684	0.647	0.644	0.658
**61**	0.715	0.733	0.74	0.729		0.674	0.649	0.644	0.656		0.684	0.649	0.645	0.659
**61.5**	0.712	0.732	0.739	0.728		0.679	0.652	0.645	0.659		0.683	0.651	0.647	0.660
**62**	0.722	0.736	0.742	0.733		0.681	0.654	0.649	0.661		0.689	0.652	0.649	0.663
**62.5**	0.716	0.736	0.739	0.730		0.679	0.655	0.649	0.661		0.7	0.655	0.651	0.669
**63**	0.719	0.741	0.745	0.735		0.683	0.657	0.651	0.664		0.702	0.655	0.652	0.670
**63.5**	0.722	0.734	0.744	0.733		0.684	0.658	0.653	0.665		0.698	0.659	0.654	0.670
**64**	0.726	0.737	0.744	0.736		0.684	0.66	0.653	0.666		0.692	0.659	0.657	0.669
**64.5**	0.723	0.744	0.746	0.738		0.685	0.662	0.655	0.667		0.698	0.662	0.658	0.673
**65**	0.725	0.743	0.746	0.738		0.683	0.663	0.655	0.667		0.7	0.663	0.66	0.674
**65.5**	0.73	0.74	0.743	0.738		0.689	0.664	0.658	0.670		0.705	0.666	0.661	0.677
**66**	0.729	0.744	0.748	0.740		0.688	0.666	0.658	0.671		0.704	0.665	0.663	0.677
**66.5**	0.732	0.746	0.746	0.741		0.689	0.669	0.661	0.673		0.705	0.668	0.665	0.679
**67**	0.734	0.748	0.752	0.745		0.691	0.669	0.66	0.673		0.713	0.67	0.666	0.683
**67.5**	0.733	0.749	0.75	0.744		0.691	0.671	0.662	0.675		0.717	0.671	0.668	0.685
**68**	0.741	0.747	0.75	0.746		0.698	0.672	0.664	0.678		0.708	0.673	0.67	0.684
**68.5**	0.741	0.748	0.747	0.745		0.7	0.674	0.665	0.680		0.715	0.675	0.671	0.687
**69**	0.739	0.753	0.754	0.749		0.696	0.675	0.666	0.679		0.723	0.675	0.672	0.690
**69.5**	0.737	0.751	0.752	0.747		0.697	0.677	0.668	0.681		0.726	0.677	0.674	0.692
**70**	0.74	0.752	0.754	0.749		0.698	0.677	0.668	0.681		0.72	0.68	0.676	0.692
**70.5**	0.745	0.749	0.756	0.750		0.698	0.68	0.669	0.682		0.724	0.681	0.678	0.694
**71**	0.747	0.752	0.76	0.753		0.7	0.681	0.673	0.685		0.729	0.683	0.68	0.697
**71.5**	0.748	0.751	0.76	0.753		0.702	0.681	0.672	0.685		0.731	0.683	0.681	0.698

**72**	0.752	0.753	0.759	0.755		0.703	0.683	0.674	0.687		0.736	0.685	0.683	0.701
**72.5**	0.755	0.755	0.764	0.758		0.704	0.684	0.675	0.688		0.737	0.685	0.685	0.702
